# Analysis of shoulder compressive and shear forces during functional activities of daily life

**DOI:** 10.1016/j.clinbiomech.2018.03.006

**Published:** 2018-05

**Authors:** Christian Klemt, Joe A. Prinold, Sharon Morgans, Samuel H.L. Smith, Daniel Nolte, Peter Reilly, Anthony M.J. Bull

**Affiliations:** a Department of Bioengineering, Imperial College London, London, SW7 2AZ, United Kingdom; b Physiotherapy Unit, Imperial College Healthcare NHS Trust, London, NW8 9NH, United Kingdom; c Department of Trauma and Orthopaedics, Imperial College Healthcare NHS Trust, London, W2 1NY, United Kingdom

**Keywords:** Shoulder, Contact forces, Activities of daily living, Musculoskeletal modelling, Implant design, Rehabilitation planning

## Abstract

**Background:**

Knowledge of forces acting through the glenohumeral joint during activities of daily living is a prerequisite for improving implant design and aiding rehabilitation planning. Existing data are limited by the number of activities performed and, in some cases, the lack of representation of the glenohumeral loading direction, although high shear force components may cause joint dislocation or implant loosening. This study aims to analyse shoulder compression and shear force components during essential functional activities of daily living.

**Methods:**

This is a combined modelling and experimental study. Motion data and external forces measured from 25 participants for 26 activities of daily living serve as input into an upper limb musculoskeletal model that quantifies glenohumeral loading.

**Findings:**

The shoulder contact force exceeds 50% of the body weight in 10/26 activities of daily living with a maximum contact force of 164% of the body weight (SD 69%) for a sit to stand task. The ratio of glenohumeral shear force component to compression force component exceeds 0.5 in 8/26 functional activities, with maximum ratios for reaching across the body (1.09; SD 0.41) and pick and place an everyday object (0.88; SD 0.36).

**Interpretation:**

This study demonstrates substantial loads through the glenohumeral joint during activities of daily living. The ratios of glenohumeral shear force component to compression force component are considerable when high loads act at long lever arms and at high angles of arm elevation. These glenohumeral ratios represent a key component of loading that should be considered when designing implants, surgical procedures, or rehabilitation protocols.

## Highlights

• Musculoskeletal shoulder modelling
• Activities of daily living
• Glenohumeral joint forces
• Rehabilitation planning
• Implant design

## 1 Introduction

The forces at the glenohumeral joint are frequently dismissed as being small when compared to loads at the knee and hip joint (Poppen and Walker, 1978). This is despite the fact that substantial loads through the joint have been demonstrated during activities of daily living (ADL) with either instrumented shoulder implants or musculoskeletal shoulder models (Bergmann et al., 2007; Nikooyan et al., 2010; Van Andel et al., 2008; Veeger et al., 2006; Westerhoff et al., 2009). Anglin et al. (2000) have reported glenohumeral contact forces (GHCFs) of 240% of the body weight (BW) for lifting a 10 kg suitcase laterally, 180% BW for holding a 5 kg box ventrally and 170% BW on average for walking with a cane. Bergmann et al. (2007) found GHCFs of 70% BW for hammering a nail, 65% BW for hair combing and 40% BW for steering a car, while Charlton and Johnson (2006) reported GHCFs ranging from 23% to 75% BW for 10 functional activities including feeding, personal hygiene and lifting everyday objects.

The GHCFs are achieved through compression of the humeral head into the glenoid-labral concavity through contraction of muscles surrounding the shoulder, and the joint force can be decomposed into 3 components: compressive force, anterior-posterior shear force and superior-inferior shear force (Lee et al., 2000). The compressive force component is directed to the centre of the glenoid socket, while the shear force components destabilise the joint by translating the humeral head towards the glenoid rim, with the ratio of these force components determining the risk of joint luxation and the loading of the capsuloligamentous labral complex as well as the prosthesis-bone interface in shoulder arthroplasties (Klemt et al., 2017, Nishinaka et al., 2008, Lazarus et al., 1996).

Therefore, the precise understanding of the magnitude of glenohumeral shear force component to compression force component during functional activities of daily life is essential to aid rehabilitation planning for patients post shoulder surgery, with these data enabling the assessment of existing rehabilitation strategies, the development of novel physiotherapy and strength training programmes as well as advice to be given to patients to avoid overloading of the joint following surgical intervention, such as a Bankart repair (Arciero et al., 1994, Hayes et al., 2004). Furthermore, knowledge of glenohumeral shear force component to compression force component allows preclinical test procedures for shoulder arthroplasties to be designed to improve implant design as well as glenoid implant fixation, where off-centre loading is considered the major cause of loosening (Geraldes et al., 2017). These data also provide support for the design and testing of preclinical surgical procedures including tendon transfer surgeries (Ackland and Pandy, 2009.).

Despite several studies being reported in the literature, there is currently no detailed knowledge of glenohumeral contact forces during functional activities with existing studies focusing on a small number of functional activities without always presenting the loading direction on the joint. Therefore, the aim of this study is to analyse the compression and shear force components of the glenohumeral contact force during essential activities of daily living which may ultimately aid implant design, surgical intervention and shoulder rehabilitation.

## 2 Methods

### 2.1 Subjects

Twenty-five healthy right-handed volunteers (20 males, 5 females) with no history of shoulder pathology participated in this study. Each activity was performed by a subset of volunteers (Table 1). Informed consent was obtained from each subject and ethical approval was granted by the Imperial College Research Ethics Committee.

**Table 1:** Participant information for each set of functional activities. Data are presented as mean and standard deviation (SD).

Dataset name	Participants	Age (years)	Height (m)	Body mass (kg)
ADL1	8	34.4 (SD 13.9)	1.73 (SD 0.08)	69.3 (SD 13.9)
ADL2	6	27.1 (SD 1.26)	1.77 (SD 0.09)	75.8 (SD 5.2)
Driving	4	26.0 (SD 1.41)	1.76 (SD 0.13)	67.5 (SD 13.9)
Planar tasks	7	25.4 (SD 1.13)	1.82 (SD 0.07)	75.0 (SD 6.1)

### 2.2 Functional activities

The volunteers were instructed to perform 26 functional activities of daily life with three sets per activity (Table 2). These activities include basic functional activities of daily life such as feeding, personal hygiene, mobility and lifting everyday objects, alongside activities with larger range of motion such as planar movements. The activities were selected based on Charlton and Johnson (2006), Bergmann et al. (2007), Westerhoff et al. (2009), Coffey and McCarthy (2013), and Pandis et al. (2015). Thirty seconds' rest was enforced between sets.

**Table 2:** Activities of daily living within each dataset.

Dataset name	Activity	Loading
ADL1	Reach back of head	–
	Lift block to head height	0.5 kg
	Lift block to shoulder height	0.5 kg
	Brush left side of head	–
	Clean back	–
	Drink from mug	–
	Eat with hand	–
	Eat with spoon	–
	Lift shopping bag from floor (standing)	2 kg
	Lift shopping bag from floor (seated)	2 kg
	Reach opposite axilla	–
	Perineal care (reach back pocket)	–
	Reach far ahead	0.5 kg
	Sit to stand	Load cell
ADL2	Extreme (reach across body)	–
	Pick and place (short distance)	2 kg
	Pull	–
	Push	–
Driving	Fast (right and left turn)	Load cell
	Slow (right and left turn)	Load cell
Planar tasks	Abduction (slow)	–
	Abduction (fast)	–
	Forward flexion (slow)	–
	Forward flexion (fast)	–

**Fig. 1:**
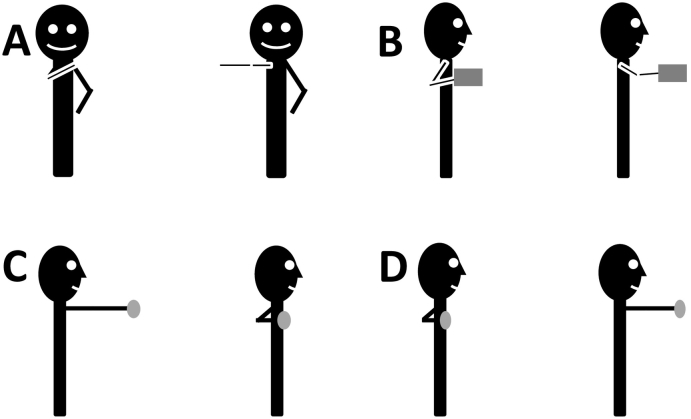
Representation of the activities of the ADL2 dataset. (A) Reach across the body, (B) Pick and place, (C) Pull, (D) Push.

The ADL2 activity of “Extreme” involves moving the hand to a point furthest from the shoulder, across the body and in the transverse plane, level with the glenohumeral joint (Fig. 1A). The “Pick and place” activity involves moving an object approximately 30 cm away from the chest within the transverse plane, after an initial starting point close to the body (Fig. 1B). The “Pull” activity involves the subject starting with outstretched arms, holding a thin wooden rod horizontally, and moving the arms in as far as possible. This is performed at chest height (Fig. 1C). The “Push” activity is the opposing action (Fig. 1D).

The “Driving” activity involves moving a steering wheel with both hands until the wheel has rotated by 65° clockwise and anticlockwise for “driving right” and “driving left” respectively. The activity was performed at both low (12 mph) and high speeds (24 mph). The torque resistance on the wheel was set to 4 Nm to simulate a standard driving torque (Li and Xian (2013).

### 2.3 Protocol

Kinematic data collection was performed using a 10-camera optical motion tracking system (Vicon Motion Tracking System, Oxford, UK) at 100 Hz and three force plates (Kistler Instrument Corp., Winterthur, Switzerland) at 1000 Hz. The “Driving” activity was performed using the driving simulator as published in Pandis et al. (2015), while the “Sit to stand” task was conducted using an instrumented chair as described in Duffell et al. (2013).

A scapula tracker (ST) was used to measure scapula kinematics (Prinold et al., 2011). The ST consists of a base attached to the mid-portion of the scapula spine and an adjustable foot positioned on the meeting-point between acromion process and scapula spine (Shaheen et al., 2011). The ST technical coordinate frame was calibrated with the anatomical coordinate frame of the scapula using the International Society of Biomechanics (ISB) recommended landmarks and measured directly using a scapula locator (Shaheen et al., 2011). Calibration was performed at 90° of humerothoracic elevation, 45° to the coronal plane. The calibration transformation was applied to each trial of that participant with errors from static palpation of landmarks being small (De Groot, 1997). The scapula kinematics for the functional activity “driving” was derived from regression equations based on the humerothoracic position (Charlton and Johnson, 2006).

Twenty-one retro-reflective markers were used to track the thorax, scapula, clavicle, humerus, radius and ulna (Shaheen et al., 2011; Wu et al., 2005). The elbow epicondyles were defined as a rigid offset from the humerus technical frame with the arm at 90° of humerothoracic flexion, 45° from the coronal plane, 90° elbow flexion and a vertical forearm. Least-square fitting was used to calculate the glenohumeral head rotational centre during a functional task using the Locator to track the scapula (Gamage and Lasemby, 2002).

A low-pass fourth-order Butterworth filter (cut-off 4.7 Hz) was used to remove noise from the kinematic data, whilst the force plate data were processed with a low-pass fourth-order Butterworth filter (cut-off 10 Hz) after spectral analysis of the signal (Prinold and Bull, 2016).

The orientation of the upper limb joints in the 3D Euclidean space was calculated using Euler angles with z-x′-y″ Cardan Sequence (Prinold and Bull, 2016). For the glenohumeral joint, the rotations about z, y, and x-axes are forward flexion/extension, external/internal rotation and abduction/adduction respectively (Wu et al., 2005).

### 2.4 Modelling and analysis

**Fig. 2:**
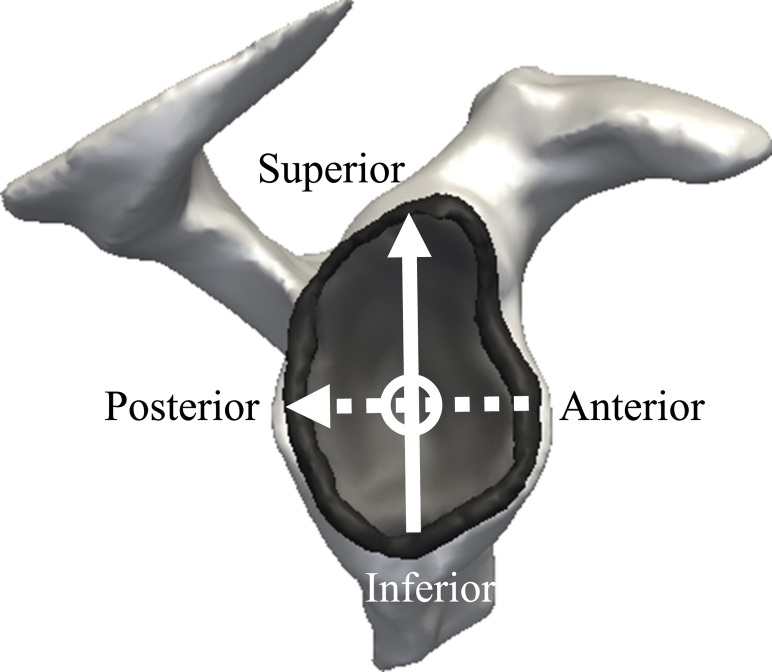
Components of glenohumeral joint force in the glenoid coordinate frame. The superior-inferior shear force is represented by the solid arrow, the posterior-anterior shear force is represented by the dashed arrow, and the glenohumeral compression force is represented by the circle.

The motion data and external forces served as inputs into the UK National Shoulder Model (UK NSM; as described in Charlton and Johnson, 2006) which was used to model glenohumeral contact forces in the right shoulder. The model is an inverse dynamics musculoskeletal model of the upper limb which includes 87 muscle elements and 3 ligaments crossing five functional joints (sternoclavicular, acromioclavicular, scapulathoracic, glenohumeral and elbow). Model validation was previously performed by comparison to instrumented implant measurements (Bergmann et al., 2007) and electromyography for functional activities with a similar range of motion compared to the functional tasks of this study (Charlton and Johnson, 2006; Prinold et al., 2013). Intersegmental moments are calculated using the measured kinematics and an optimisation algorithm minimises the sum of muscle stresses squared to solve the natural muscle load-sharing redundancy. Subject-specific measurements, including segment lengths and body weight and height, are used for scaling body segment parameters (Johnson et al., 1996). A partially closed chain method is used to optimise scapula and clavicle kinematics (Prinold and Bull, 2014). The model outputs include glenohumeral contact force and joint angles. The glenohumeral contact forces are represented in the anatomical coordinate frame of the glenoid plane as described in Lee and Lee (2010), which allows the decomposition of the joint reaction force in 3 components: compressive force, anterior-posterior shear force and superior-inferior shear force (Fig. 2).

### 2.5 Data normalisation

Each dataset was normalised to allow averaging and clear presentation of results. Once the 0%, 100%, and in some cases the 50% points were established the data was then interpolated between these points using a cubic spline function.

The activities in the ADL1 dataset were normalised according to the points described in Table 3. In some cases, the activities were split into two phases, where there was a clear distinction between the end of one phase (start point to functional position)and the start of the second phase (return from functional position to start point), resulting in two values being presented at 50% with a discontinuity in the results in some cases.

**Table 3:** Points used to normalise the activities in the ADL1 dataset.

Activity	Starting to functional position		Functional to starting position	
	0%	50%	50%	100%
Reach back of head	Initiation of movement	Wrist reaches furthest point	Initiation of return movement	Wrist returned to steady distance
Lift block to head height	Force exerted to lift block	Block released	Force exerted to lift block	Block released
Lift block to shoulder height	Force exerted to lift block	Block released	Force exerted to lift block	Block released
Brush left side of head	Initiation of movement	Wrist reaches furthest point	Initiation of return movement	Wrist returned to steady distance
Clean back	Initiation of movement	Wrist reaches furthest point	Initiation of return movement	Wrist returned to steady distance
Drink from mug	Force exerted to lift mug	Wrist reaches furthest point	Force exerted to lift block	Wrist returned to steady distance
Eat with hand	Initiation of movement	Wrist reaches furthest point	Initiation of return movement	Wrist returned to steady distance
Eat with spoon	Initiation of movement	Wrist reaches furthest point	Initiation of return movement	Wrist returned to steady distance
Lift shopping bag from floor (standing)	Force exerted to lift bag	Wrist reaches furthest point	Initiation of return movement	Bag released
Lift shopping bag from floor (seated)	Force exerted to lift bag	Bag released	Force exerted to lift bag	Bag released
Reach opposite axilla	Initiation of movement	Wrist reaches furthest point	Initiation of return movement	Wrist returned to steady distance
Perineal care (reach back pocket)	Initiation of movement	Wrist reaches furthest point	Initiation of return movement	Wrist returned to steady distance
Reach far ahead	Force exerted to lift block	Block released	Force exerted to lift block	Block released
Sit to stand (to sit)	Force applied to chair arms	Force returns to baseline	Force applied to chair arms	Force returns to baseline

To normalise the ADL2 dataset, the distance between the mid-point of the two wrist markers from their position in the first frame was calculated. The initiation of the movement in the desired direction, as judged from the distance time graph, was set as 0% and the furthest point at which the hand stopped appreciably moving in the desired direction as 100% (push, extreme), or vice versa as appropriate (pull, pick and place). This was defined visually per movement using the motion data.

The speed and angle of the wheel were used to normalise the “Driving” data. An average vector between two markers on the appropriate side of the wheel's handle (right for a right turn and left for a left turn) was found over the first five frames. The angle from that vector was then found for each frame. The first frame in which the velocity of the angle went over 0.04°/s was used as 0%. The point at which the angle went over 60° was set as the 100% point.

The “Planar” data was normalised to humerothoracic elevation angle, using a y-z’-y” Euler angle sequence. The largest value common to all subjects and trials at the bottom of the two phases of the motion (start of upward phase and end of downward phase) were used as the start and end points. The two 50% points were then defined as the smallest values common to all subjects and trials at the end of the upward motion phase and the start of the downward phase. These two phases were interpolated separately.

## 3 Results

The glenohumeral contact forces range from 26% (SD 7%) to 164% (SD 69%) of the body weight (BW) for the 26 functional activities of daily living (Table 4).

**Table 4:** Glenohumeral contact forces for 26 functional activities of daily living. Data are presented as mean and standard deviation (SD).

	Glenohumeral Contact Force [% BW]	Ratio of glenohumeral superior (+) – inferior (−) shear to compression force	Ratio of glenohumeral posterior (+) – anterior (−) shear to compression force
Reach back of head	33 (SD 8)	0.13 (SD 0.06)	−0.24 (SD 0.11)
Lift block to head height	55 (SD 18)	0.09 (SD 0.03)	−0.42 (SD 0.14)
Lift block to shoulder height	52 (SD 15)	0.10 (SD 0.04)	−0.40 (SD 0.16)
Brush left side of head	35 (SD 16)	0.12 (SD 0.07)	−0.52 (SD 0.19)
Clean back	39 (SD 14)	−0.57 (SD 0.27)	−0.16 (SD 0.07)
Drink from mug	29 (SD 9)	0.08 (SD 0.02)	−0.09 (SD 0.04)
Eat with hand	26 (SD 7)	0.13 (SD 0.03)	−0.14 (SD 0.09)
Eat with spoon	32 (SD 8)	0.09 (SD 0.02)	−0.16 (SD 0.08)
Lift shopping bag from floor	53 (SD 15)	−0.32 (SD 0.14)	−0.21 (SD 0.10)
Lift shopping bag on lap	69 (SD 22)	−0.30 (SD 0.13)	−0.27 (SD 0.12)
Reach opposite axilla	24 (SD 12)	0.17 (SD 0.09)	−0.52 (SD 0.24)
Perineal care	29 (SD 16)	−0.58 (SD 0.36)	−0.25 (SD 0.11)
Reach far ahead	52 (SD 24)	0.19 (SD 0.06)	−0.25 (SD 0.12)
Sit to stand	164 (SD 69)	0.41 (SD 0.20)	−0.50 (SD 0.22)
Driving slow right	35 (SD 11)	0.05 (SD 0.02)	−0.23 (SD 0.10)
Driving slow left	45 (SD 13)	0.06 (SD 0.03)	−0.20 (SD 0.07)
Driving fast right	33 (SD 9)	0.02 (SD 0.01)	−0.19 (SD 0.05)
Driving fast left	47 (SD 16)	0.09 (SD 0.04)	−0.23 (SD 0.08)
Extreme	48 (SD 24)	−1.09 (SD 0.41)	−0.62 (SD 0.25)
Pick and place	37 (SD 14)	0.88 (SD 0.36)	−0.84 (SD 0.34)
Pull	38 (SD 13)	−0.77 (SD 0.30)	−0.14 (SD 0.06)
Push	38 (SD 16)	−0.81 (SD 0.33)	−0.19 (SD 0.07)
Abduction slow	58 (SD 15)	0.28 (SD 0.09)	−0.23 (SD 0.08)
Abduction fast	54 (SD 17)	0.30 (SD 0.14)	−0.18 (SD 0.08)
Flexion slow	54 (SD 13)	0.13 (SD 0.05)	−0.19 (SD 0.06)
Flexion fast	51 (SD 14)	0.14 (SD 0.06)	−0.14 (SD 0.05)

The ratio of glenohumeral shear force component to compression force component exceeds 0.5 in 8/26 functional activities (Table 4). The glenohumeral ratio ranges from 0.50 (SD 0.22) to 1.09 (SD 0.41) for activities such as reaching across the body, pushing and pulling, picking and placing an everyday object as well as sit to stand.

**Fig. 3:**
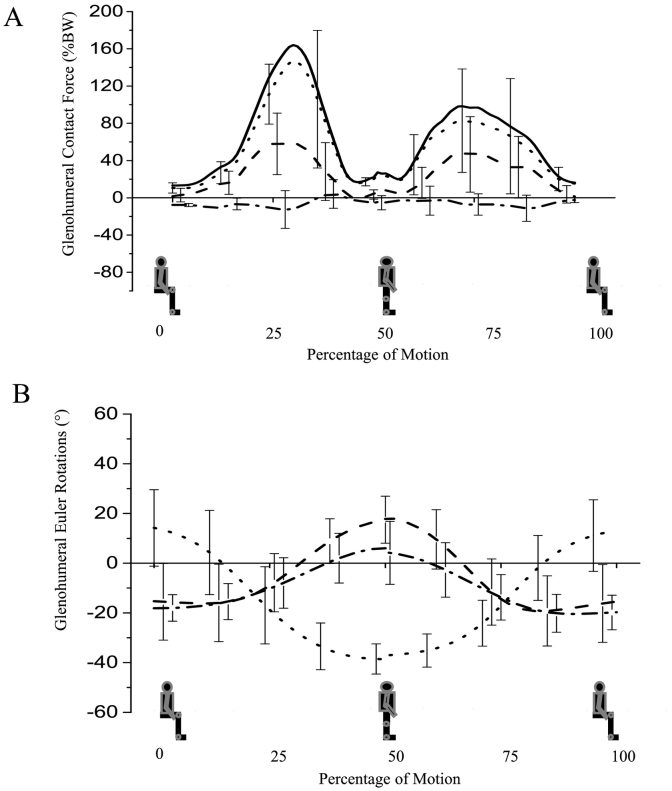
(A) Glenohumeral contact forces and (B) glenohumeral Euler rotations during “sit to stand” activity. (A) The solid line represents the total joint contact force. The dotted line represents the joint compressive force, the dashed line represents superior (+) – inferior (−) shear, the dashed and dotted line represents posterior (+) – anterior (−) shear. (B) The dotted line represents (+) flexion, the dashed line represents (+) abduction and the dashed and dotted line represents (+) external rotation. Bars represent standard deviations.

The glenohumeral contact force exceeds 164% BW (SD 69%) for the sit to stand task, with the ratio of anterior shear force component to compression force component being 0.50 (SD 0.22). The superior glenohumeral ratio exceeds 0.41 (SD 0.20), representing the second largest superior glenohumeral ratio of the entire dataset (Fig. 3).

**Fig. 4:**
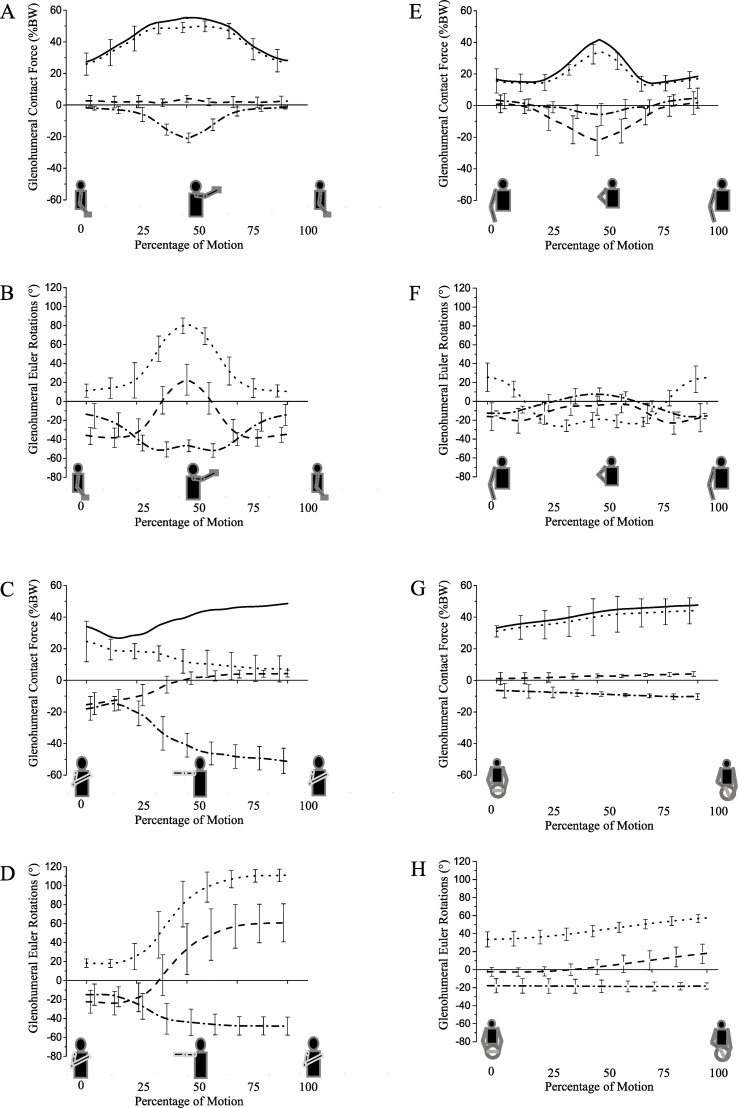
Glenohumeral contact forces during (A) “Lifting block to head height”, (C) “Reaching across the body”, (E) “Cleaning back”, (G) “Drive fast left”. The solid line represents the total joint contact force. The dotted black line represents the joint compressive force, the dashed line represents superior (+) – inferior (−) shear, the dashed and dotted line represents posterior (+) – anterior (−) shear. Glenohumeral Euler rotations during (B) “Lifting block to head height”, (D) “Reaching across the body”, (F) “Cleaning back”, (H) “Drive fast left”. The dotted line represents (+) flexion, the dashed line represents (+) abduction and the dashed and dotted line represents (+) external rotation. Bars represent standard deviations.

The glenohumeral contact force ranges from 37% (SD 14%) to 55% (SD 18%) BW for functional activities of lifting and placing everyday objects to shoulder/head height (Fig. 4A). The ratios of anterior shear force component to compression force component are the largest of the dataset, ranging from 0.40 (SD 0.16) to 0.88 (SD 0.36).

The glenohumeral contact force exceeds 48% BW (SD 24%) for the task “reaching across the body”. The ratio of anterior shear force component to compression force component is 0.62 (SD 0.25), while the inferior glenohumeral ratio exceeds 1.09 (SD 0.41), thereby representing the largest ratio of inferior shear force component to compression force component (Fig. 4B).

The glenohumeral contact forces for feeding tasks are below 32% BW (SD 8%). The glenohumeral ratios during these activities are small ranging between 0.09 (SD 0.04) and 0.14 (SD 0.09) for the anterior ratio, while the superior glenohumeral ratio ranges from 0.08 (SD 0.02) to 0.13 (SD 0.03). In contrast, the glenohumeral contact forces for tasks involving personal hygiene are below 39% BW (SD 14), with anterior glenohumeral ratios exceeding 0.25 (SD 0.11) (Fig. 4C).

The glenohumeral contact forces during the “driving” activity range from 33% (SD 9%) to 47% BW (SD 16%). The ratio of anterior shear force component to compression force component does not exceed 0.23 (SD 0.10), while the superior glenohumeral ratio is below 0.09 (SD 0.04), thereby representing the lowest ratio from the 26 activities of daily living (Fig. 4D).

## 4 Discussion

### 4.1 Glenohumeral contact forces to aid implant design

In this study, glenohumeral shear force components and compression force components during 26 functional activities of daily life were analysed to provide detailed insight into the loading of the joint. The results show that substantial loads are exerted across the joint even during basic activities of daily living (Table 3), with the joint force mainly being generated through contraction of shoulder muscles including the deltoid, pectoralis, latissimus dorsi, supraspinatus and infraspinatus. Although the findings of this study cannot be validated directly, they are in agreement with instrumented implant measurements as well as predictions from other musculoskeletal shoulder models that were validated against these measurements (Bergmann et al., 2007; Nikooyan et al., 2010; Veeger et al., 2006; Westerhoff et al., 2009). The peak glenohumeral contact forces for activities such as driving slow/fast as well as abduction (fast) and flexion (fast) were measured by Bergmann et al. (2007) as ranging between 40% BW and 57% BW, while our study computes joint forces between 33% BW (SD 9%) and 58% BW (SD 15%). Charlton and Johnson (2006) estimated peak glenohumeral contact forces to range from 23% to 75% BW for 10 functional activities including feeding, personal hygiene and lifting everyday objects, with results of this study predicting a range of 26% BW (SD 7) to 69% BW (SD 32) for these functional daily activities. The small differences between data from this study (young healthy volunteers) and instrumented implant measurements (elderly participants) may be explained by kinematic differences.

The findings of this study demonstrate substantial shear force components on the glenoid plane during functional activities of daily life. The largest ratios of shear force component to compression force component are computed for activities such as reaching across the body (1.09; SD 0.41), picking and placing everyday objects (0.88; SD 0.36), pulling and pushing (0.81; SD 0.33), sitting and standing (0.5; SD 0.22) as well as cleaning the back (0.57; SD 0.27). These results suggest that glenohumeral shear force components are substantial not only when high loads act at long lever arms but also at high angles of arm elevation (Fig. 3, Fig. 4; see Supplementary material for contact forces and joint angles for the entire dataset).

The shear force components presented in this study are comparable to the study of Anglin et al. (2000) that presented shear force components of 15% to 40% BW for demanding, functional daily activities such as walking with a cane, lifting a 5 kg box with hands from the floor to shoulder height and lifting a 10 kg suitcase laterally. The direction of the contact force on the glenoid as quantified in this study corresponds to findings by Anglin et al. (2000), demonstrating loading of the antero-superior quadrant. Similarly, loading of the superior glenoid during arm elevation is consistent with Karlsson and Peterson (1992), Poppen and Walker (1978) and Van der Helm (1994). Differences between individual studies may be based kinematic differences due to large variability in subject characteristics and joint angles (see Supplementary material).

The detailed understanding of glenohumeral contact forces and loading directions during essential functional activities of daily living as provided in this study will allow preclinical test procedures for shoulder arthroplasties to be improved in order to improve implant design and fixation with off-centre loading, where this is the major cause of glenoid loosening (Geraldes et al., 2017). The existing guidelines for preclinical testing are based on the study by Anglin et al. (2000), with findings from this study allowing improvements in testing protocols based on shear force components for essential functional activities of daily life that partly exceed the range of shear force components as presented by Anglin et al. (2000).

### 4.2 Glenohumeral contact forces to aid rehabilitation planning

The precise understanding of glenohumeral compressive force components and more importantly glenohumeral shear force components during essential functional activities of daily life will aid rehabilitation planning and allow advice to be given to patients about safe activities in order to avoid joint overloading post Bankart repair, for example. The existing rehabilitation guidelines following an anterior stabilisation aim to optimise the healing of the Bankart repair and capsuloligamentous structures while controlling pain and reducing immobilisation times to reduce muscle atrophy (McDermott and Wallace, 1999, Bottoni et al., 2002, Kibler et al., 2001). Therefore, patients are advised to keep the arm in a sling for 6 weeks post-surgical intervention with exceptions being made for basic tasks such as feeding and personal hygiene (Dines and Levinson (1995). The data presented in this study demonstrate that the ratios of glenohumeral anterior shear force component to compression force component are low, ranging between 0.09 (SD 0.04) and 0.14 (SD 0.09), while the inferior glenohumeral ratios range from 0.08 (SD 0.02) to 0.13 (SD 0.03). Consequently, the findings of this study provide scientific support for current rehabilitation guidelines regarding feeding tasks; this scientific evidence gives credence to clinical advice given, with the potential to reduce patients' fear and anxiety relating to re-injury and thereby improving confidence in performing activities of daily living.

In contrast to feeding tasks, the glenohumeral ratios for functional activities of daily life such as perineal care, cleaning back and reaching opposite axilla range between 0.16 (SD 0.07) and 0.25 (SD 0.11) and between 0.17 (SD 0.09) and 0.58 (SD 0.36) for anterior and superior ratios, respectively. These data suggest that tasks of personal hygiene put a higher demand on a Bankart repair. In fact, clinically, post shoulder stabilisation procedures patients report difficulty reaching to the opposite axilla to clean with the operated arm (Dawson et al., 1996). The data in this study may help to explain why. The inferior and anterior shear force components experienced during this movement have the potential to produce patient “apprehension” via proprioceptive feedback mechanisms from the joint and soft tissues (Dean et al., 2017; 2013). This may, in turn, produce protective muscle activity reducing range of motion in the more cautious patient post repair (Ginn and Cohen, 2005).

The current rehabilitation guidelines following an anterior stabilisation recommend avoiding using arm rests while standing up or sitting down on a chair in order to avoid overloading the labral repair within 12 weeks post-surgical intervention (Kibler et al., 2001; Murphy et al., 2013). The data presented in this study provide scientific support for those guidelines as the anterior glenohumeral ratio during the sit-to-stand task amounts to 0.50 (SD 0.22), with large shear force components impairing the recovery process due to high stresses exerted onto the Bankart repair.

The clinical guidelines for rehabilitation post Bankart repair also recommend not driving (and, therefore, not steering a car) within 12 weeks post-surgery for the same loading related reasons. The findings of this study demonstrate that the glenohumeral ratios are much less significant for the driving activity, ranging between 0.02 (SD 0.01) and 0.09 (SD 0.04) as well as 0.19 (SD 0.05) and 0.23 (0.08) for superior and anterior ratios. In fact, the shear force components during the driving task are amongst the lowest of all 26 functional activities of daily living. Therefore, the data presented in this study suggest that the driving task is much less demanding for an anterior stabilisation from a mechanical point of view than previously assumed. We acknowledge that the repetitive nature of the driving task might increase the demand on the Bankart repair and reduce the load required for overloading the repaired structure (Uhl et al., 2010). However, the effect of repetitive motions on the load of the Bankart repair is challenging to evaluate and the peak shear force components were obtained at large rotation angles of the steering wheel of 60°. Therefore, the results of this study provide an indication that the driving task is less demanding for a Bankart repair than recommended in rehabilitation guidelines.

### 4.3 Limitations of this study

This study has several modelling limitations. First of all, the scapula kinematics for the ADL1 dataset was derived from regression equations rather than measured kinematics. However, given the moderate joint angles for a large number of those activities, the effect should be relatively small (Pandis et al., 2015). Secondly, the validation of musculoskeletal shoulder models has demonstrated small differences between model output and instrumented shoulder implant measurements. The model improvement could include patient-specific scaling of the model and optimisation of the muscle load-sharing cost function. Thirdly, not all functional activities of daily living were performed by the same participants. Fourthly, the glenohumeral compressive force component is directed towards the mid-glenoid as the UKNSM does not account for humeral head translation. As the humeral head exhibits a small degree of in-vivo translation during functional daily activities (Nishinaka et al., 2008), the loading is not applied through the centre of the glenoid, which produces a torque around the glenoid-labral socket. As the articulating surface of the glenoid is rather shallow and translational movements are small (Howell and Galinat, 1989), this effect is expected to be small. Finally, the presented shoulder loads can only be partly transferred between different age groups due to kinematic changes (Nikooyan et al., 2010), and the presented data are only valid in the glenoid coordinate system and will have to be transformed into the humeral frame when assessing humeral loading during functional activities of daily living.

## 5 Conclusion

This study analyses shoulder compression force components and shear force components during 26 functional activities of daily life utilizing a musculoskeletal shoulder model. The results demonstrate substantial loads through the shoulder with the contact force exceeding 50% of the body weight in 10/26 activities of daily living. The ratio of glenohumeral shear force component to compression force component exceeds 0.5 in 8/26 functional activities, with glenohumeral ratios for tasks involving for reaching across the body (1.09; SD 0.41) and picking and placing an everyday object (0.88; SD 0.36). The loading of the joint is considerable not only when high loads act at long lever arms but also at high angles of arm elevation. This improved understanding of glenohumeral joint loading will aid implant design, design of surgical procedures and rehabilitation planning.

## Appendix A Supplementary data

Supplementary material 1 Image 1

Supplementary material 2 Image 2
