# Supplementary material for: Analysis of shoulder compressive and shear forces during functional activities of daily life
Source: Clin Biomech (Bristol). 2018 May;54:34–41. doi: 10.1016/j.clinbiomech.2018.03.006 (PMC6405441; doi:10.1016/j.clinbiomech.2018.03.006)
Supplement: Supplementary file 1 — Supplementary material 1 [file mmc1.pptx]

## Slide 1
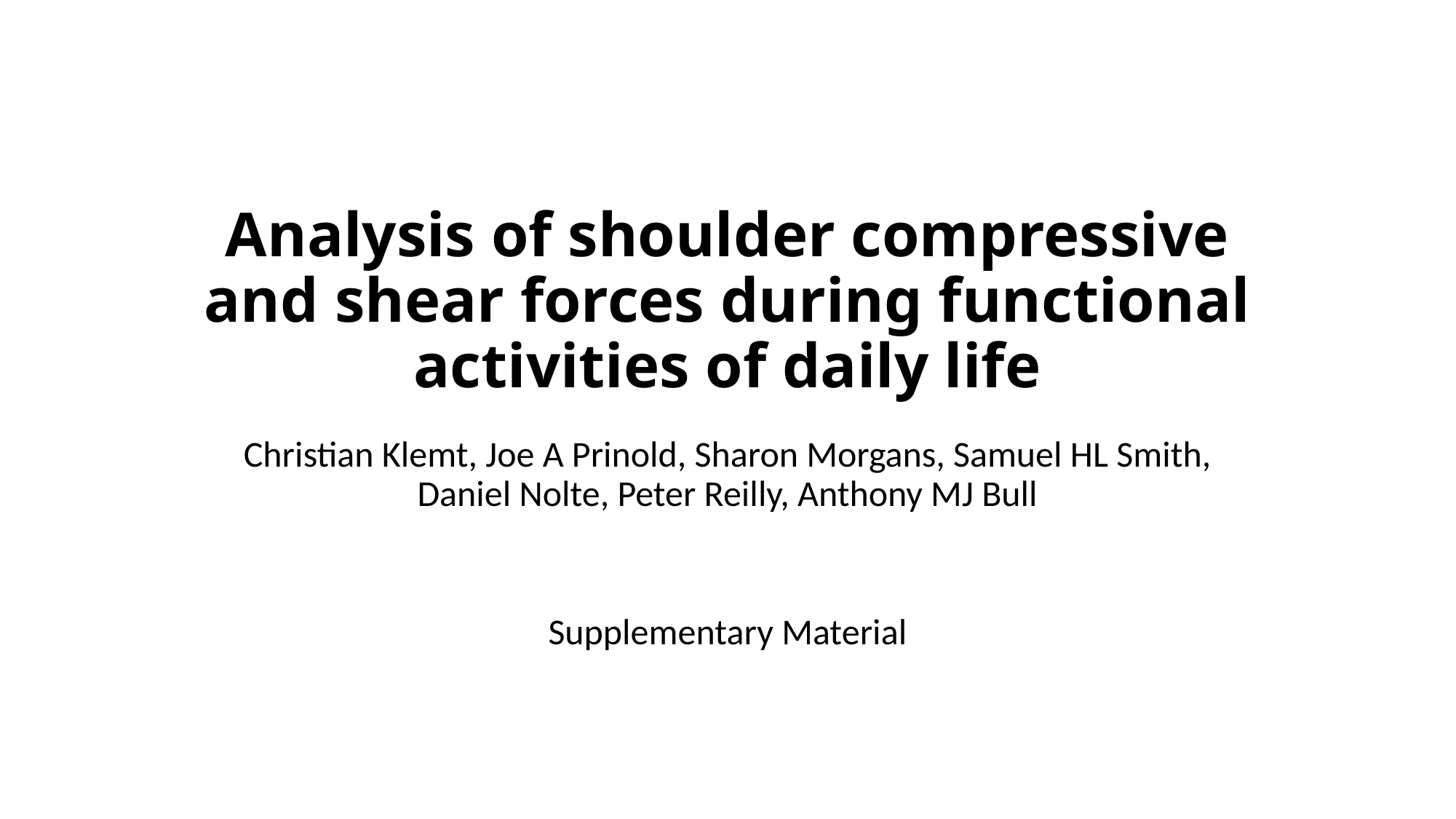

# Analysis of shoulder compressive and shear forces during functional activities of daily life
Christian Klemt, Joe A Prinold, Sharon Morgans, Samuel HL Smith, Daniel Nolte, Peter Reilly, Anthony MJ Bull
Supplementary Material

## Slide 2
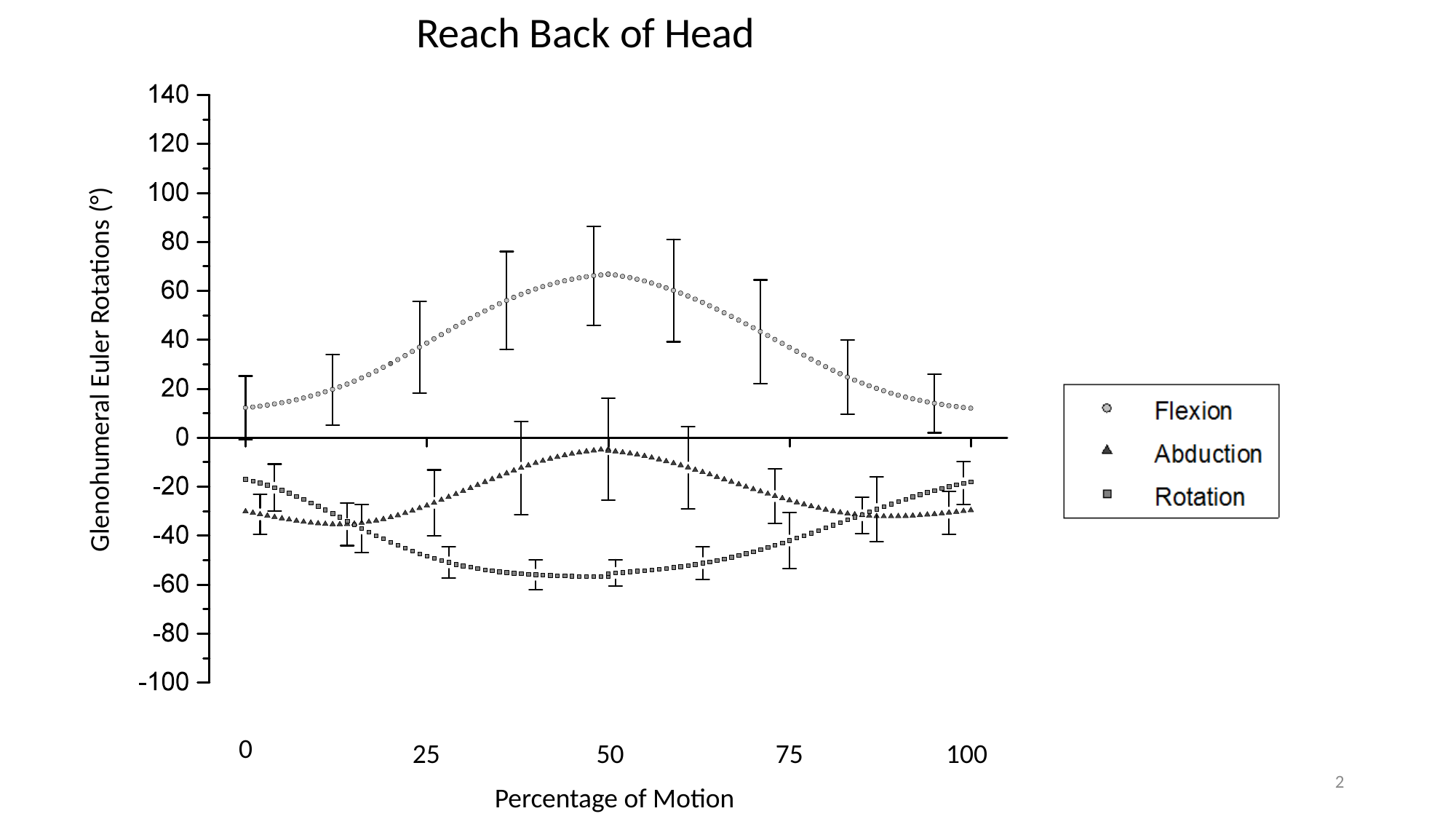

Reach Back of Head
Glenohumeral Euler Rotations (°)
0
25
50
75
100
2
Percentage of Motion

## Slide 3
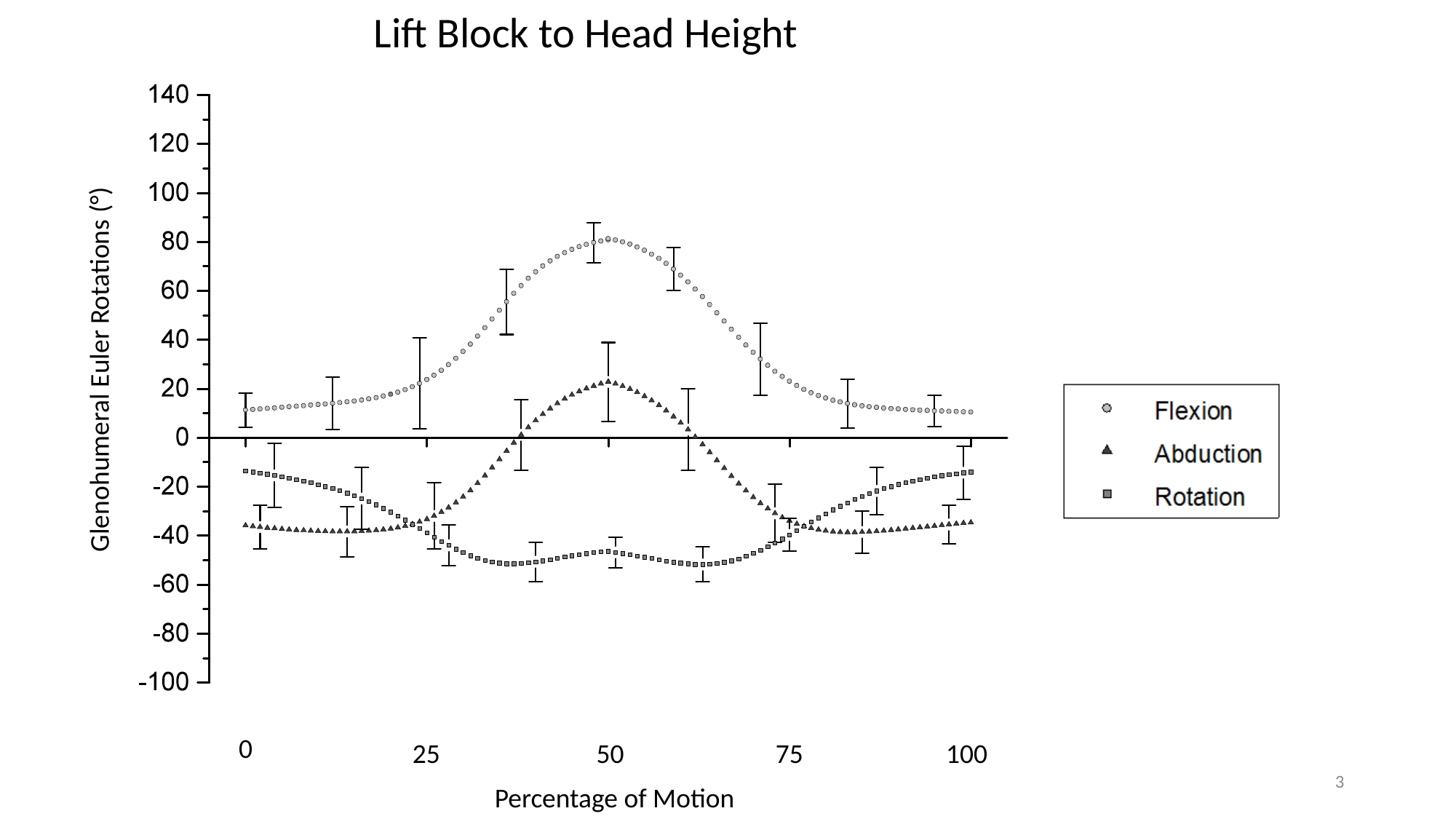

Lift Block to Head Height
Glenohumeral Euler Rotations (°)
0
25
50
75
100
3
Percentage of Motion

## Slide 4
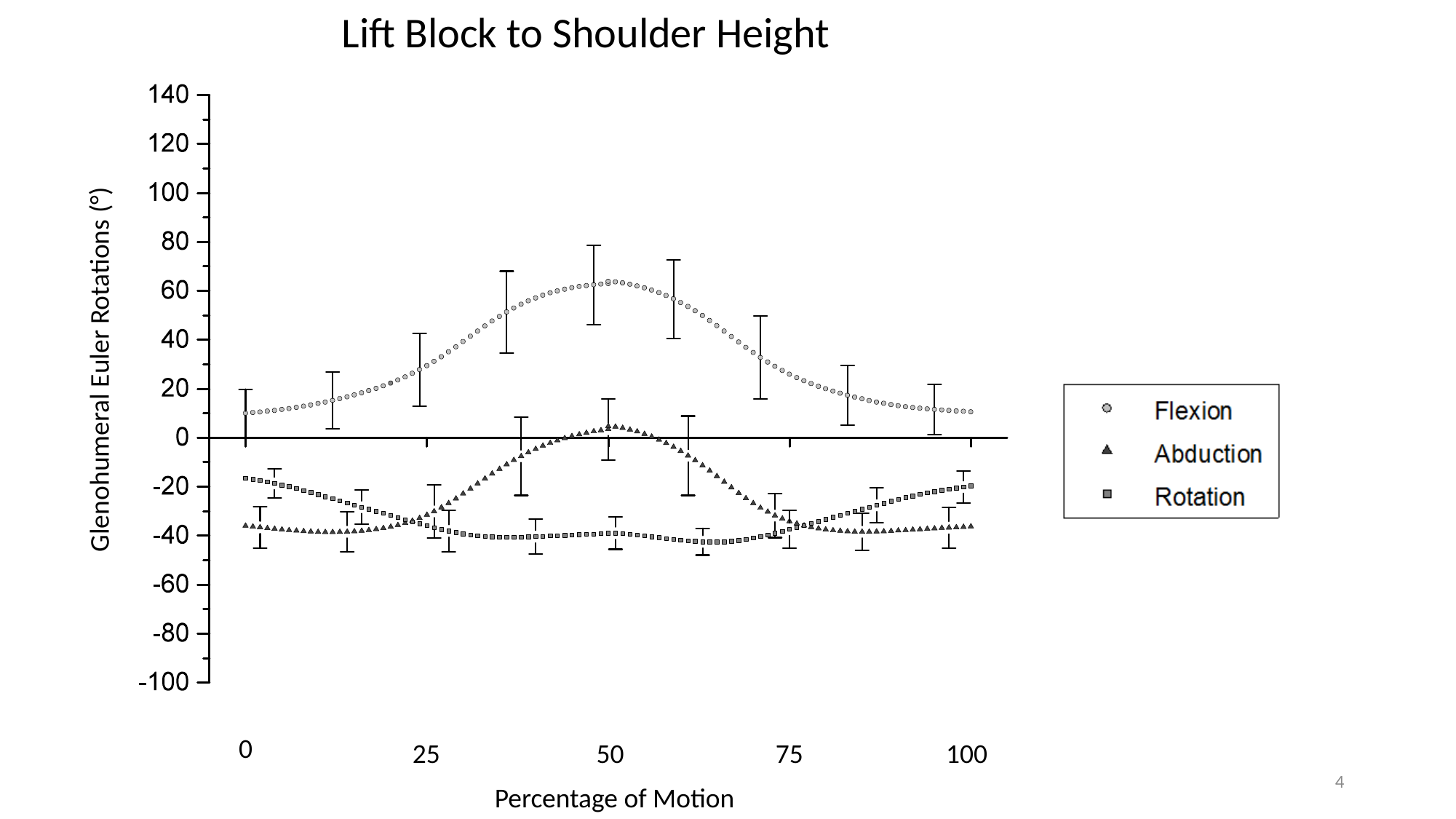

Lift Block to Shoulder Height
Glenohumeral Euler Rotations (°)
0
25
50
75
100
4
Percentage of Motion

## Slide 5
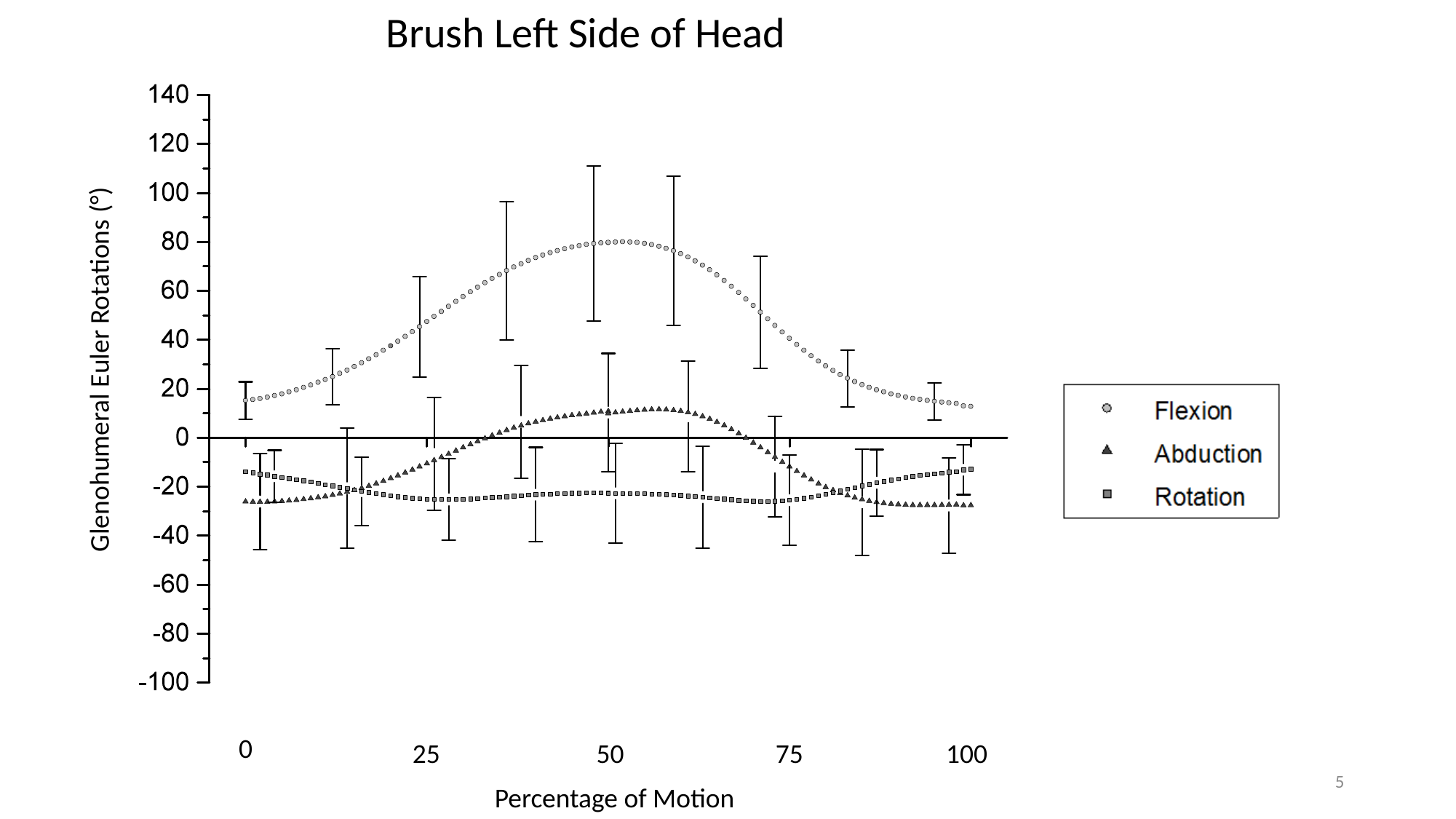

Brush Left Side of Head
Glenohumeral Euler Rotations (°)
0
25
50
75
100
5
Percentage of Motion

## Slide 6
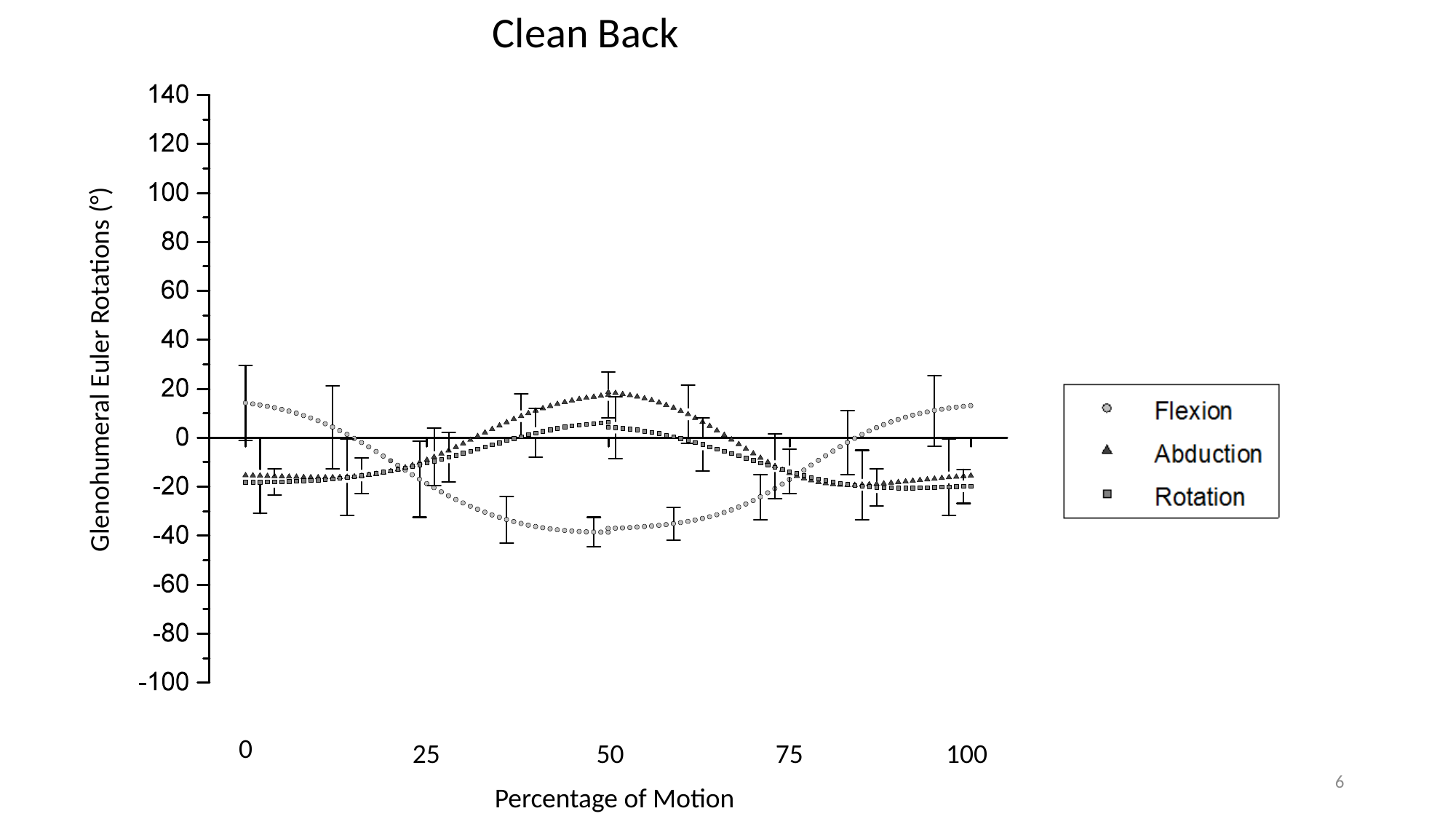

Clean Back
Glenohumeral Euler Rotations (°)
0
25
50
75
100
6
Percentage of Motion

## Slide 7
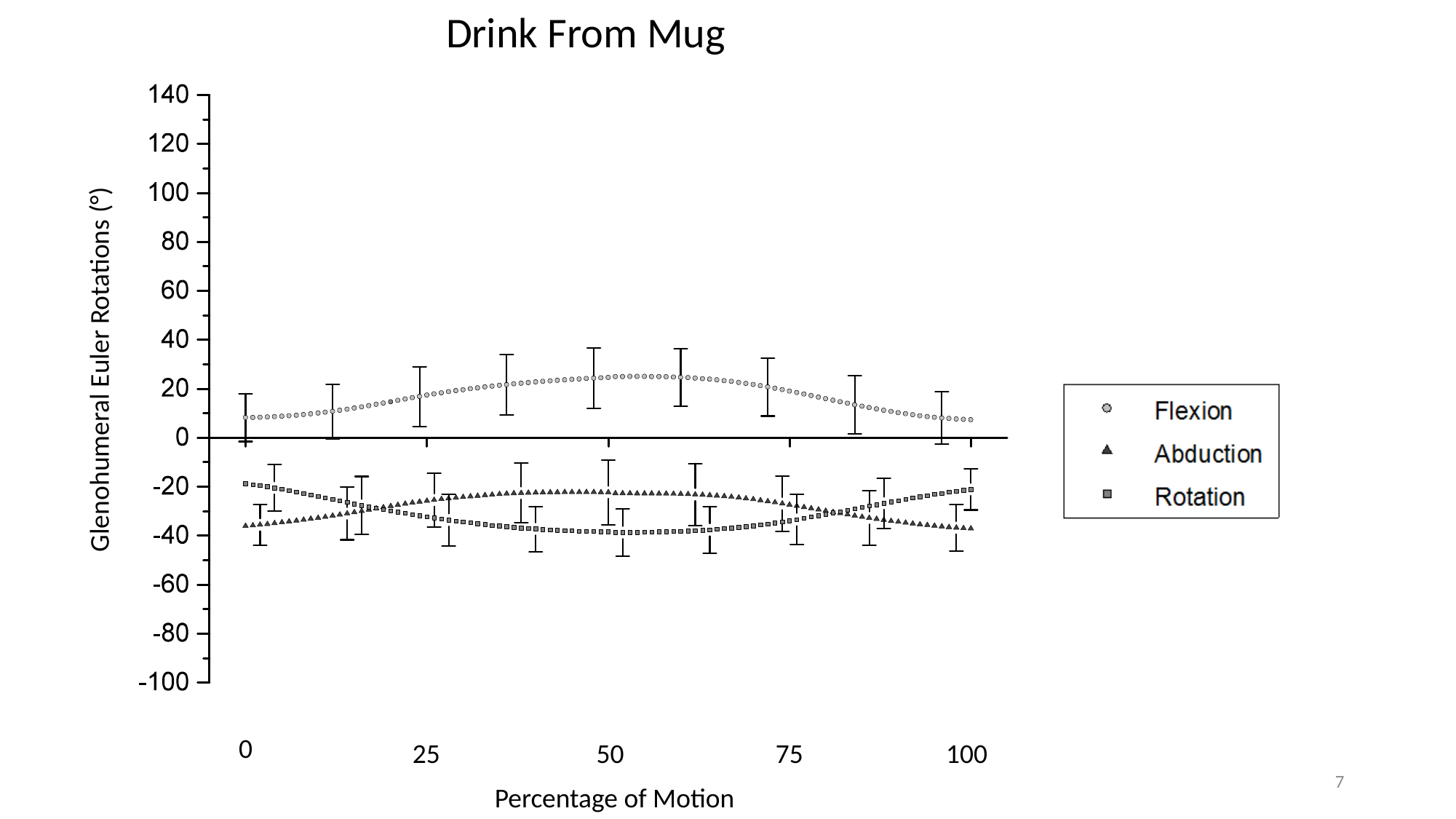

Drink From Mug
Glenohumeral Euler Rotations (°)
0
25
50
75
100
7
Percentage of Motion

## Slide 8
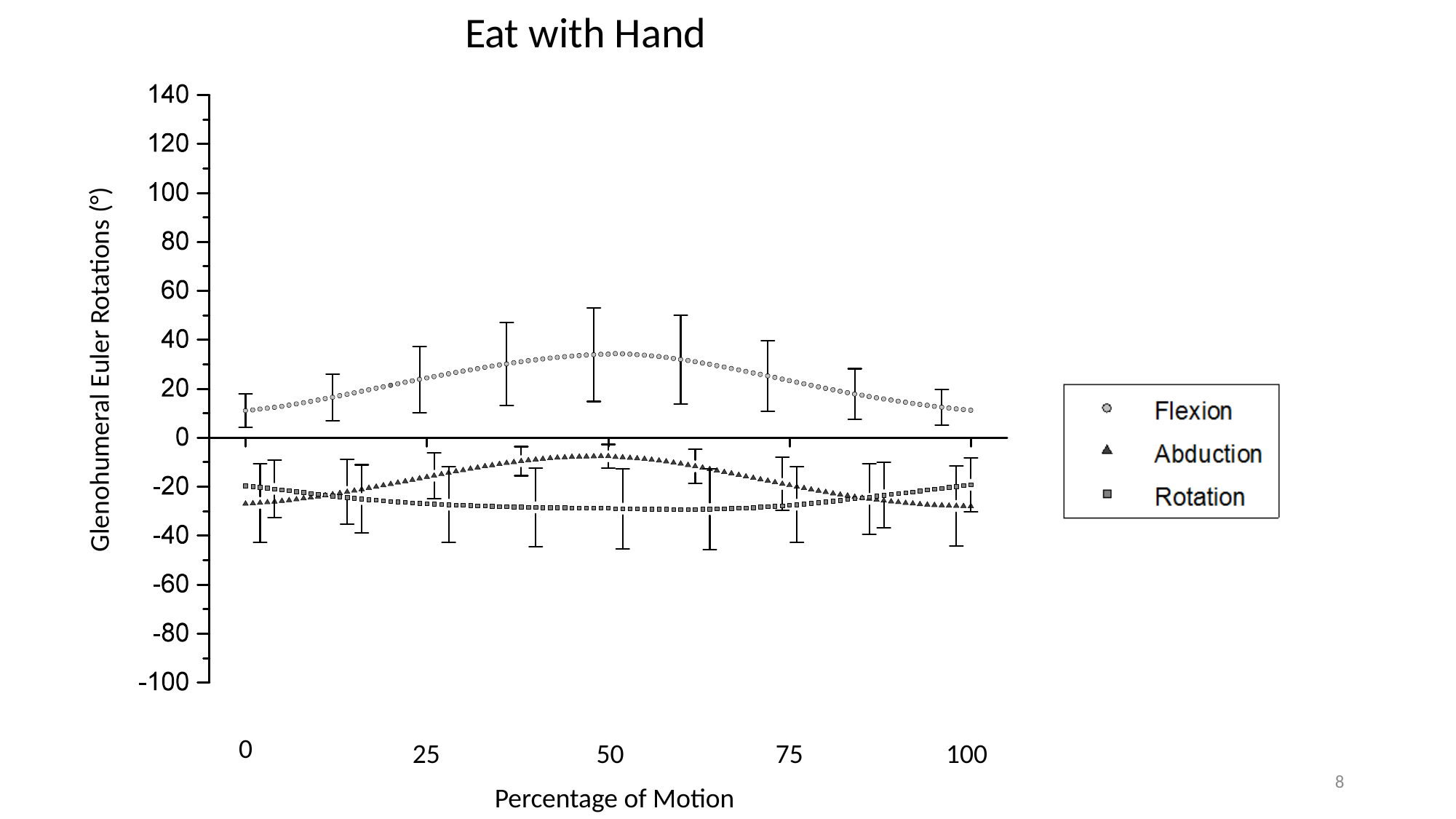

Eat with Hand
Glenohumeral Euler Rotations (°)
0
25
50
75
100
8
Percentage of Motion

## Slide 9
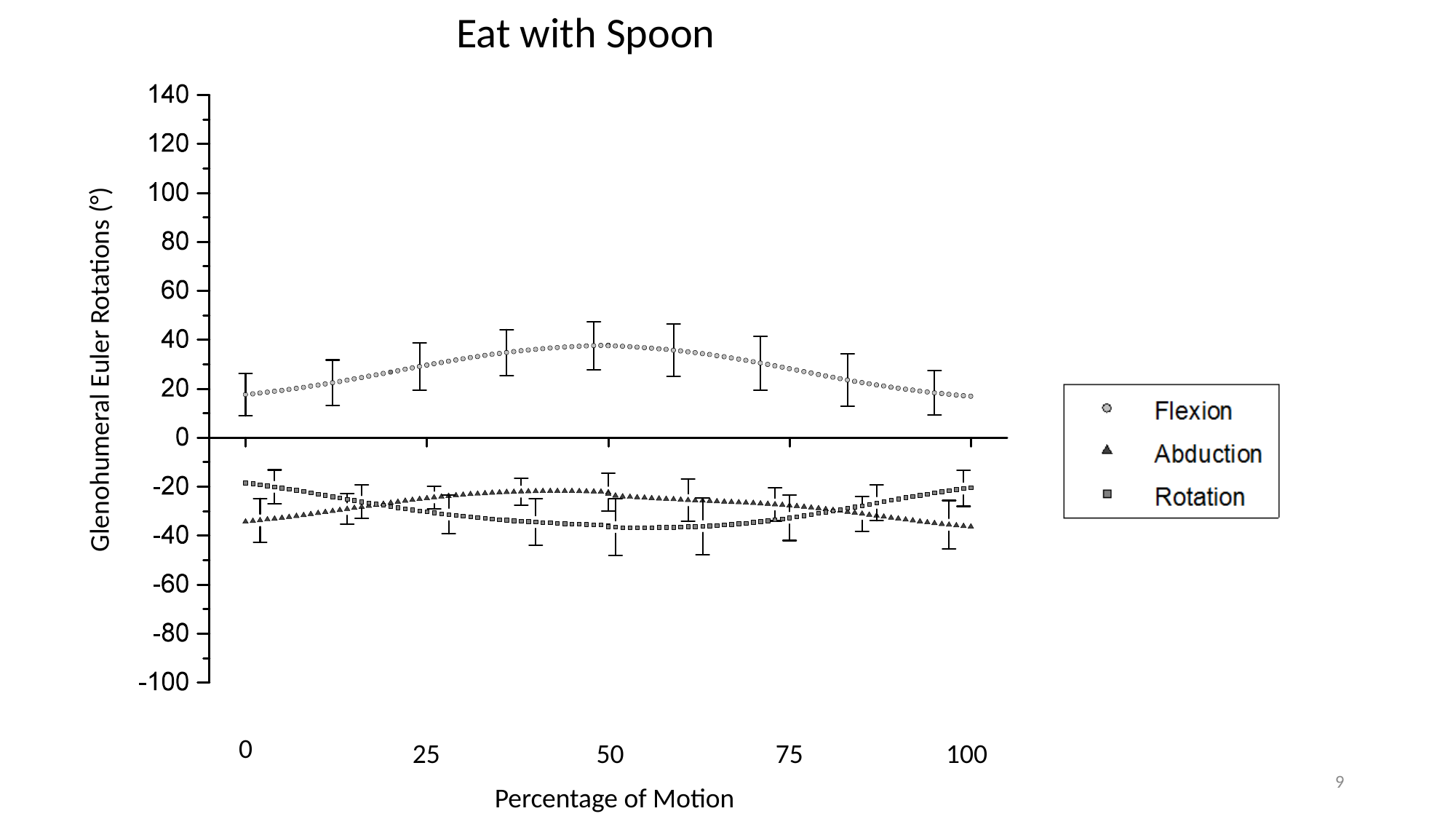

Eat with Spoon
Glenohumeral Euler Rotations (°)
0
25
50
75
100
9
Percentage of Motion

## Slide 10
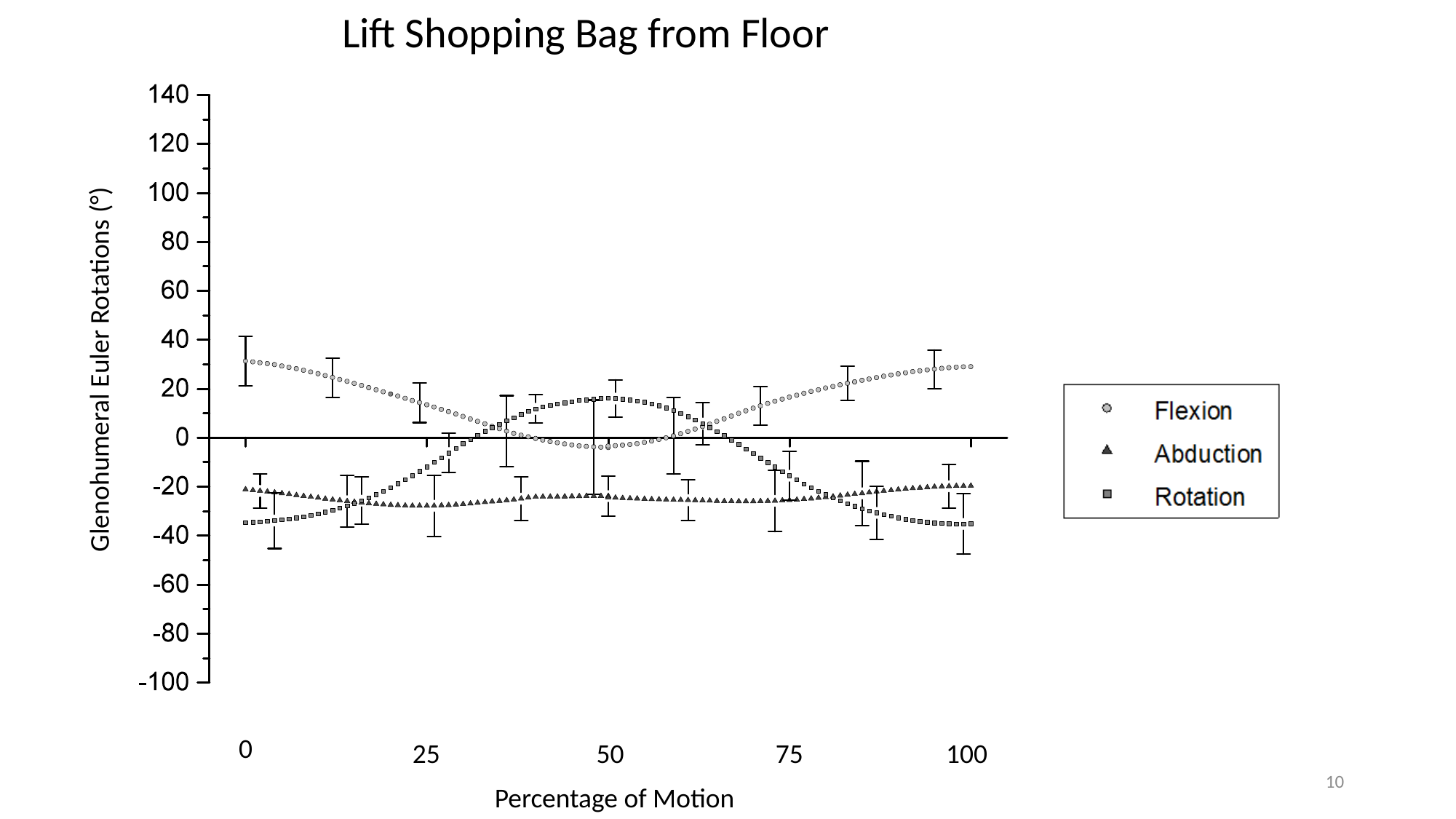

Lift Shopping Bag from Floor
Glenohumeral Euler Rotations (°)
0
25
50
75
100
10
Percentage of Motion

## Slide 11
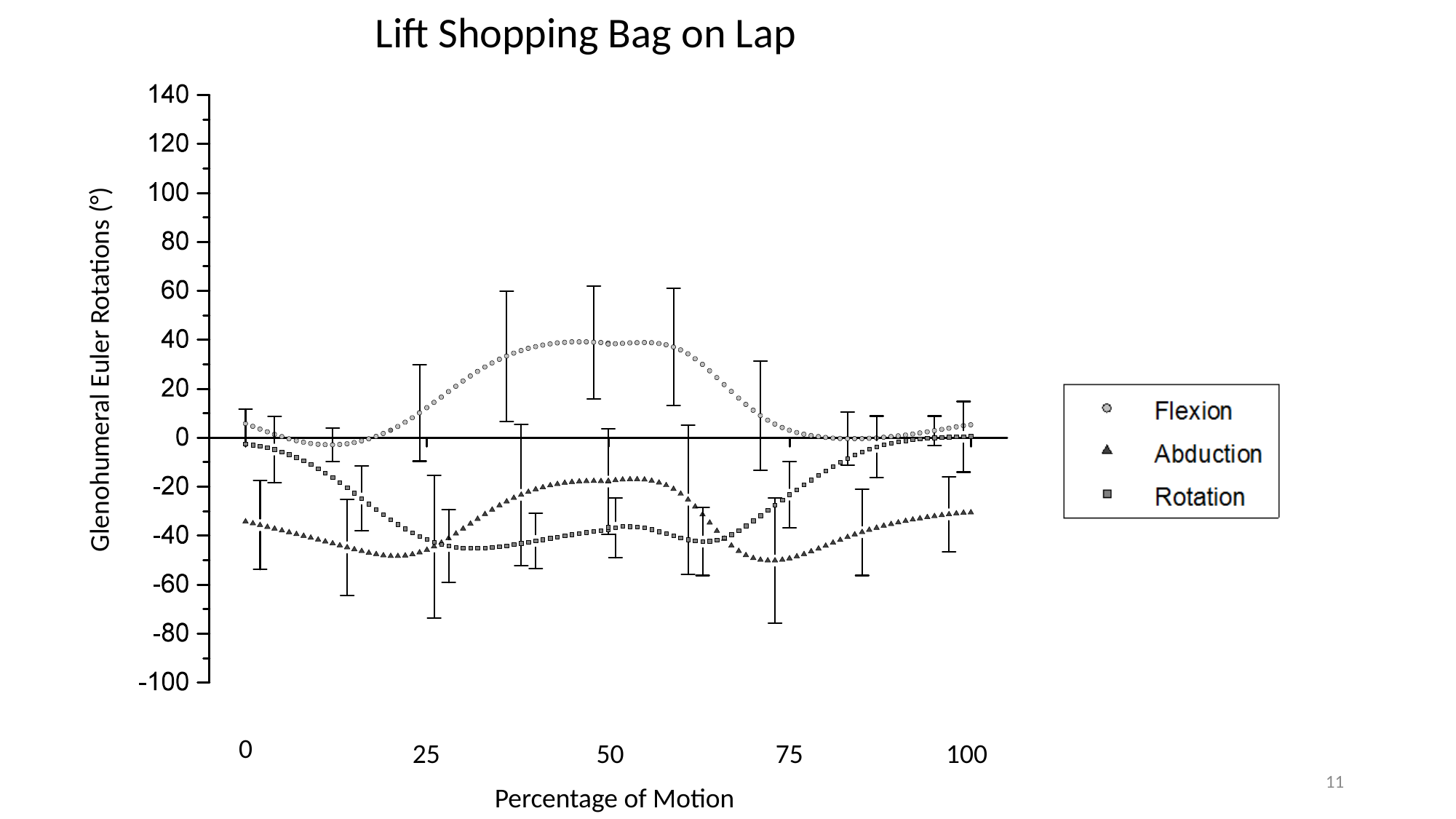

Lift Shopping Bag on Lap
Glenohumeral Euler Rotations (°)
0
25
50
75
100
11
Percentage of Motion

## Slide 12
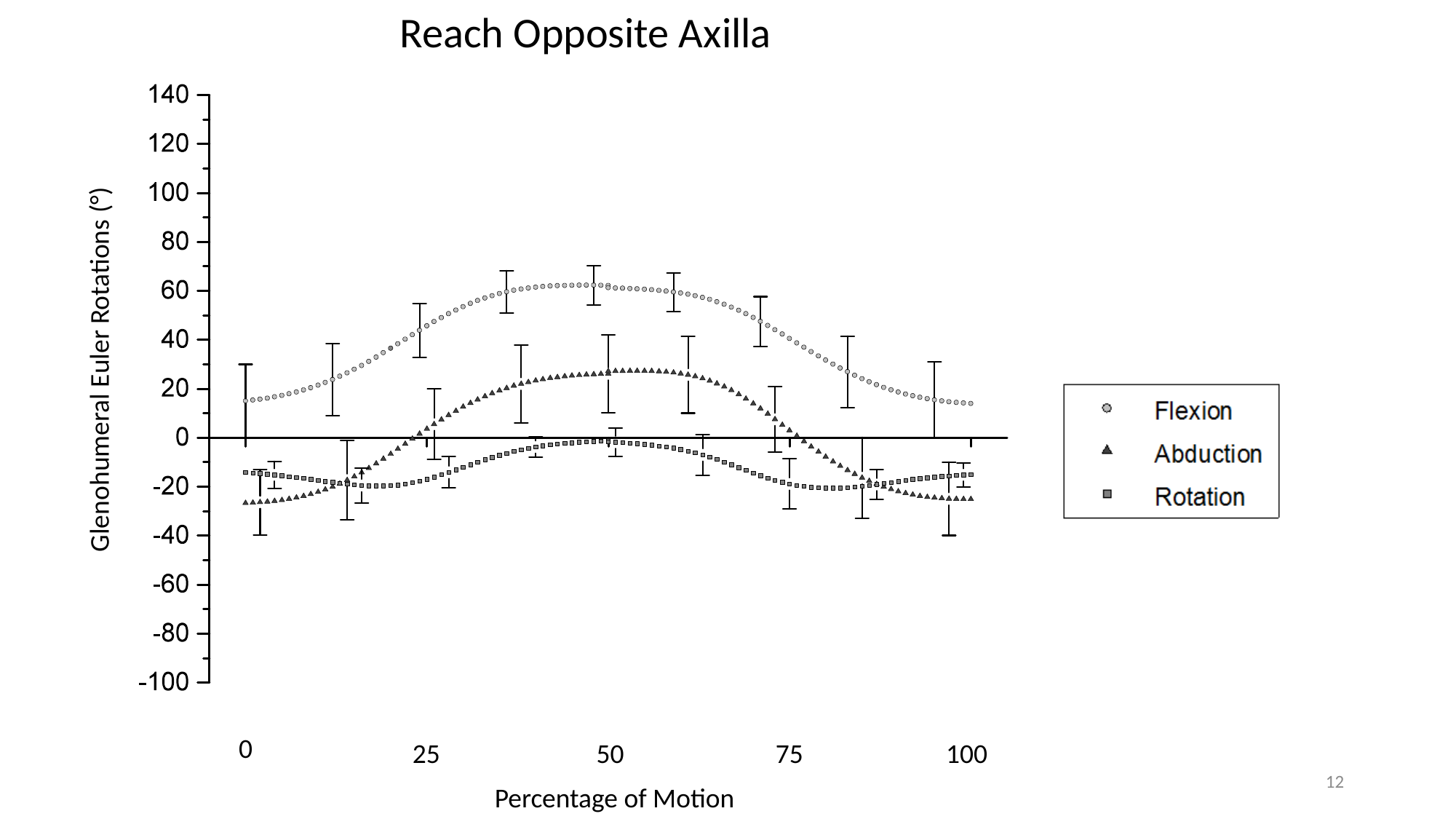

Reach Opposite Axilla
Glenohumeral Euler Rotations (°)
0
25
50
75
100
12
Percentage of Motion

## Slide 13
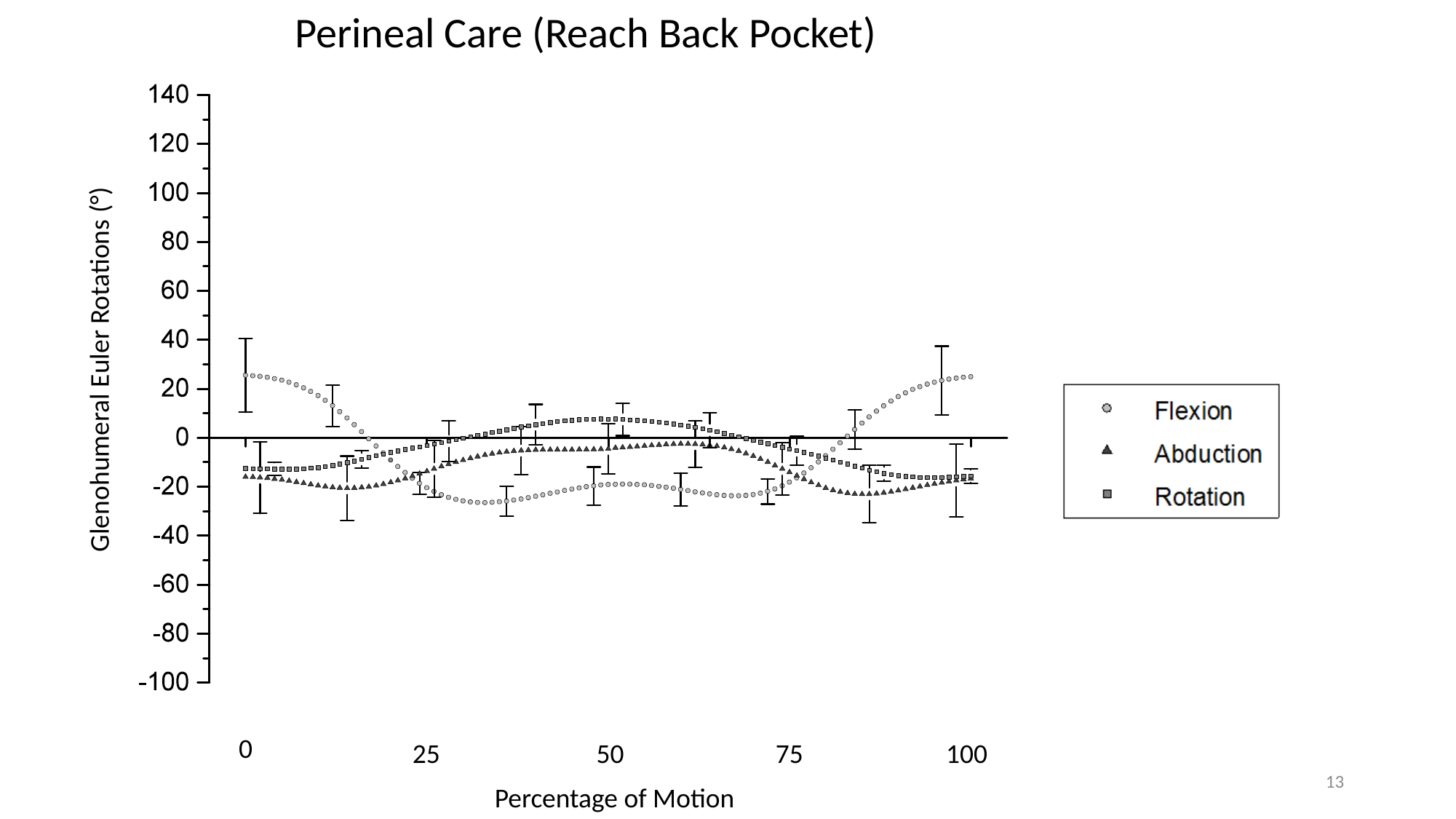

Perineal Care (Reach Back Pocket)
Glenohumeral Euler Rotations (°)
0
25
50
75
100
13
Percentage of Motion

## Slide 14
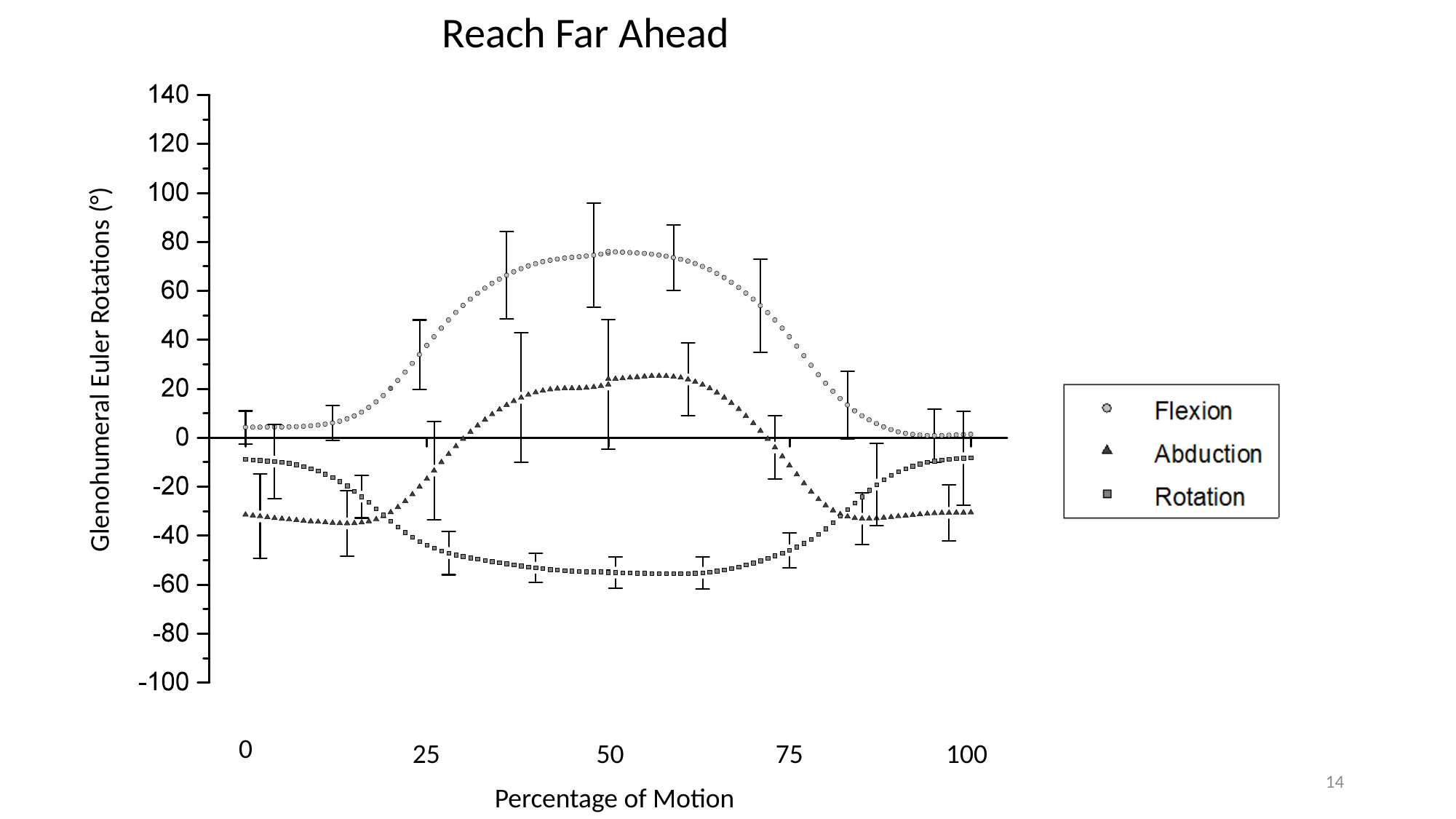

Reach Far Ahead
Glenohumeral Euler Rotations (°)
0
25
50
75
100
14
Percentage of Motion

## Slide 15
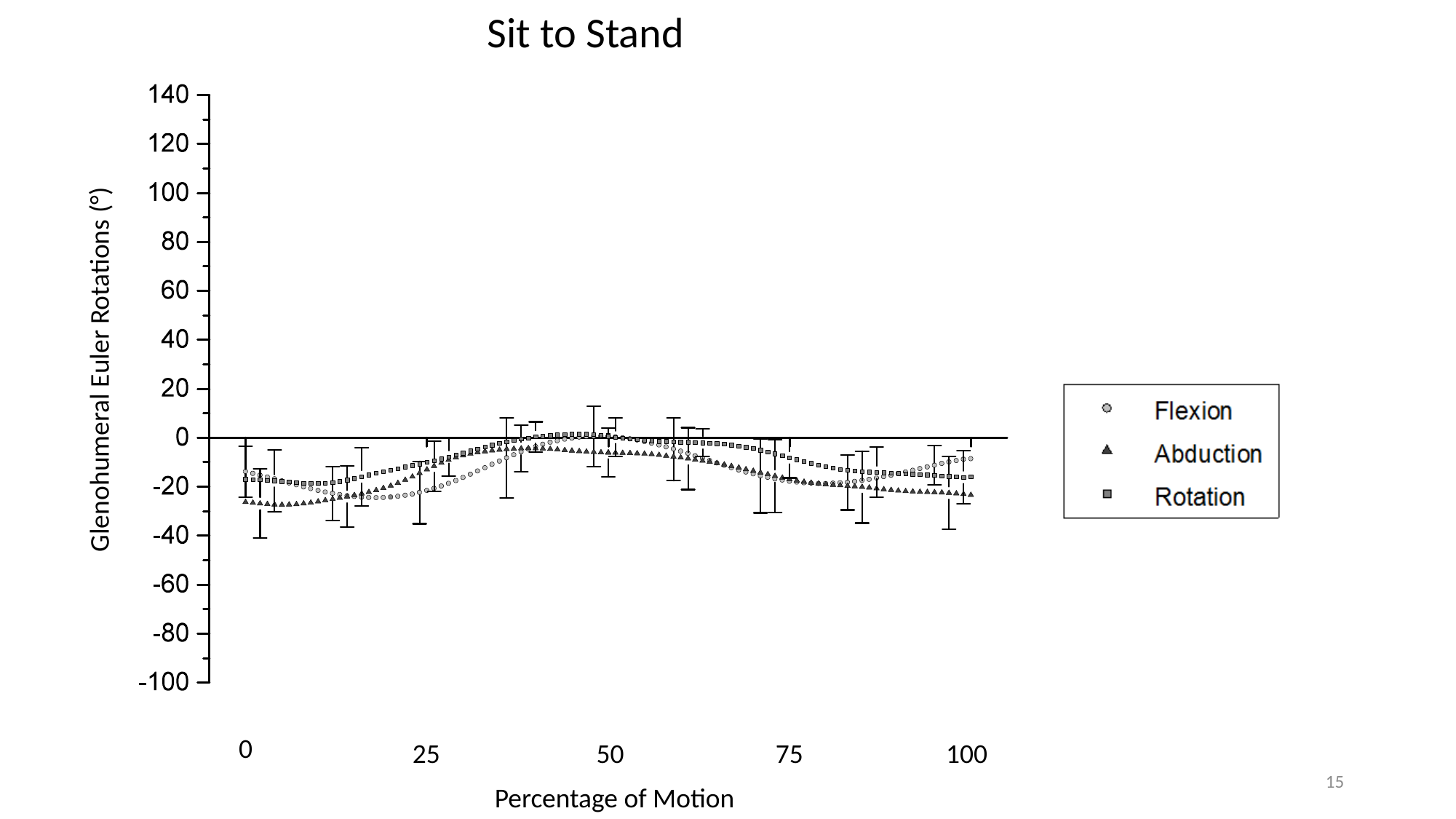

Sit to Stand
Glenohumeral Euler Rotations (°)
0
25
50
75
100
15
Percentage of Motion

## Slide 16
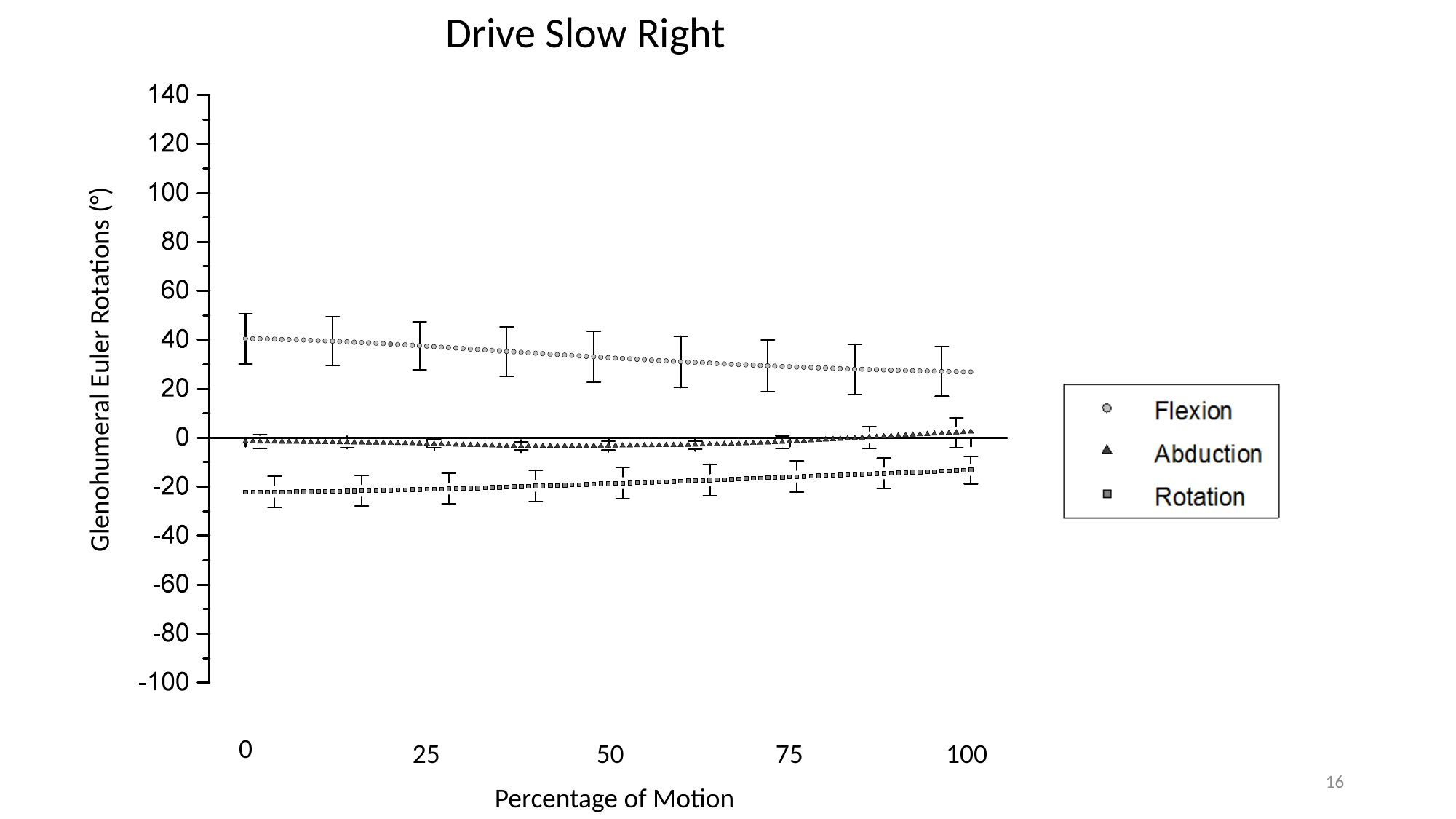

Drive Slow Right
Glenohumeral Euler Rotations (°)
0
25
50
75
100
16
Percentage of Motion

## Slide 17
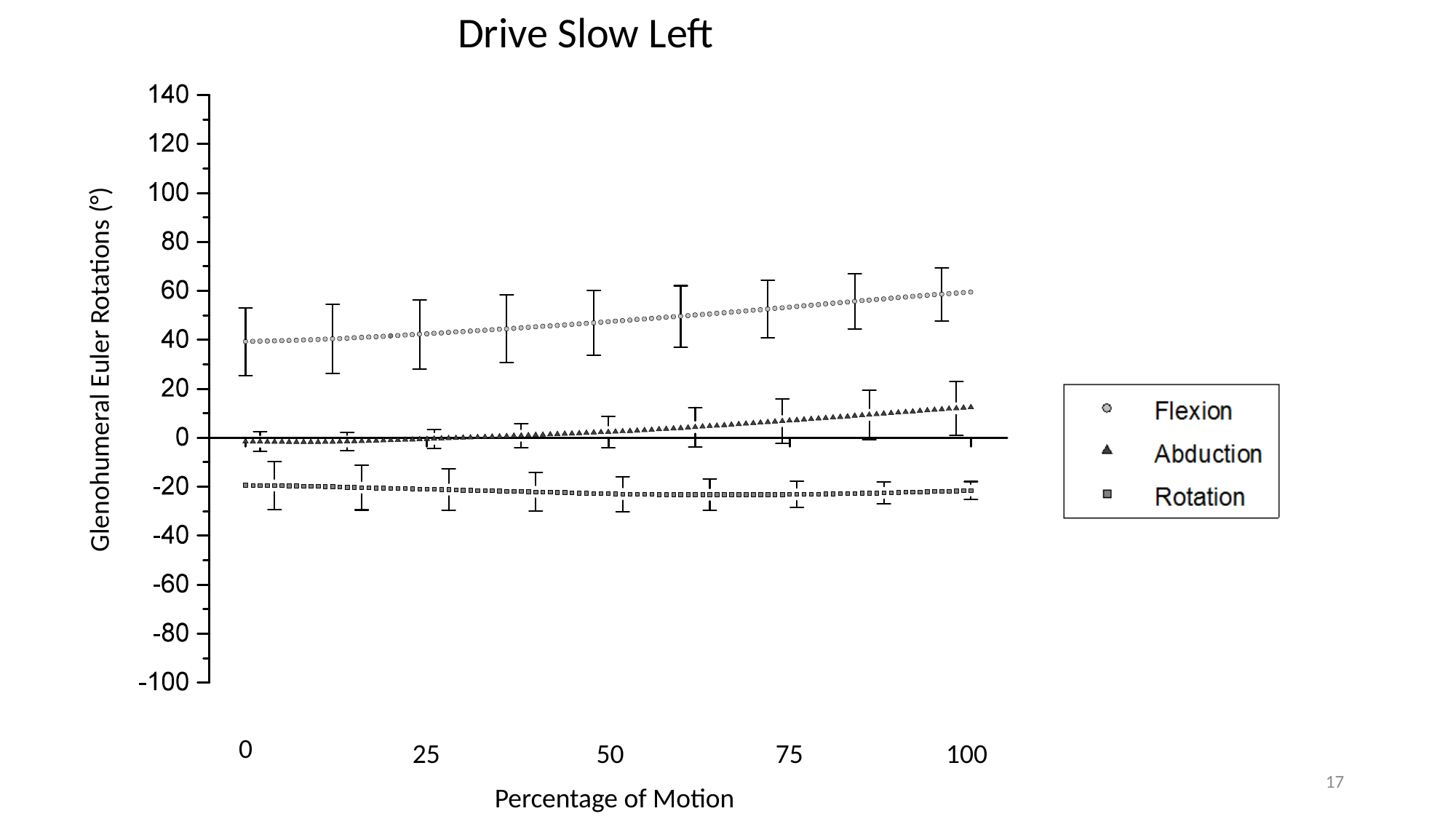

Drive Slow Left
Glenohumeral Euler Rotations (°)
0
25
50
75
100
17
Percentage of Motion

## Slide 18
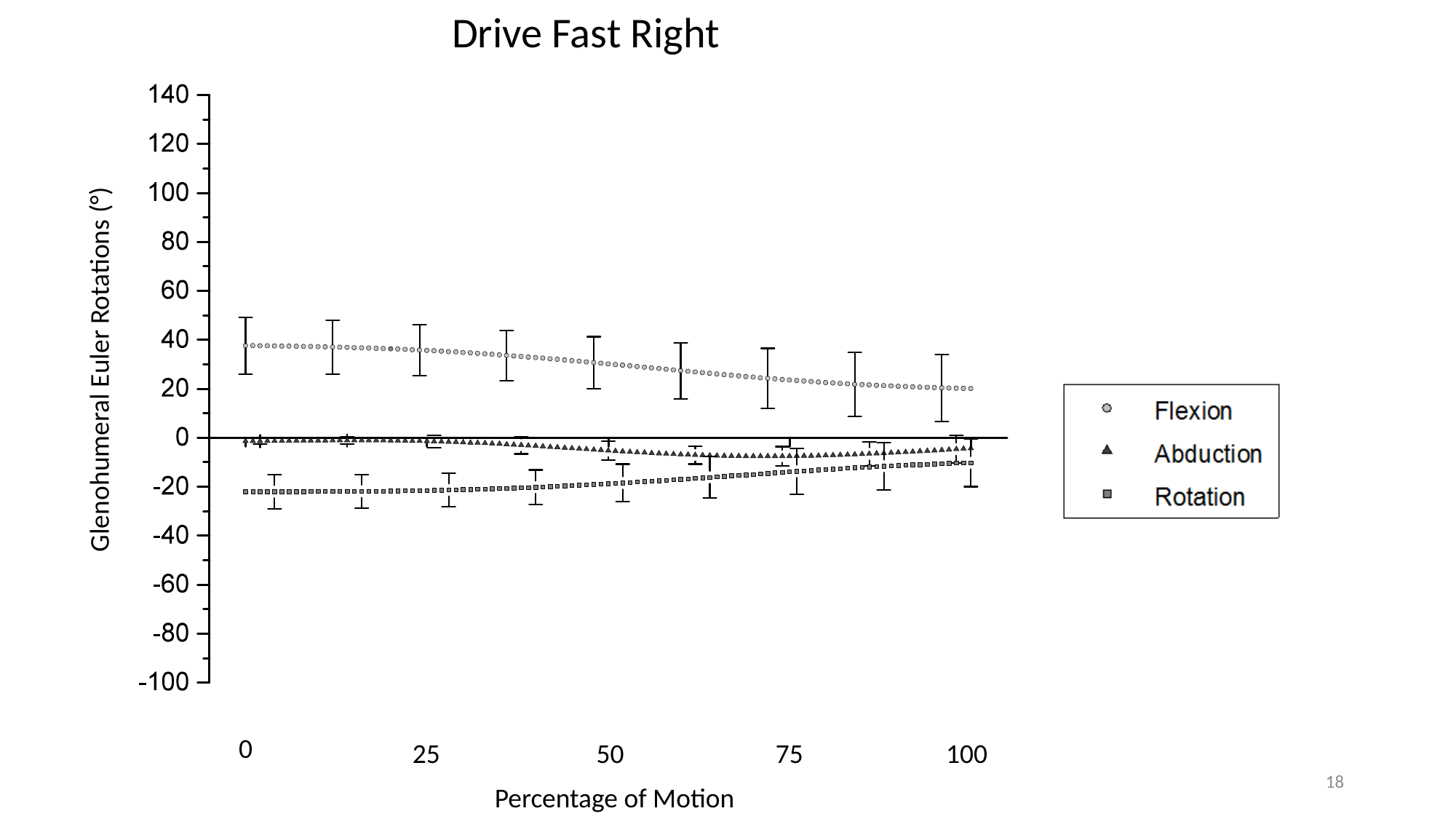

Drive Fast Right
Glenohumeral Euler Rotations (°)
0
25
50
75
100
18
Percentage of Motion

## Slide 19
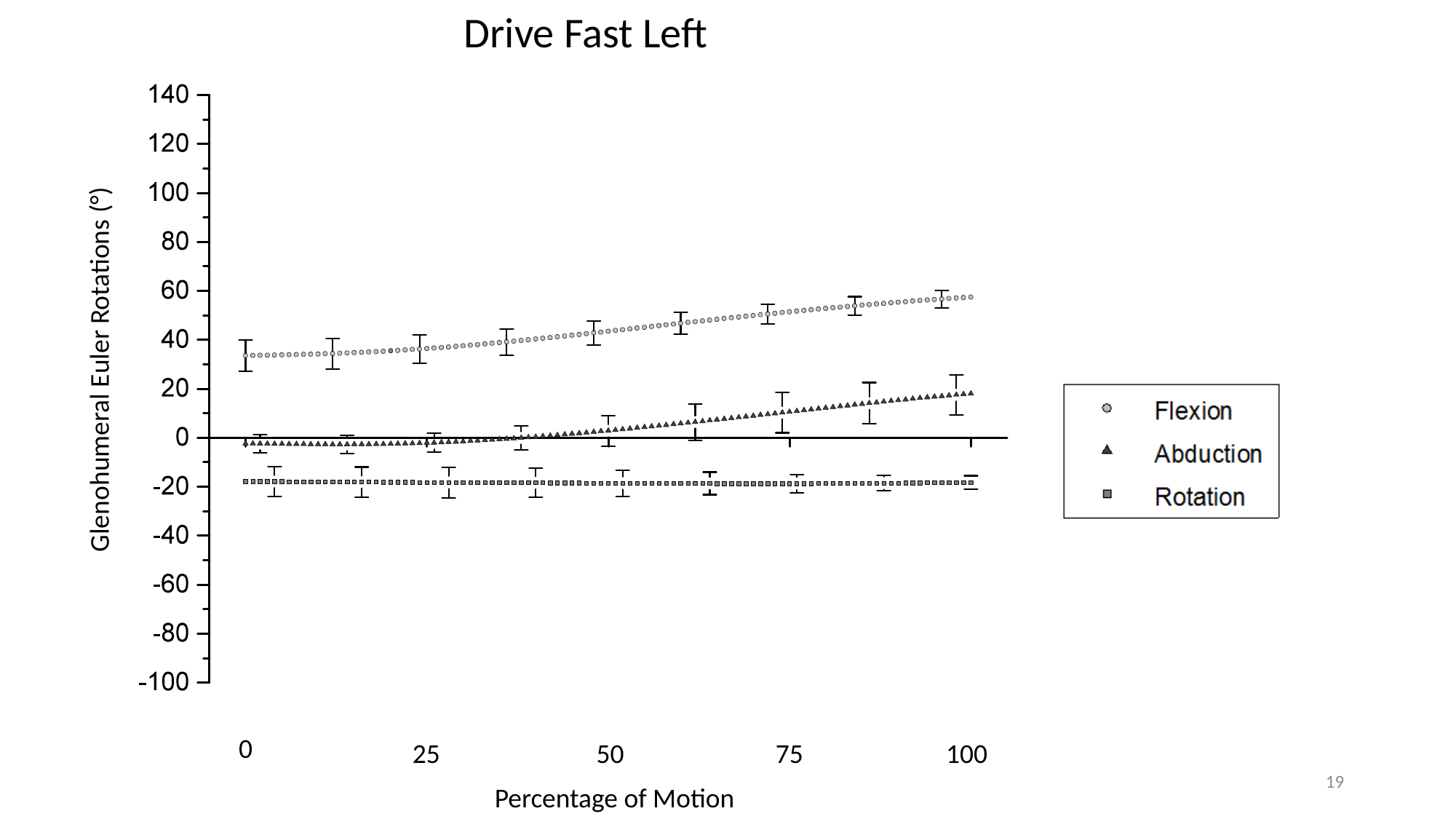

Drive Fast Left
Glenohumeral Euler Rotations (°)
0
25
50
75
100
19
Percentage of Motion

## Slide 20
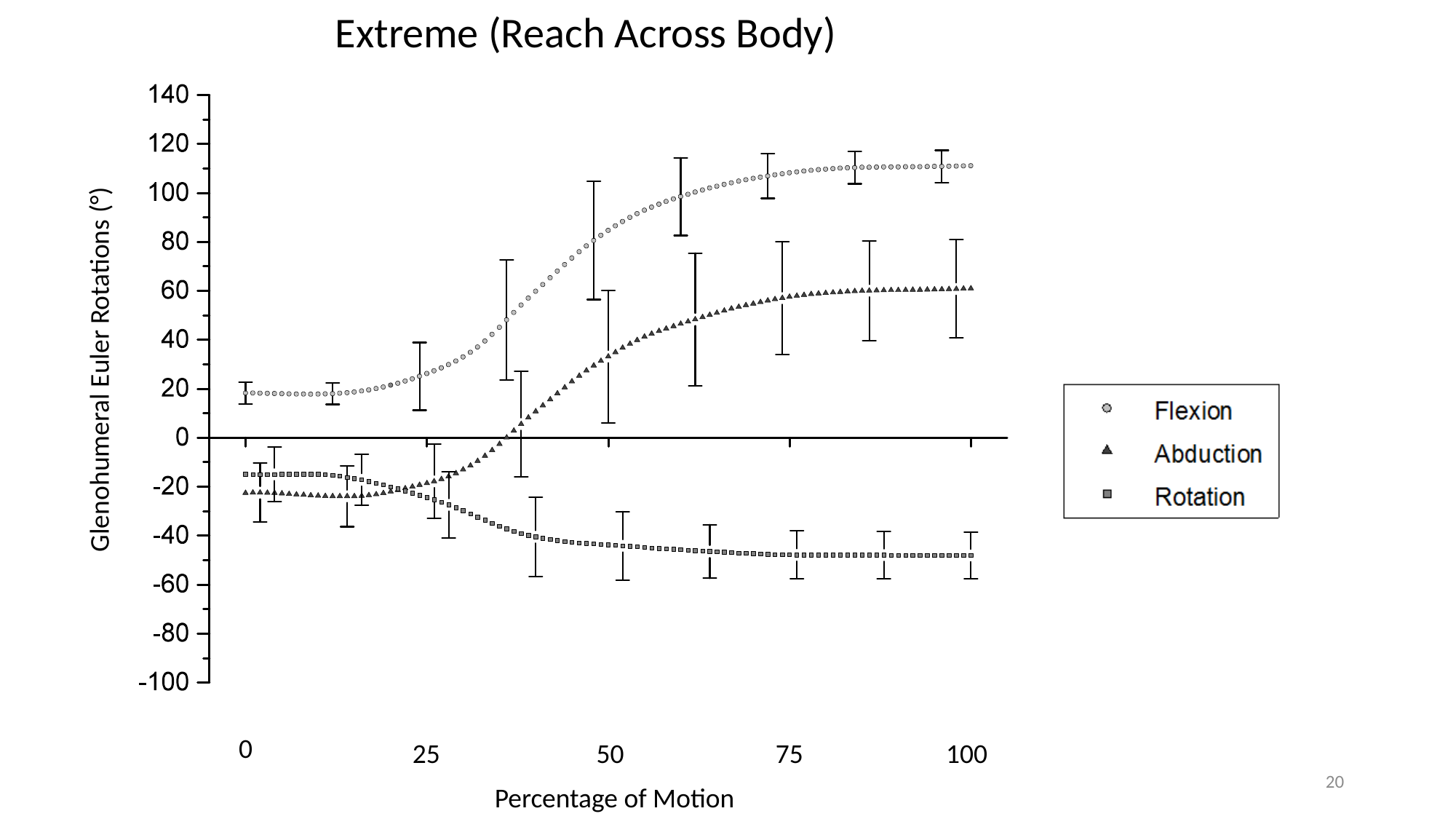

Extreme (Reach Across Body)
Glenohumeral Euler Rotations (°)
0
25
50
75
100
20
Percentage of Motion

## Slide 21
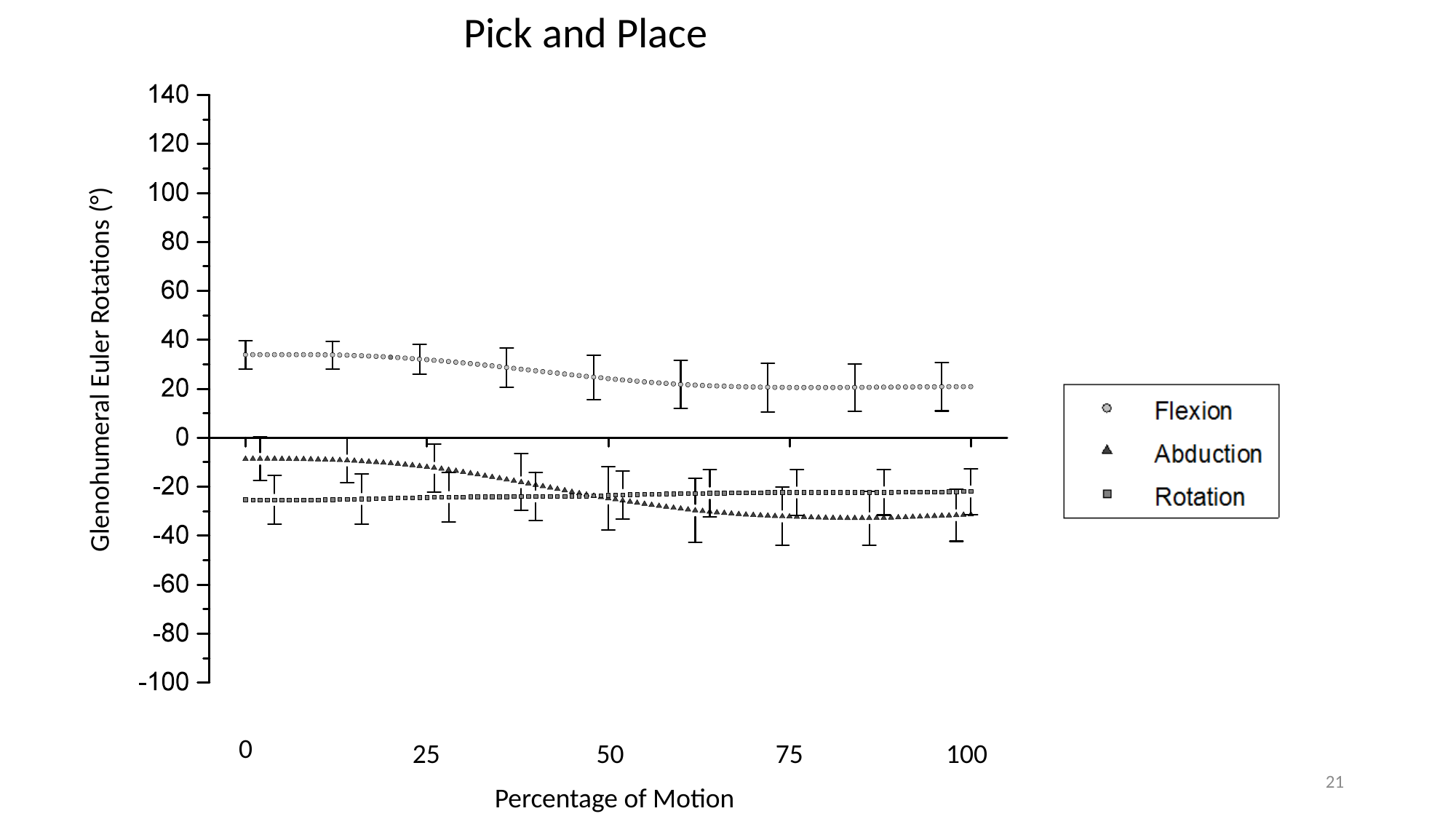

Pick and Place
Glenohumeral Euler Rotations (°)
0
25
50
75
100
21
Percentage of Motion

## Slide 22
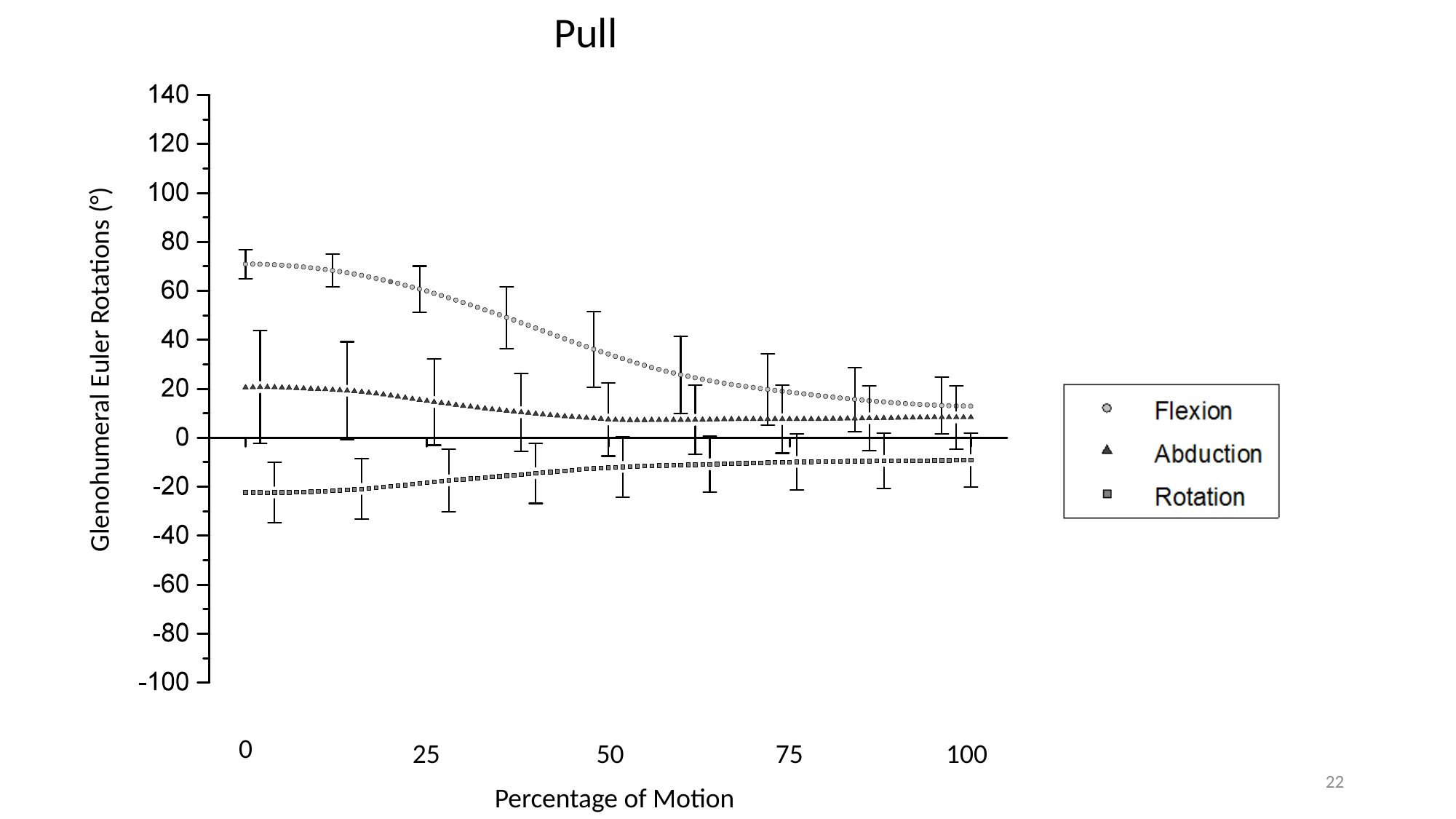

Pull
Glenohumeral Euler Rotations (°)
0
25
50
75
100
22
Percentage of Motion

## Slide 23
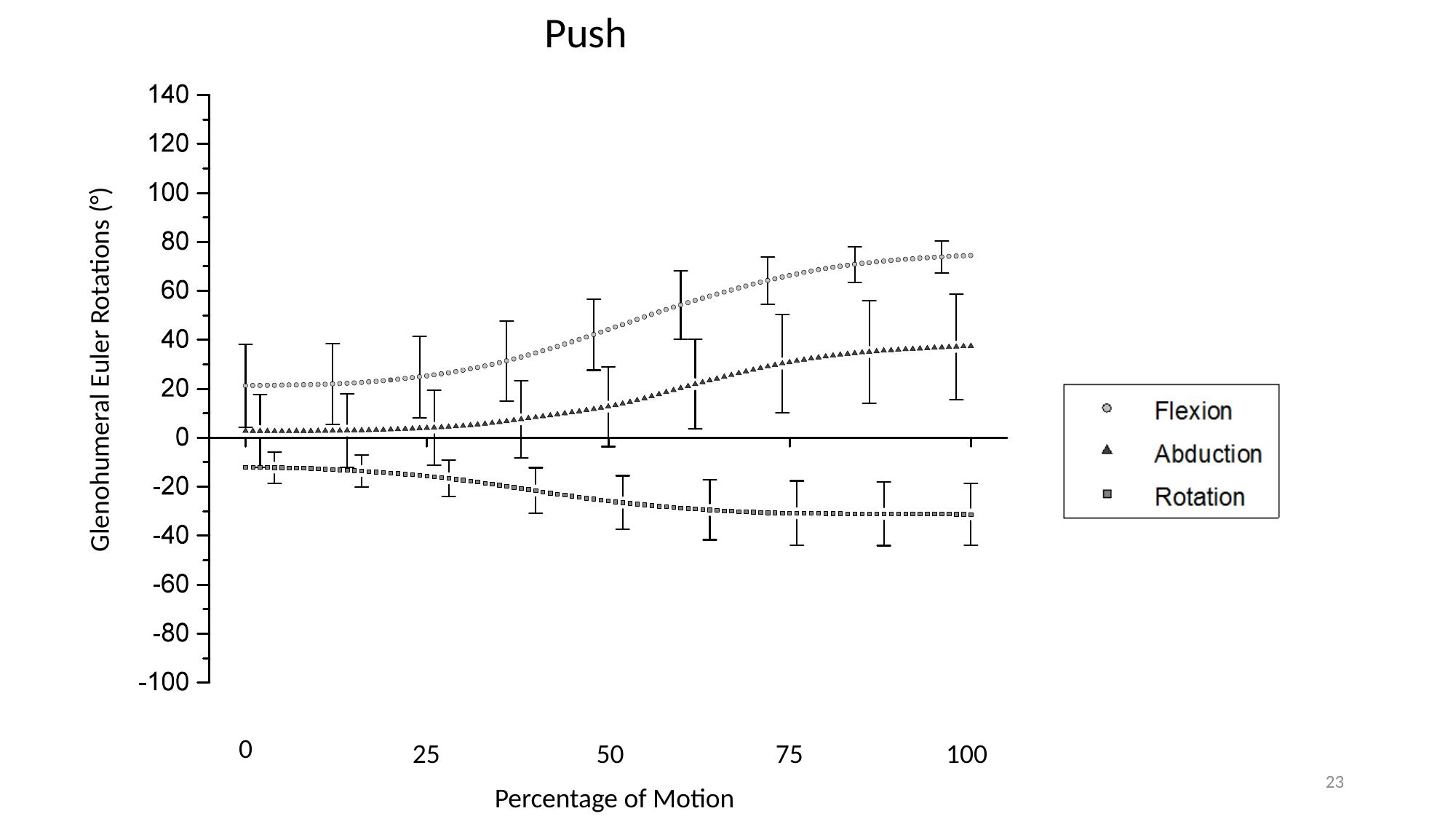

Push
Glenohumeral Euler Rotations (°)
0
25
50
75
100
23
Percentage of Motion

## Slide 24
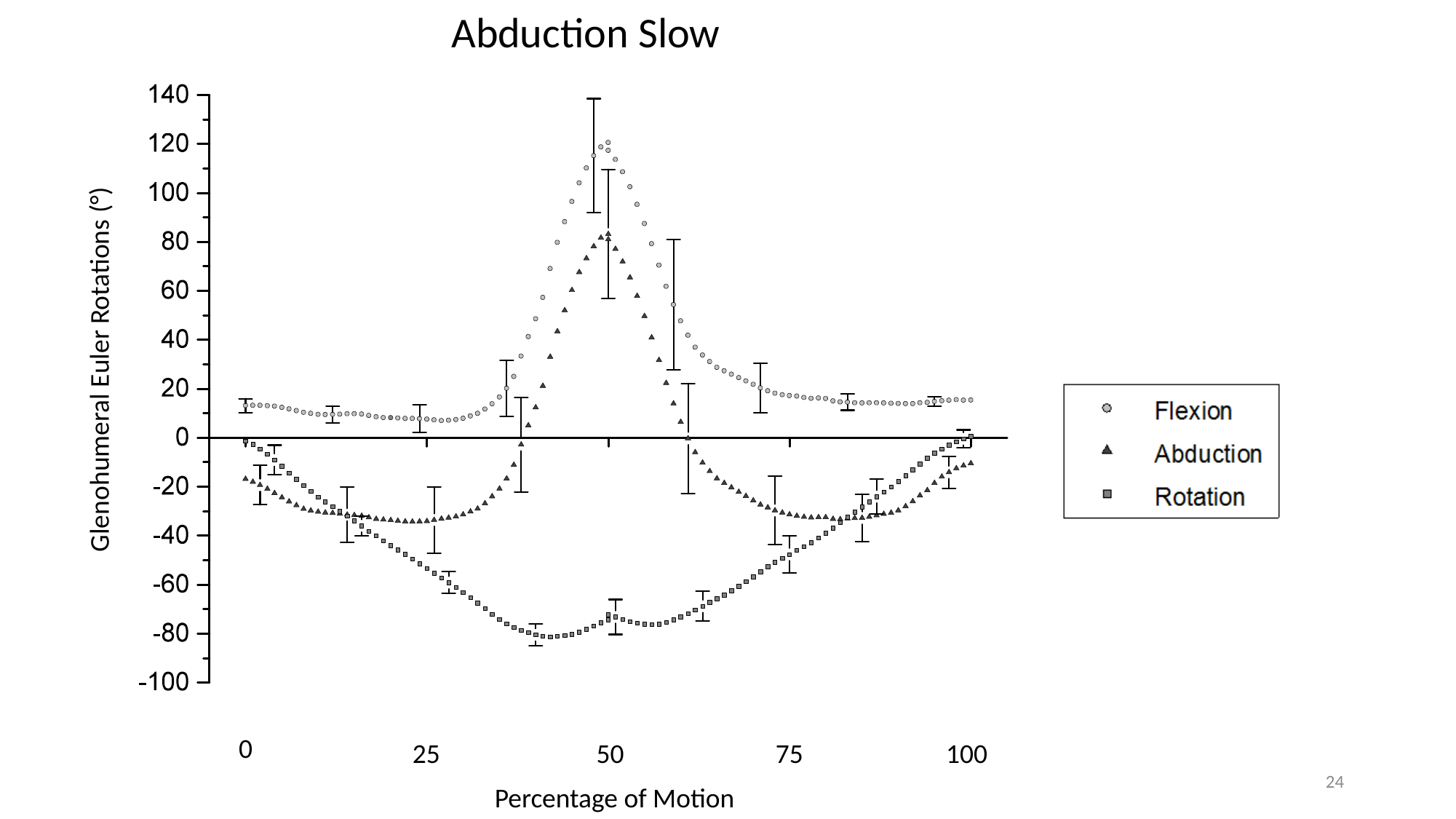

Abduction Slow
Glenohumeral Euler Rotations (°)
0
25
50
75
100
24
Percentage of Motion

## Slide 25
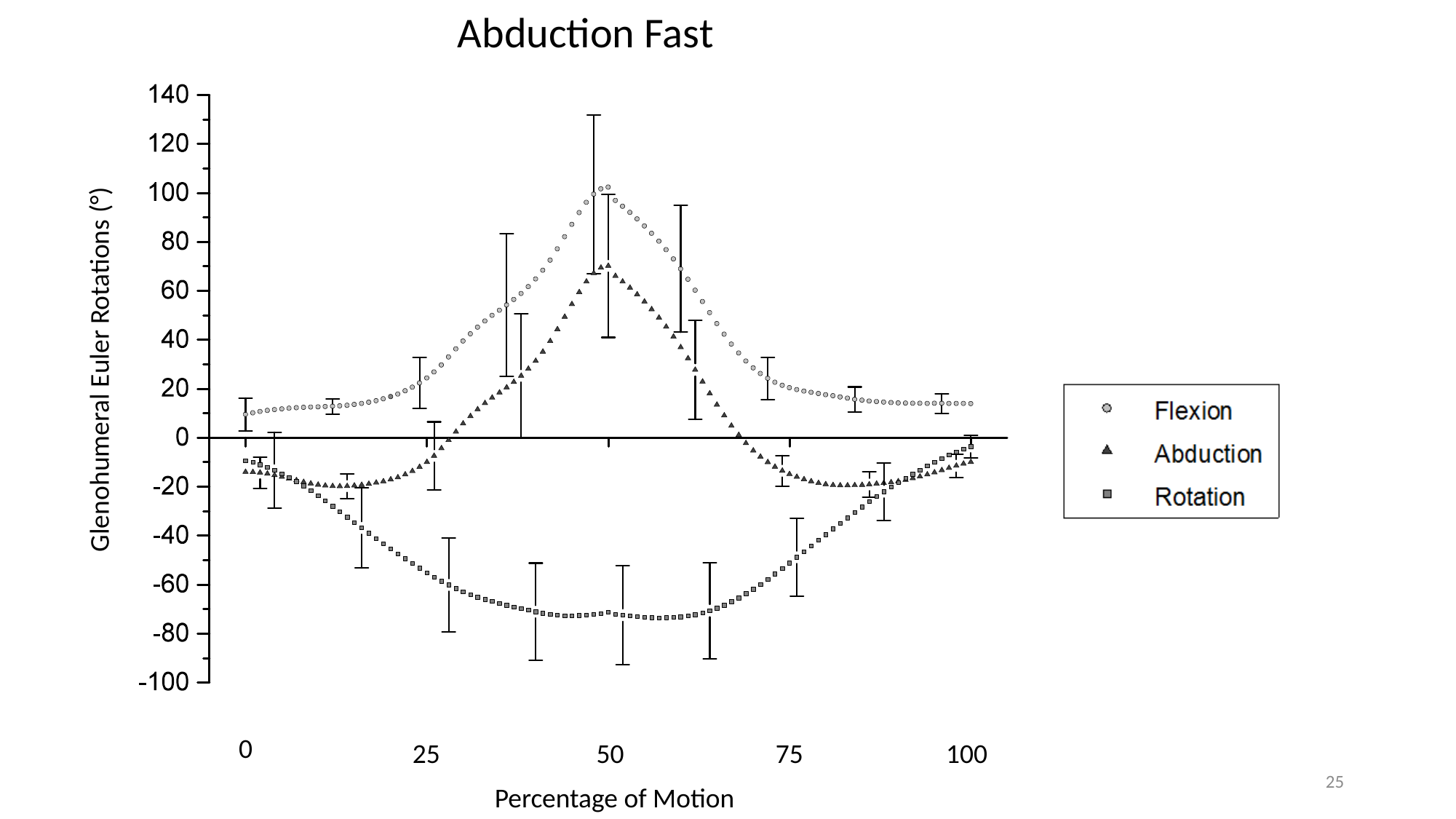

Abduction Fast
Glenohumeral Euler Rotations (°)
0
25
50
75
100
25
Percentage of Motion

## Slide 26
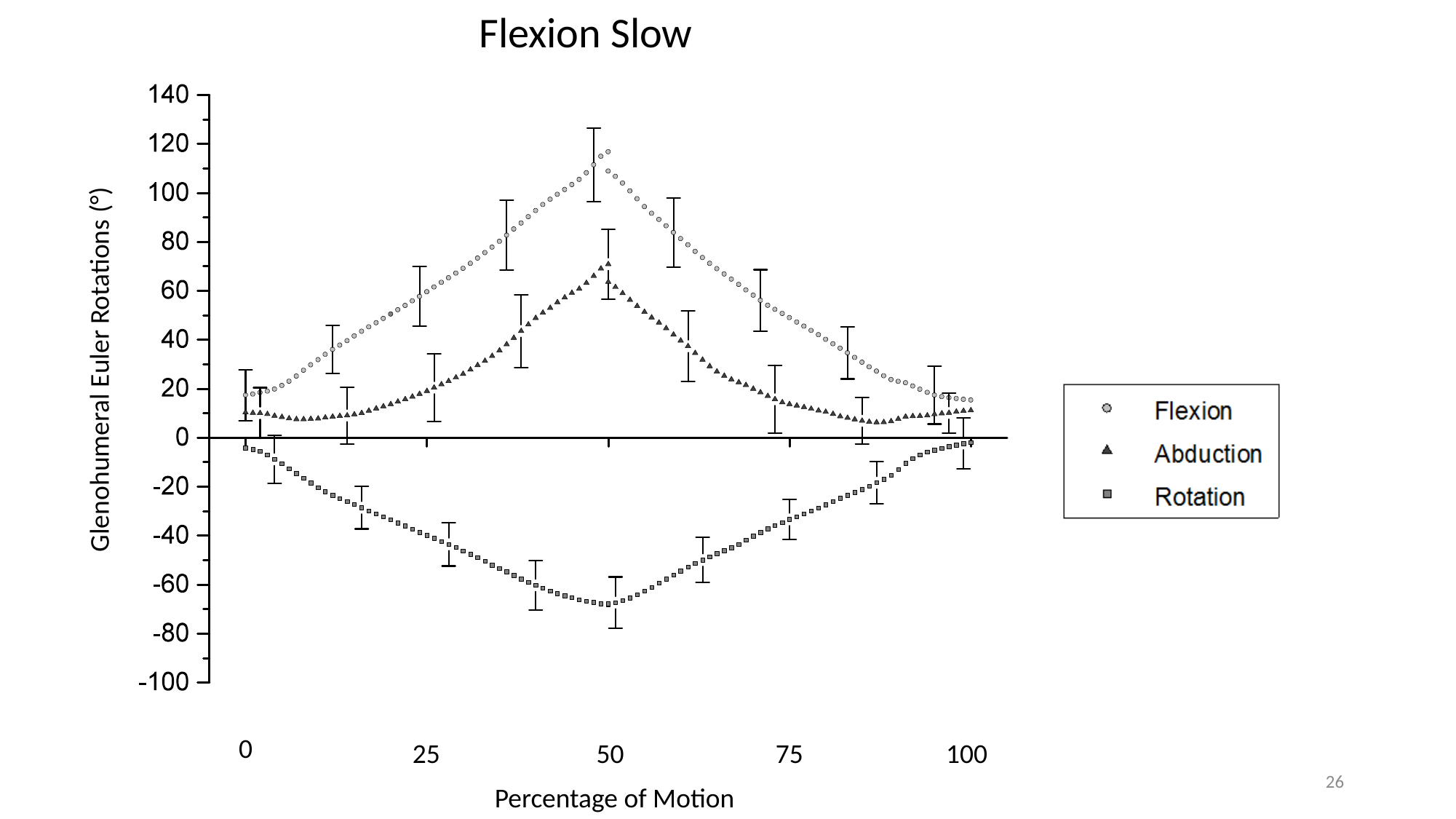

Flexion Slow
Glenohumeral Euler Rotations (°)
0
25
50
75
100
26
Percentage of Motion

## Slide 27
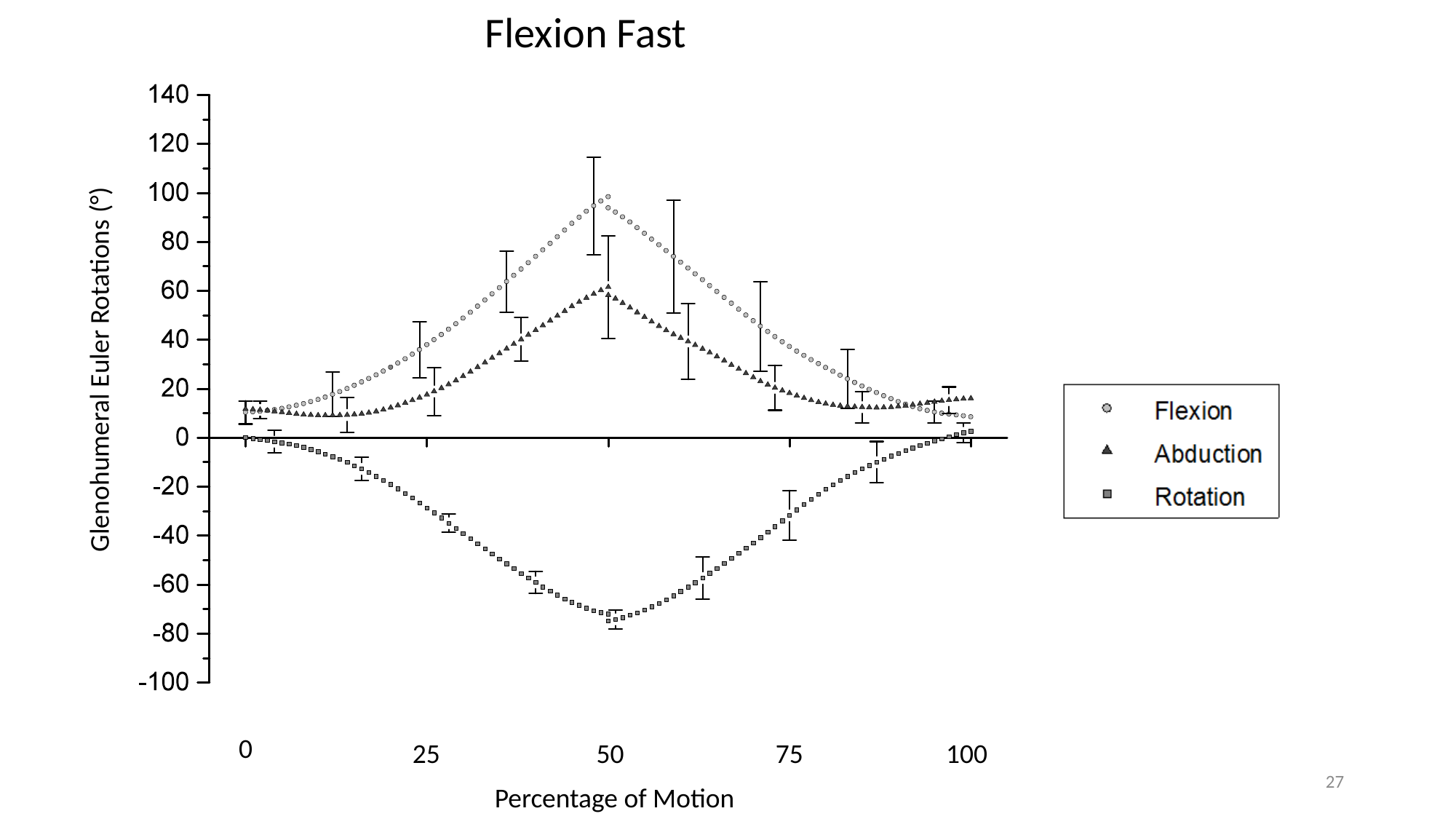

Flexion Fast
Glenohumeral Euler Rotations (°)
0
25
50
75
100
27
Percentage of Motion
