# Supplementary material for: Analysis of shoulder compressive and shear forces during functional activities of daily life
Source: Clin Biomech (Bristol). 2018 May;54:34–41. doi: 10.1016/j.clinbiomech.2018.03.006 (PMC6405441; doi:10.1016/j.clinbiomech.2018.03.006)
Supplement: Supplementary file 2 — Supplementary material 2 [file mmc2.pptx]

## Slide 1
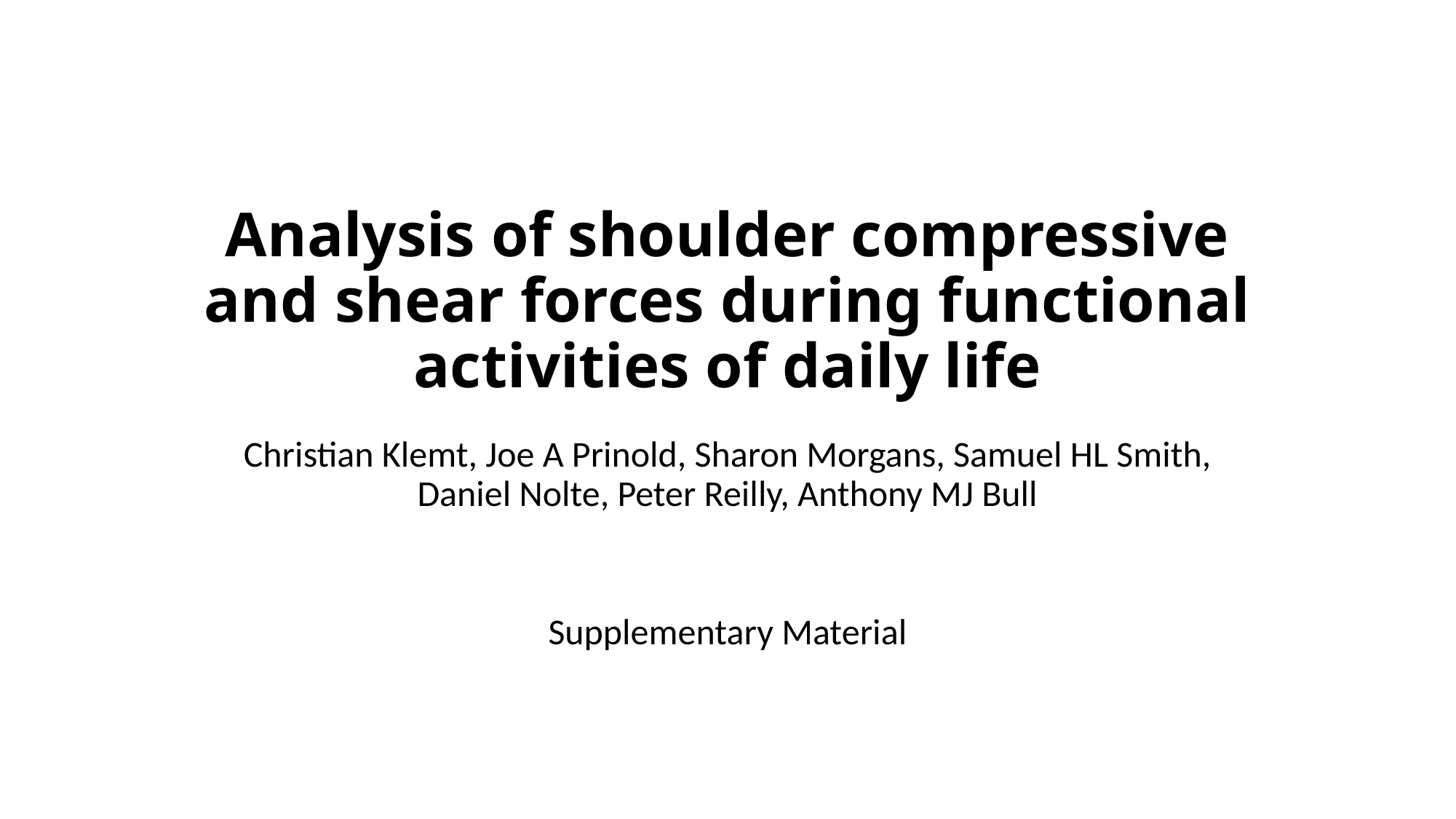

# Analysis of shoulder compressive and shear forces during functional activities of daily life
Christian Klemt, Joe A Prinold, Sharon Morgans, Samuel HL Smith, Daniel Nolte, Peter Reilly, Anthony MJ Bull
Supplementary Material

## Slide 2
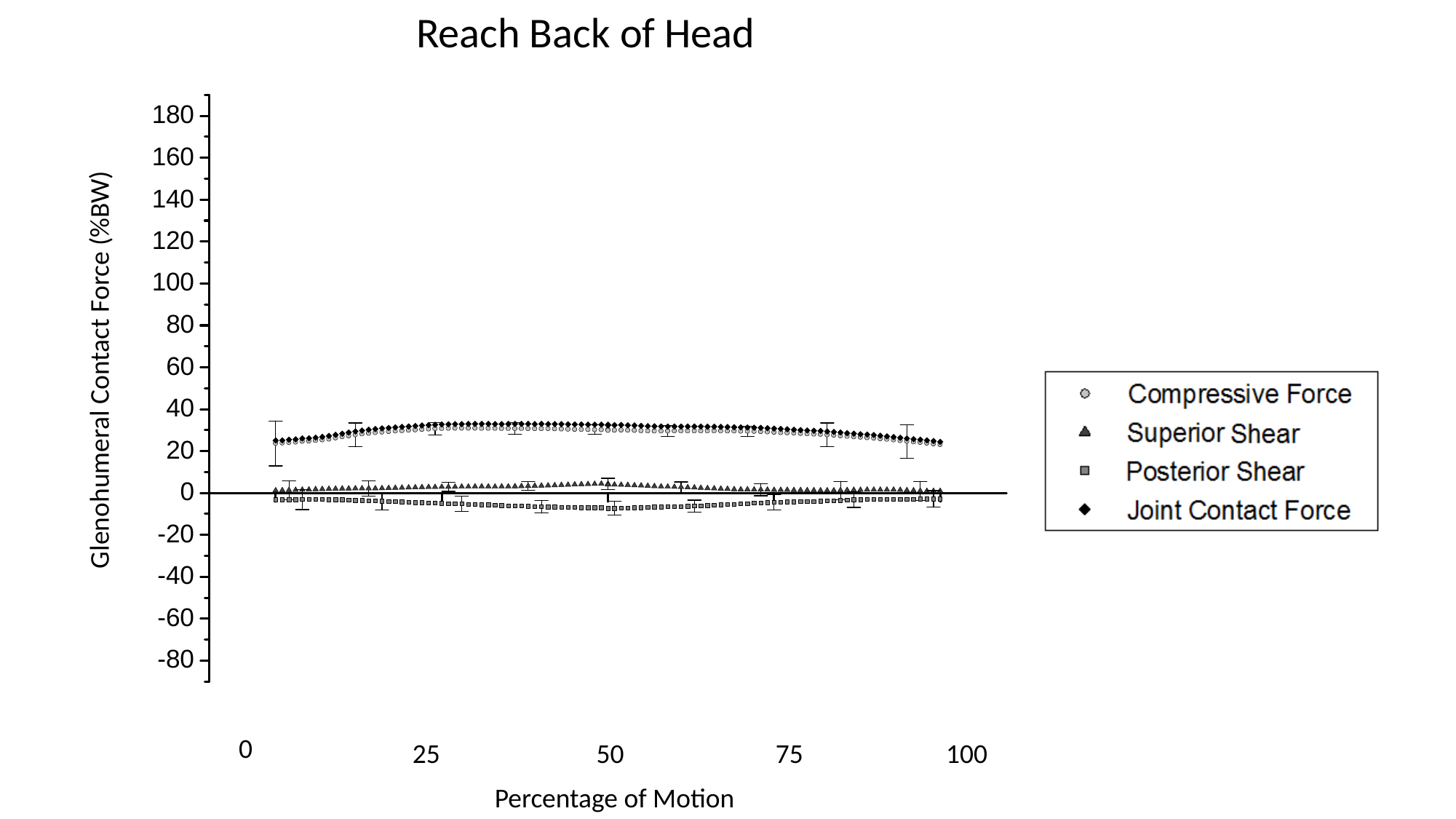

Reach Back of Head
Glenohumeral Contact Force (%BW)
0
25
50
75
100
Percentage of Motion

## Slide 3
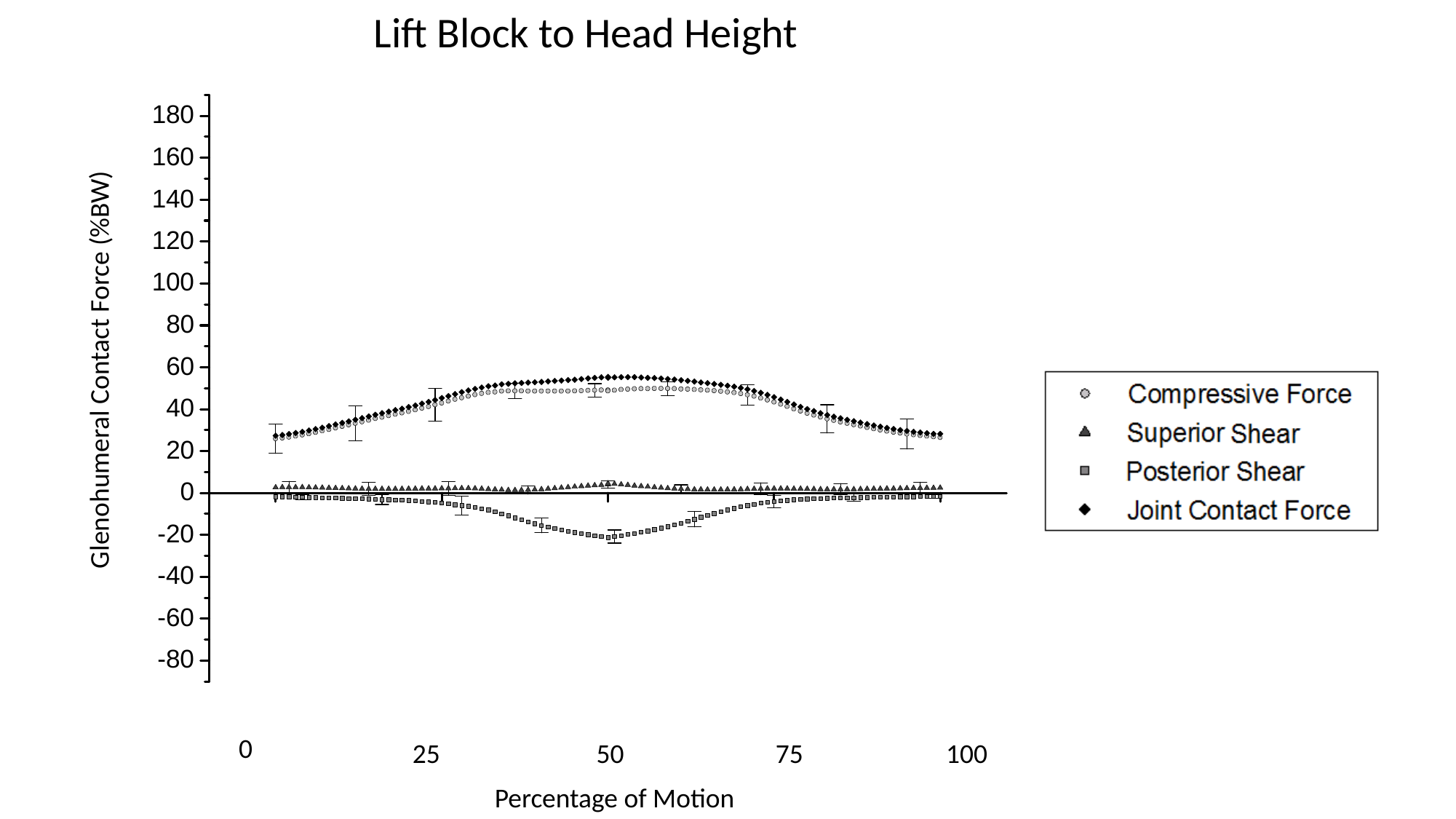

Lift Block to Head Height
Glenohumeral Contact Force (%BW)
0
25
50
75
100
Percentage of Motion

## Slide 4
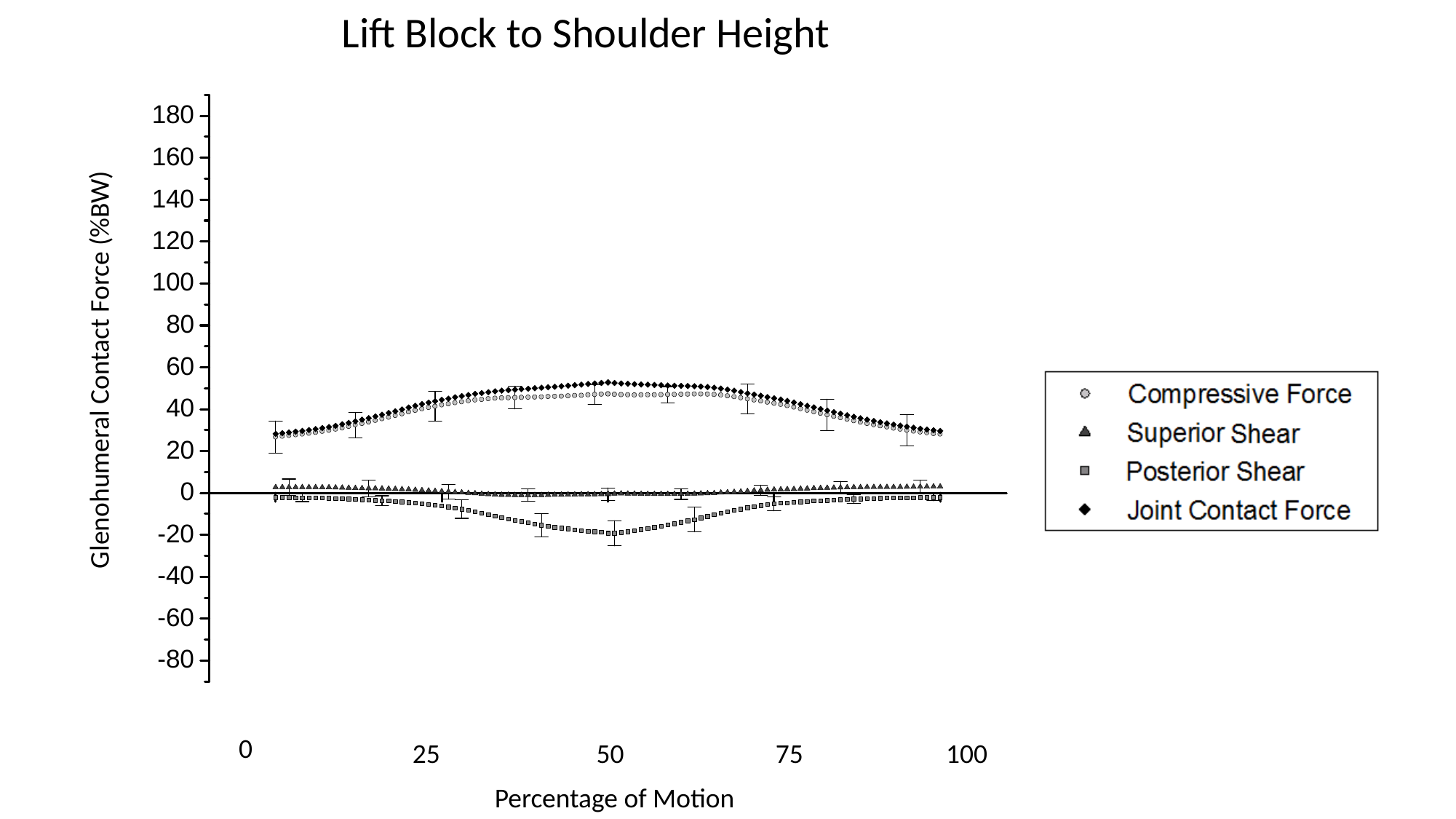

Lift Block to Shoulder Height
Glenohumeral Contact Force (%BW)
0
25
50
75
100
Percentage of Motion

## Slide 5
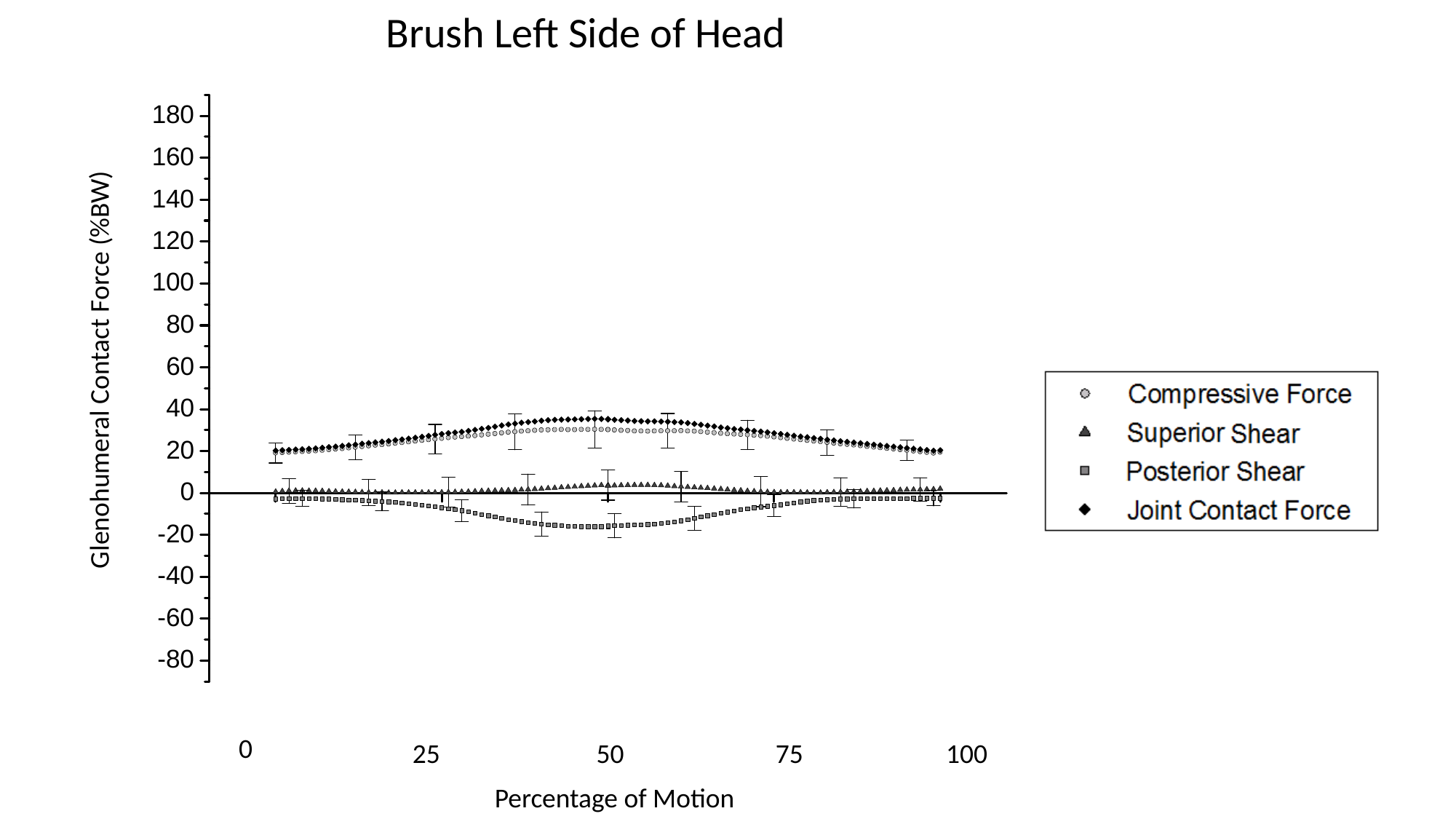

Brush Left Side of Head
Glenohumeral Contact Force (%BW)
0
25
50
75
100
Percentage of Motion

## Slide 6
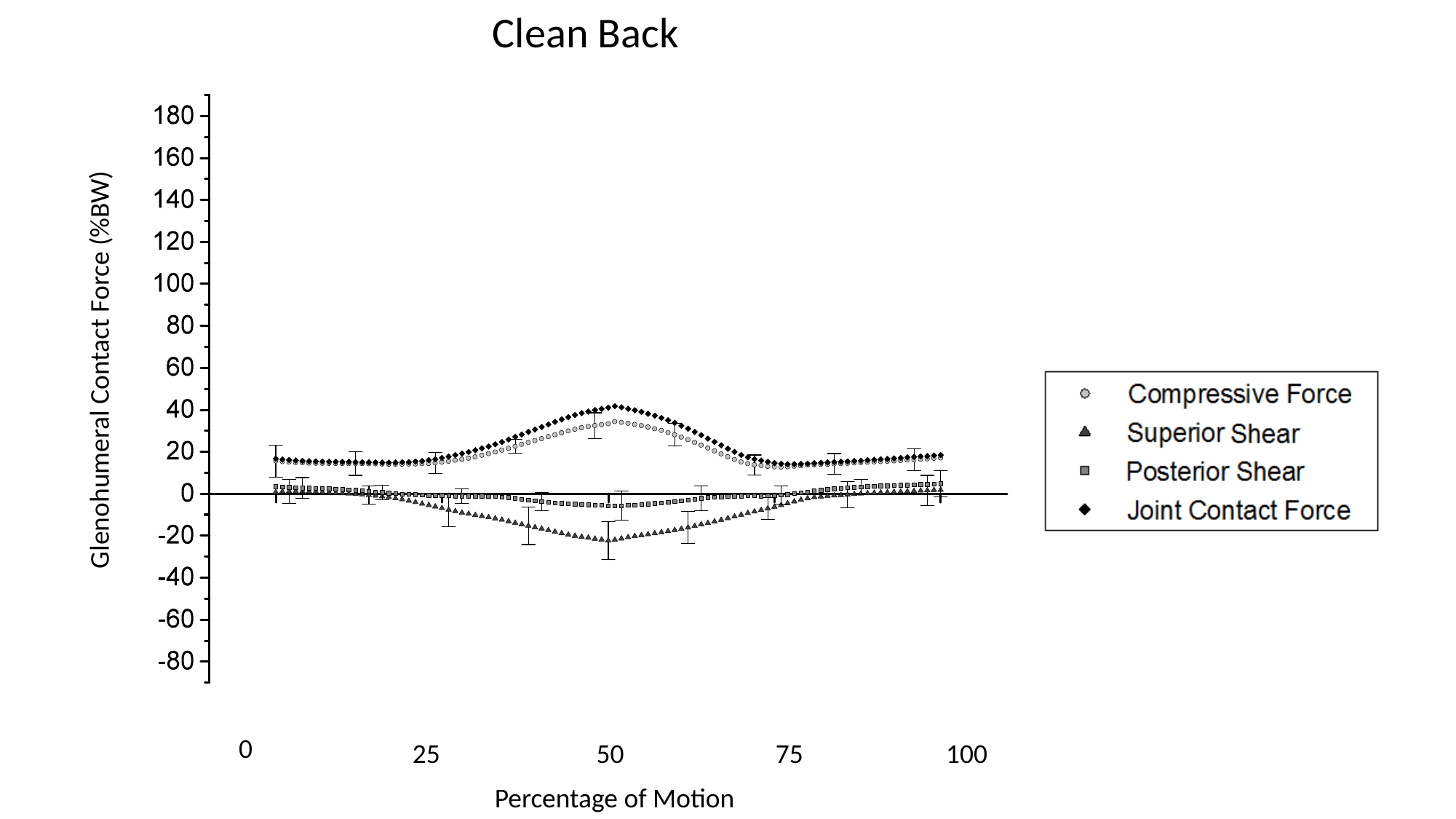

Clean Back
Glenohumeral Contact Force (%BW)
0
25
50
75
100
Percentage of Motion

## Slide 7
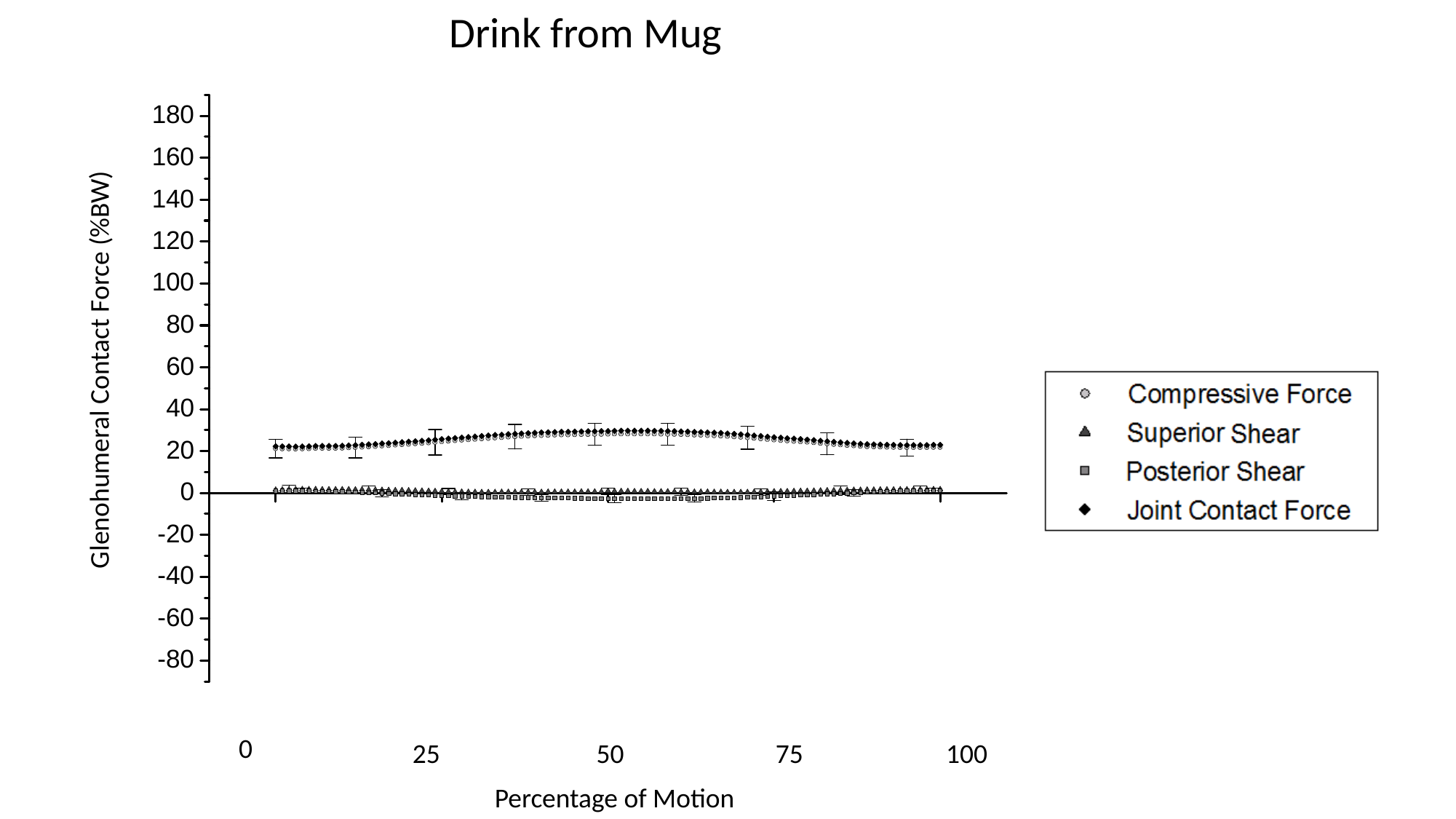

Drink from Mug
Glenohumeral Contact Force (%BW)
0
25
50
75
100
Percentage of Motion

## Slide 8
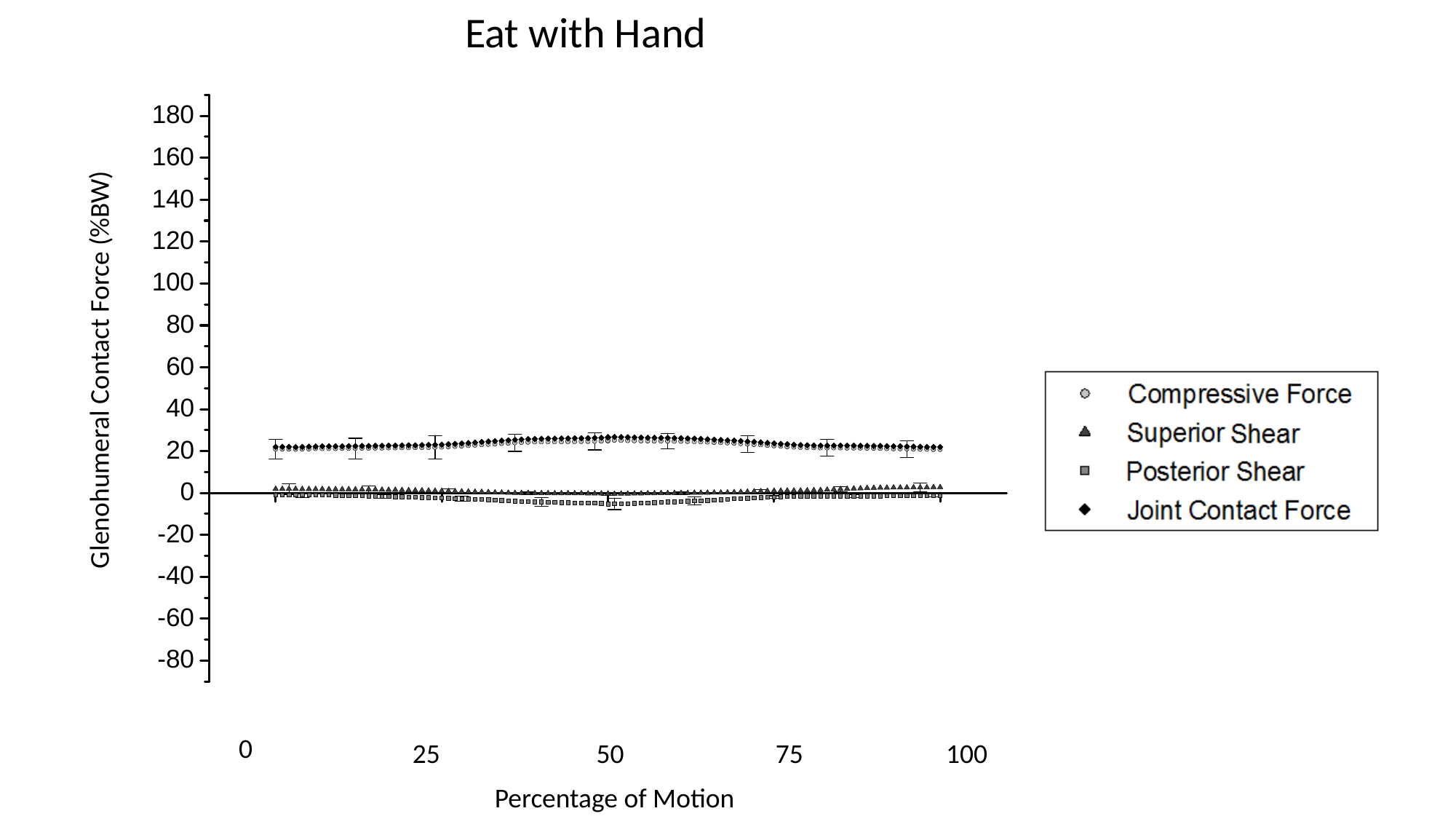

Eat with Hand
Glenohumeral Contact Force (%BW)
0
25
50
75
100
Percentage of Motion

## Slide 9
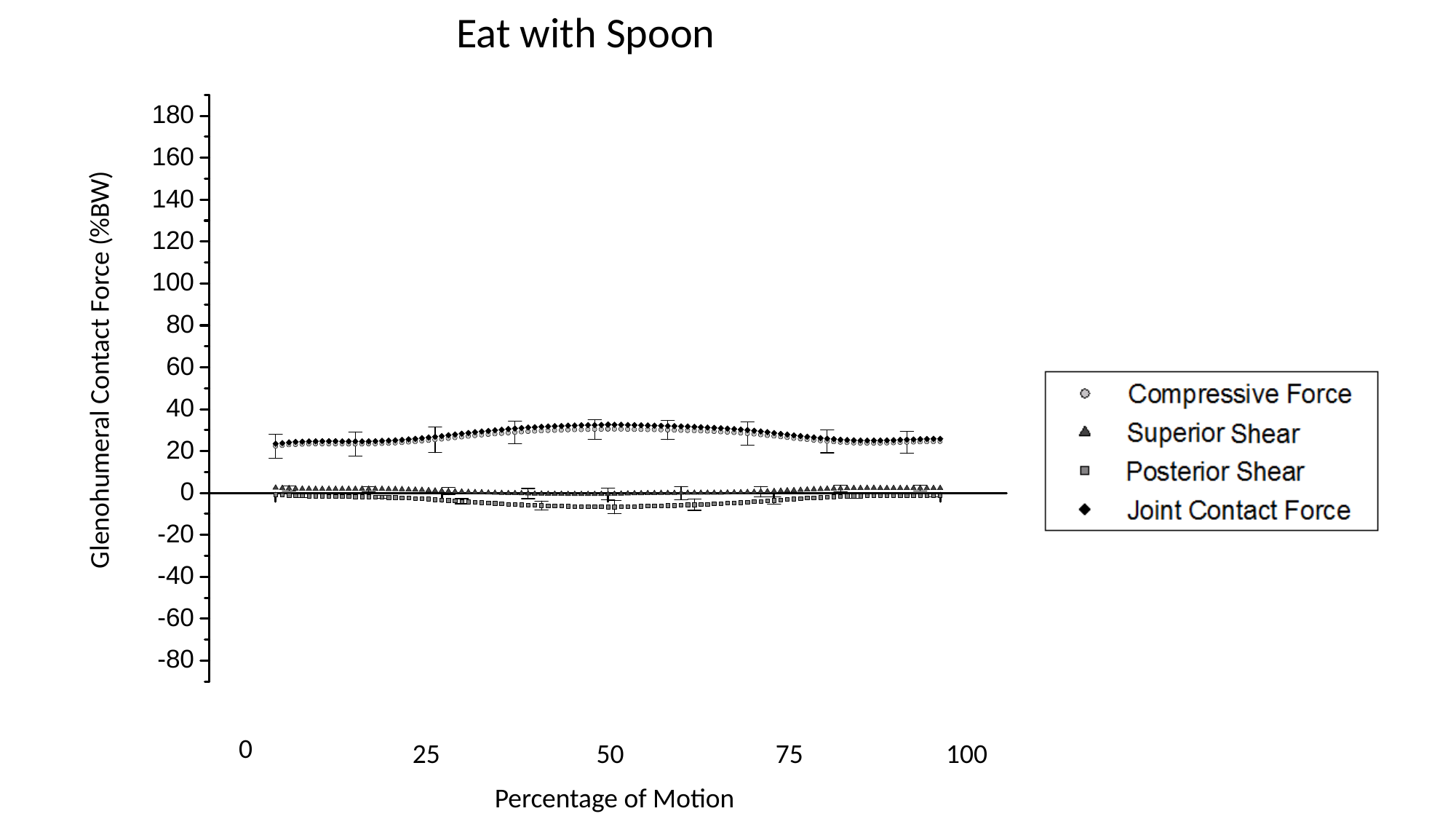

Eat with Spoon
Glenohumeral Contact Force (%BW)
0
25
50
75
100
Percentage of Motion

## Slide 10
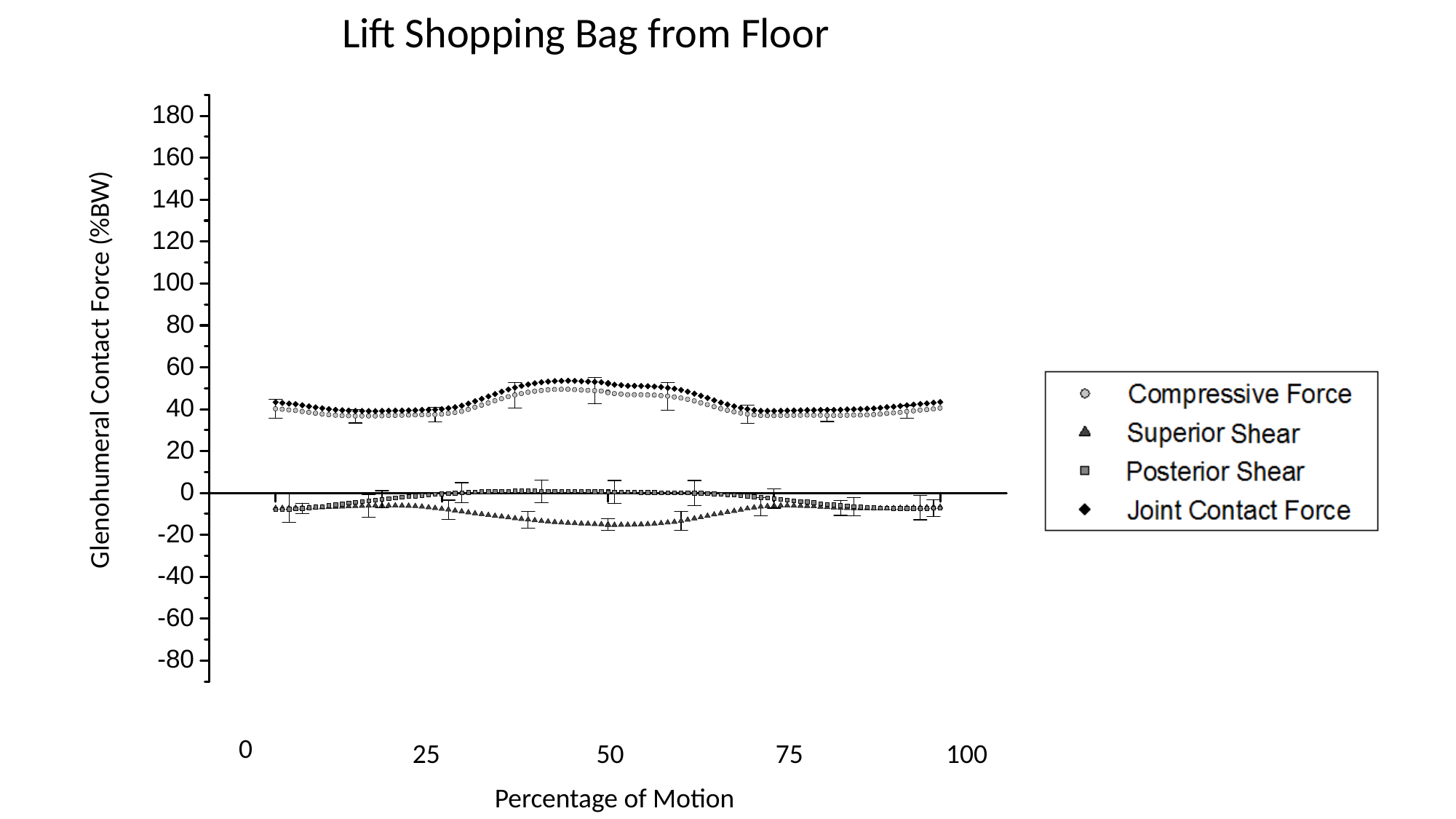

Lift Shopping Bag from Floor
Glenohumeral Contact Force (%BW)
0
25
50
75
100
Percentage of Motion

## Slide 11
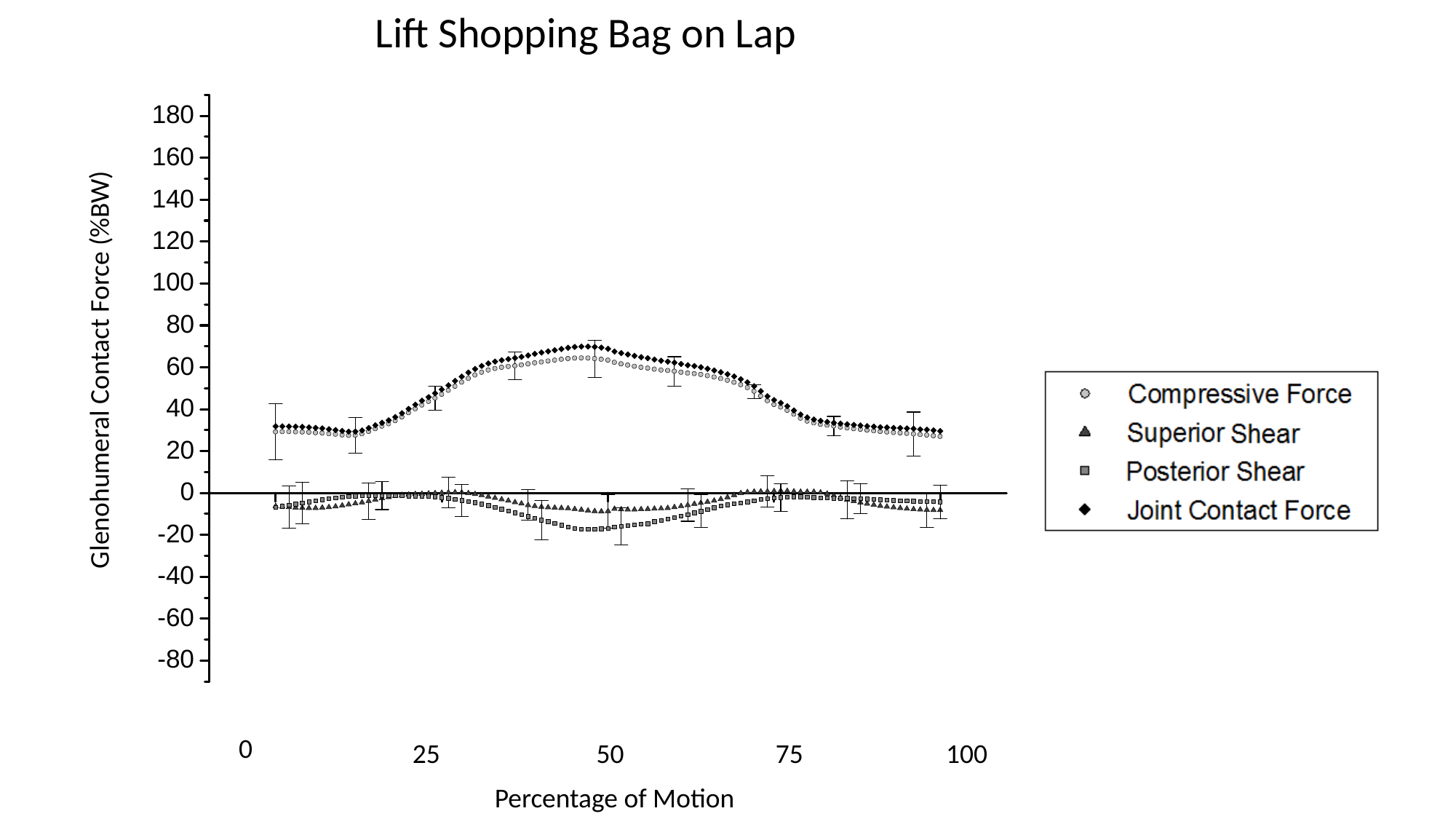

Lift Shopping Bag on Lap
Glenohumeral Contact Force (%BW)
0
25
50
75
100
Percentage of Motion

## Slide 12
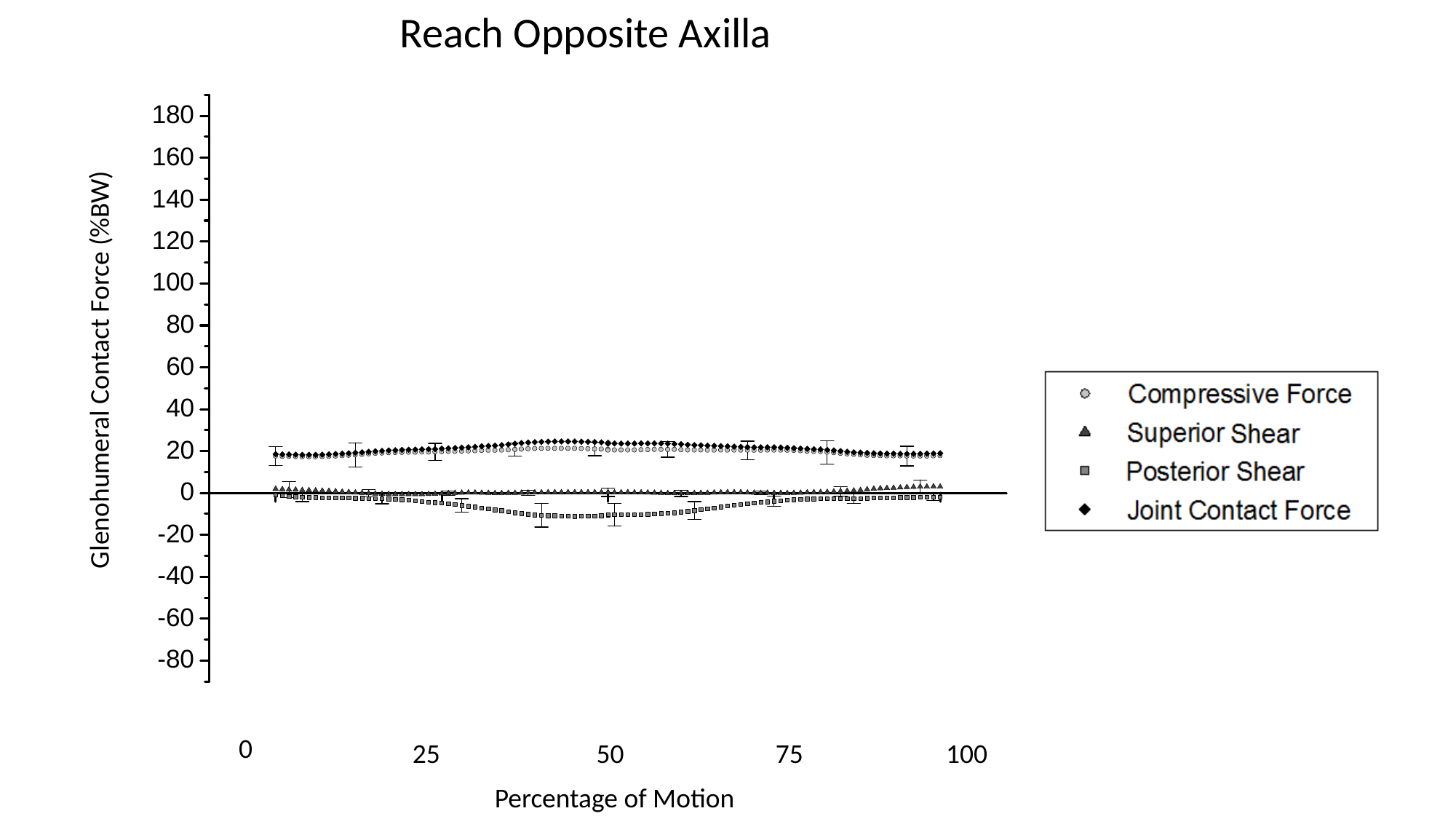

Reach Opposite Axilla
Glenohumeral Contact Force (%BW)
0
25
50
75
100
Percentage of Motion

## Slide 13
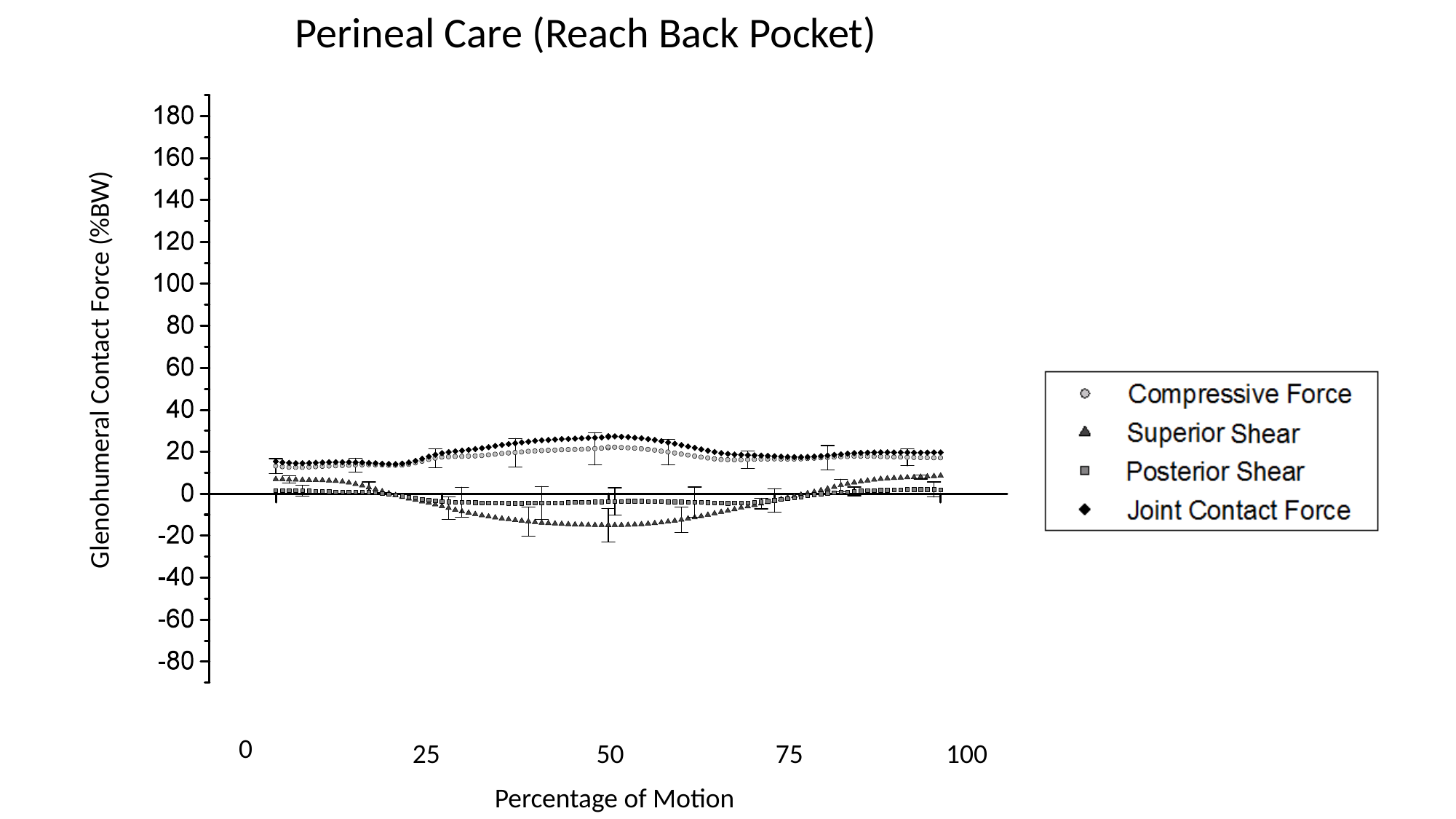

Perineal Care (Reach Back Pocket)
Glenohumeral Contact Force (%BW)
0
25
50
75
100
Percentage of Motion

## Slide 14
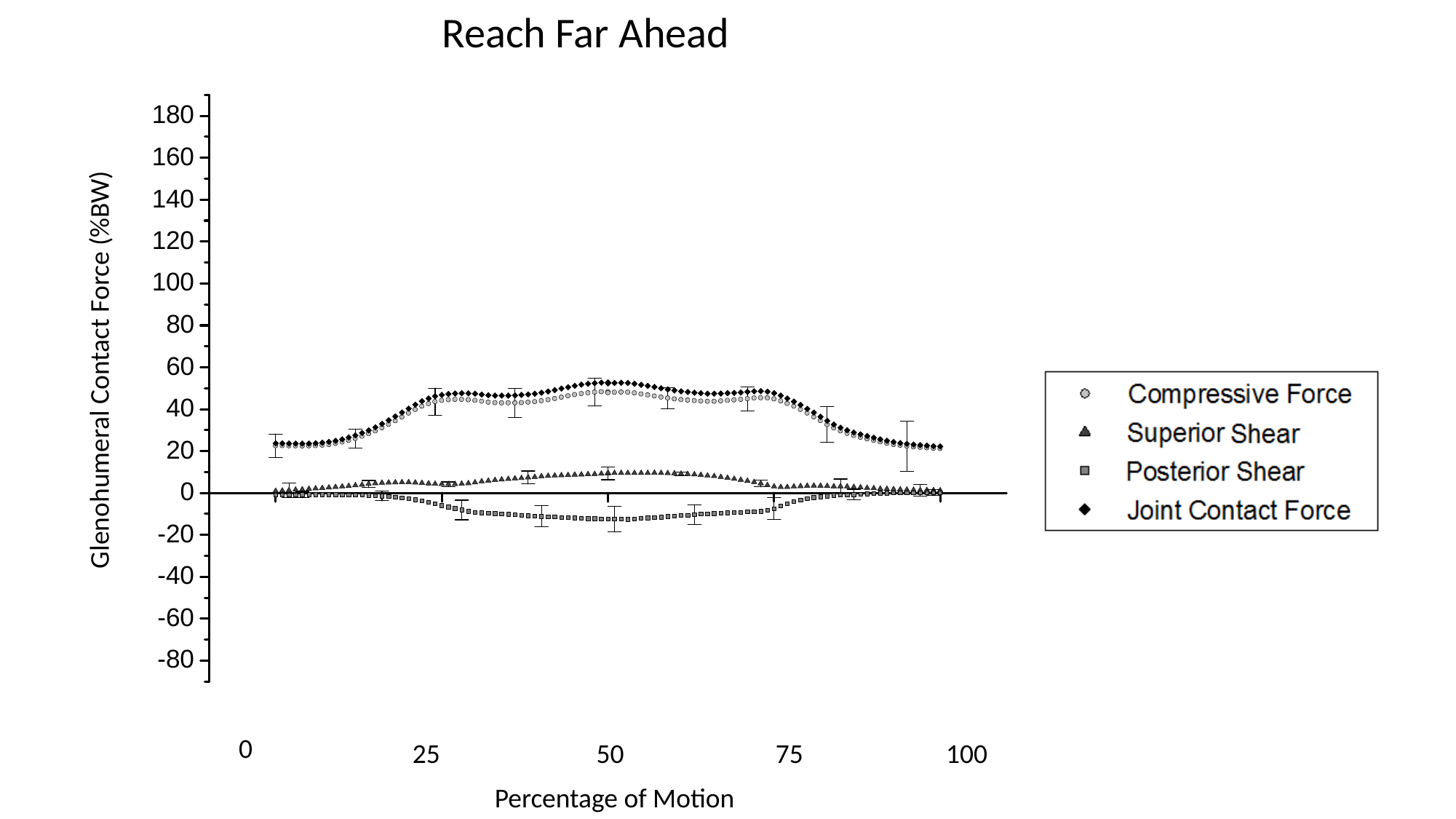

Reach Far Ahead
Glenohumeral Contact Force (%BW)
0
25
50
75
100
Percentage of Motion

## Slide 15
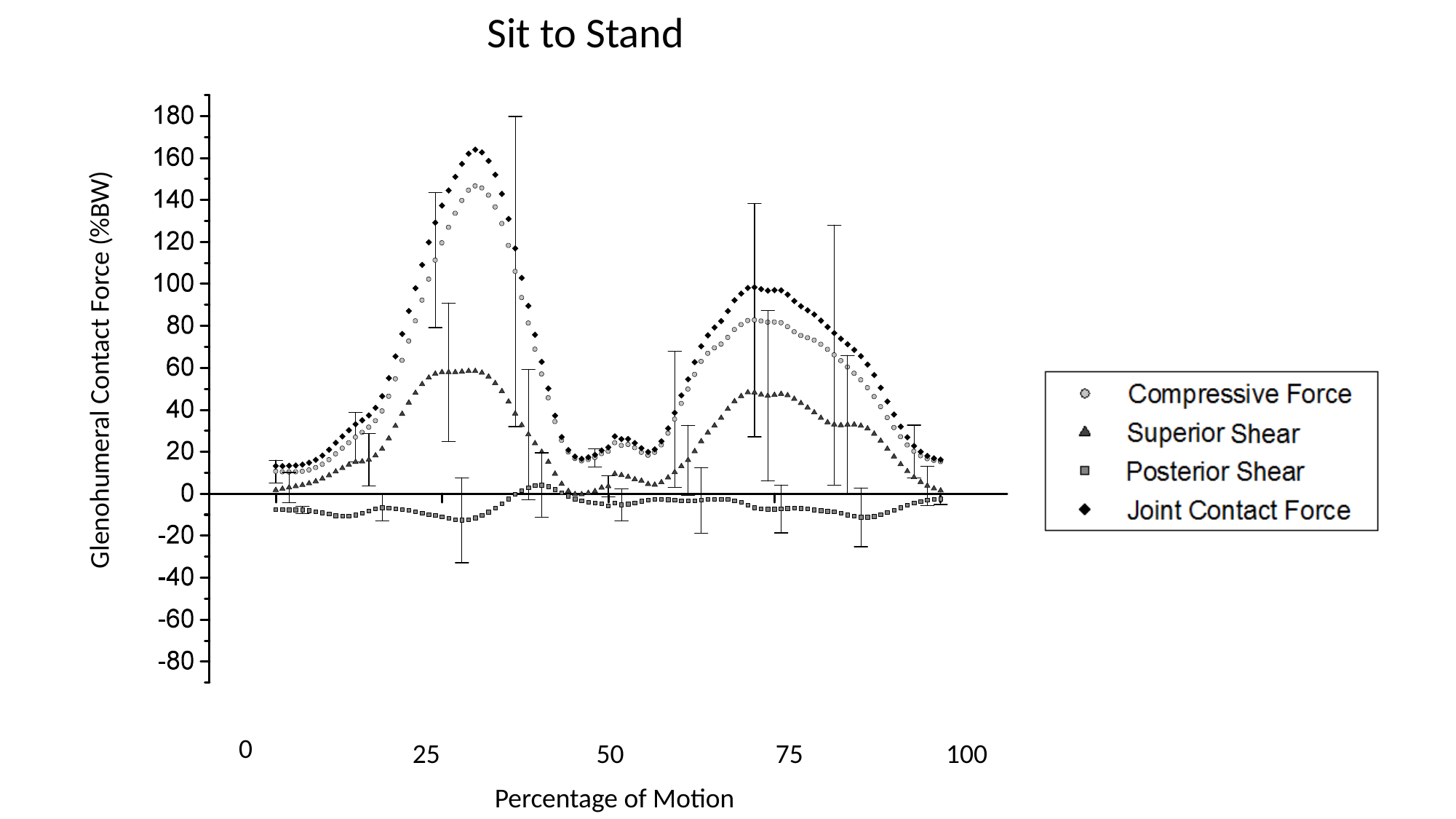

Sit to Stand
Glenohumeral Contact Force (%BW)
0
25
50
75
100
Percentage of Motion

## Slide 16
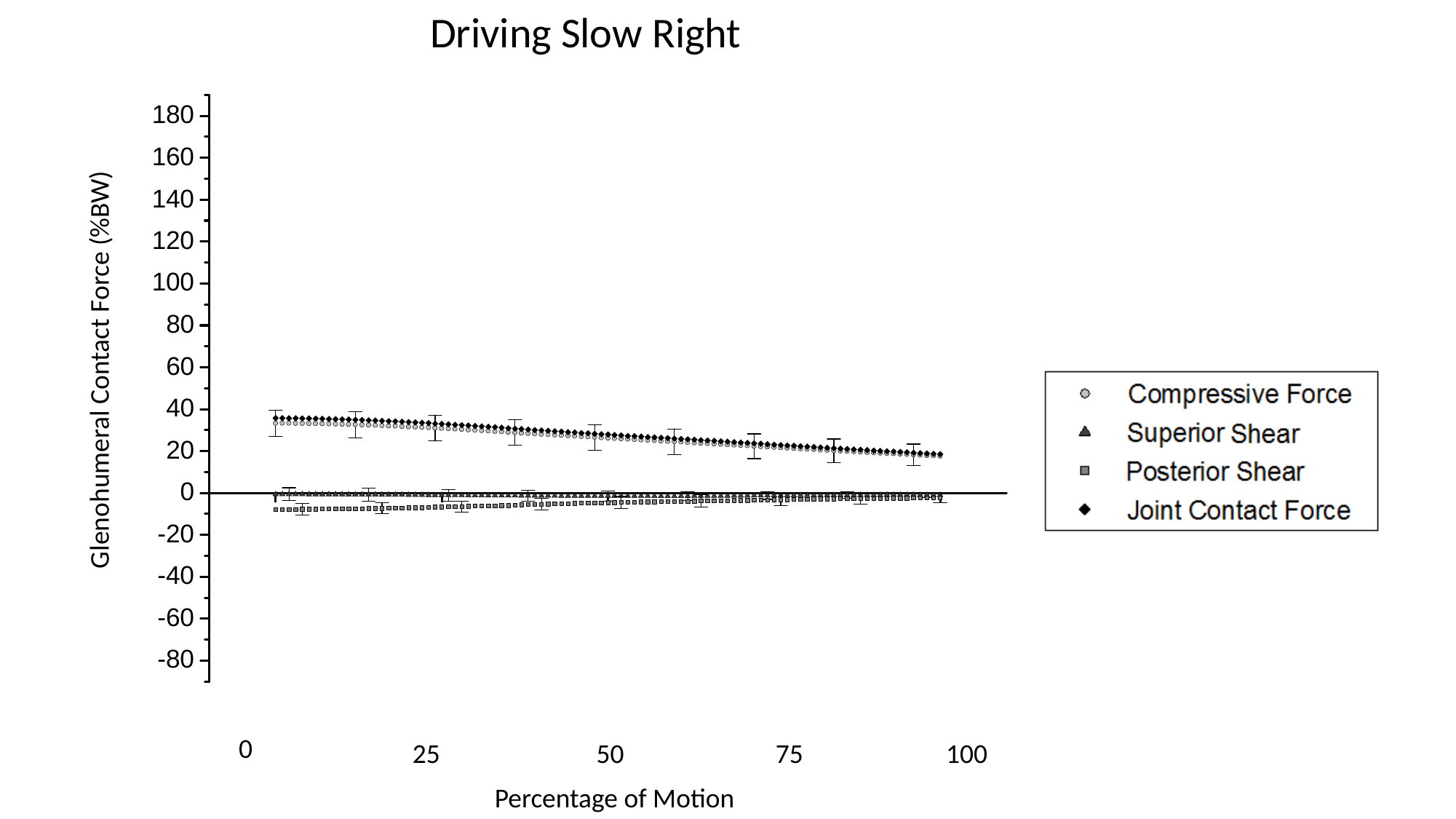

Driving Slow Right
Glenohumeral Contact Force (%BW)
0
25
50
75
100
Percentage of Motion

## Slide 17
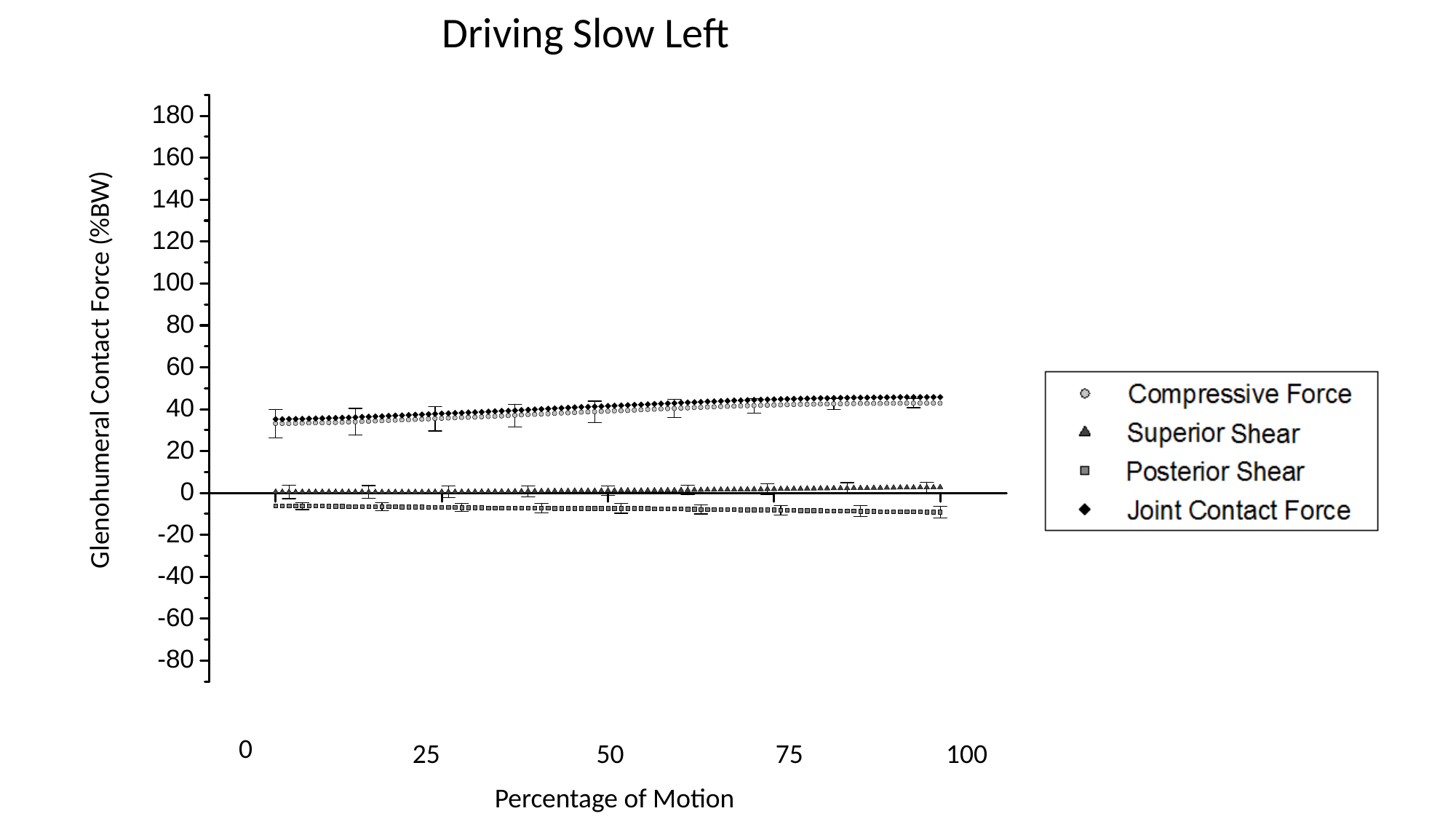

Driving Slow Left
Glenohumeral Contact Force (%BW)
0
25
50
75
100
Percentage of Motion

## Slide 18
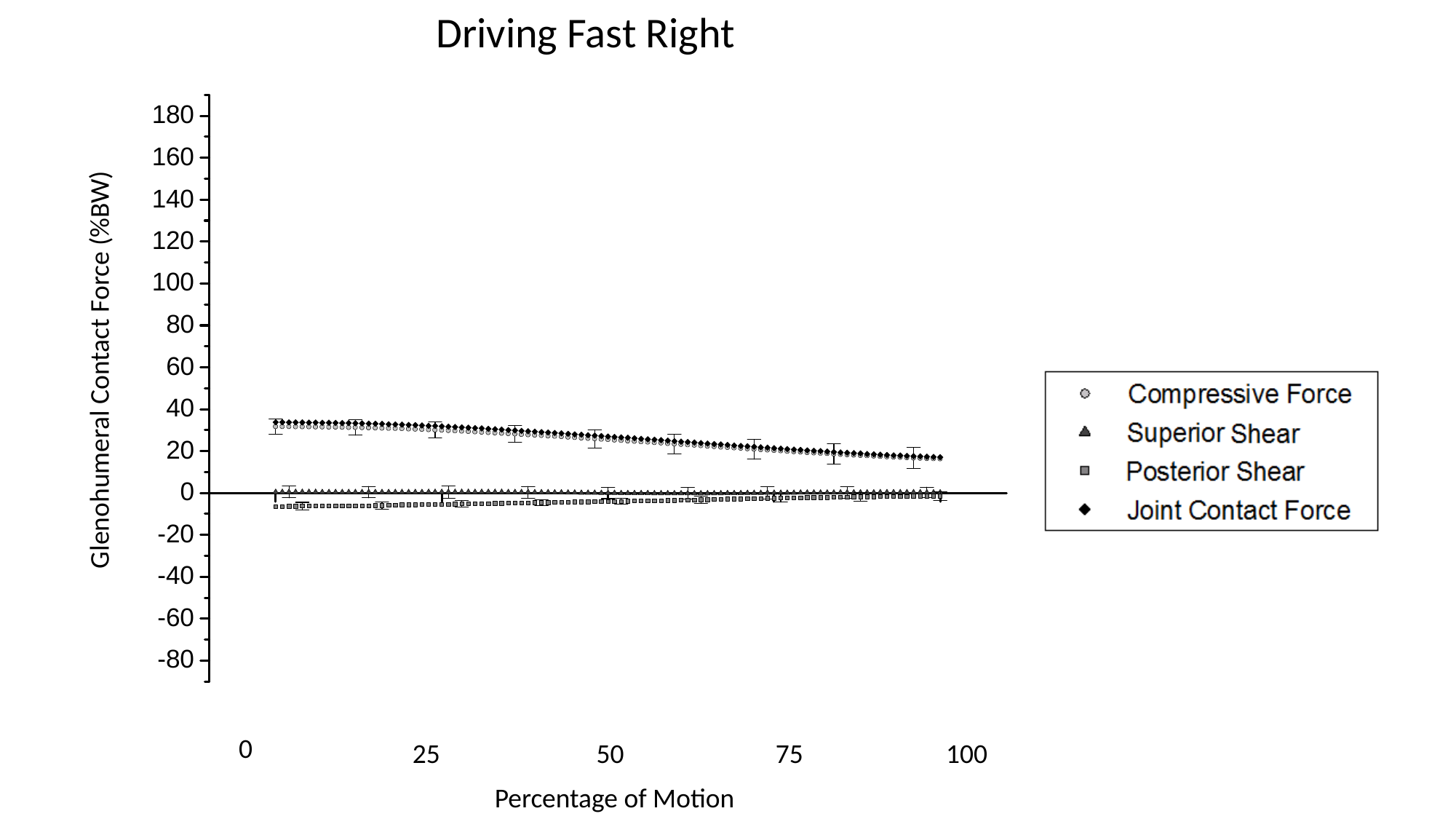

Driving Fast Right
Glenohumeral Contact Force (%BW)
0
25
50
75
100
Percentage of Motion

## Slide 19
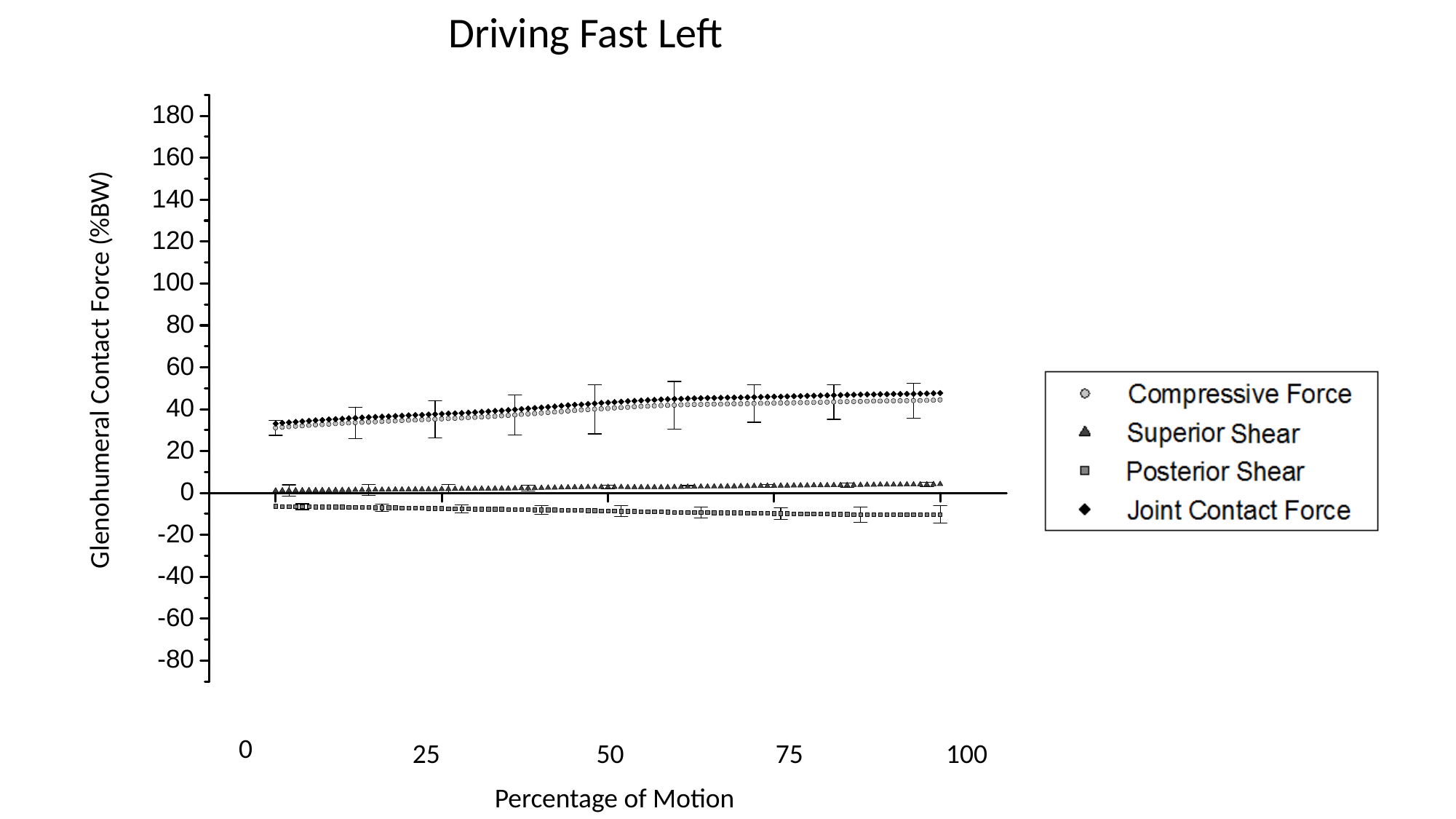

Driving Fast Left
Glenohumeral Contact Force (%BW)
0
25
50
75
100
Percentage of Motion

## Slide 20
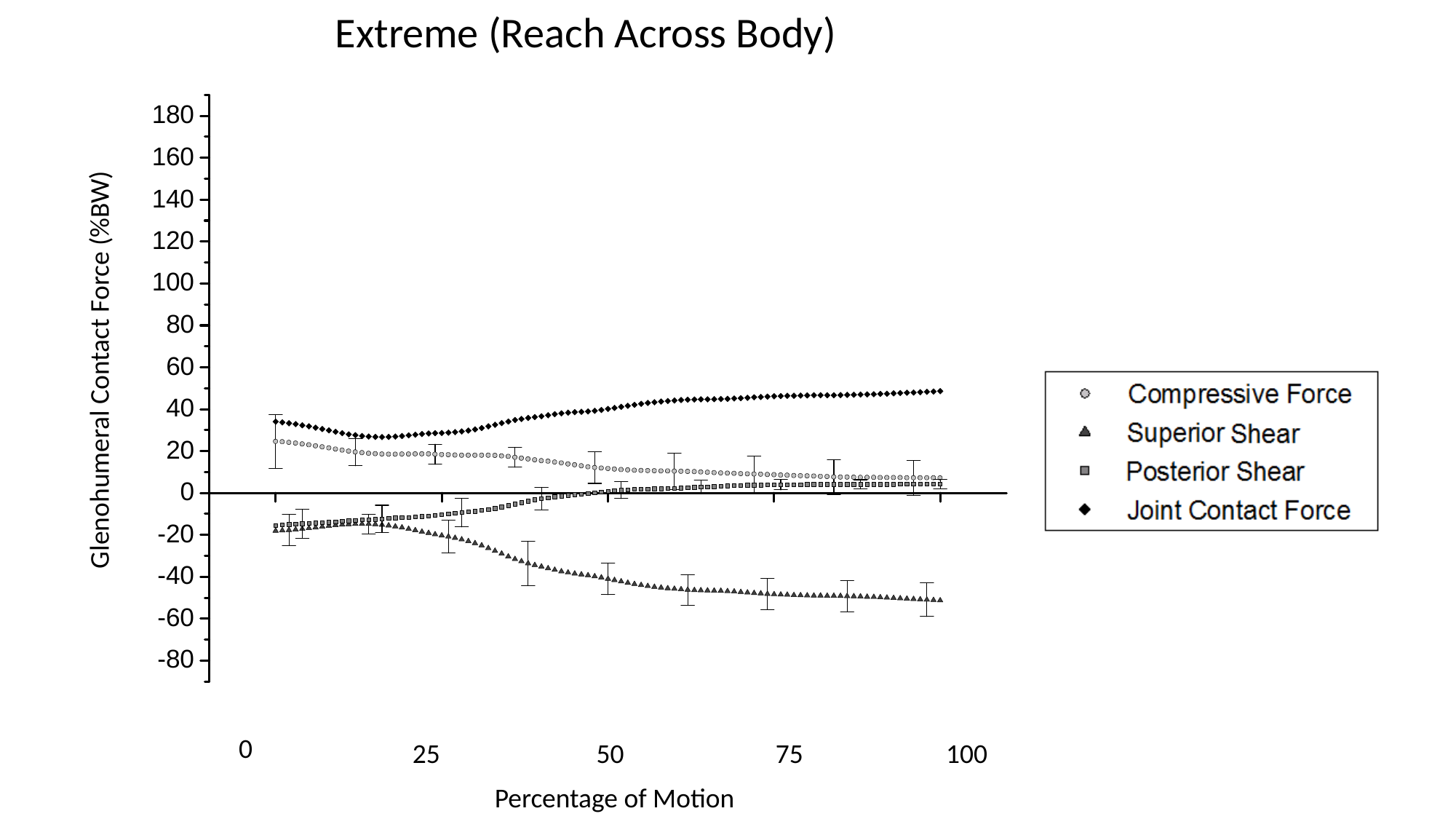

Extreme (Reach Across Body)
Glenohumeral Contact Force (%BW)
0
25
50
75
100
Percentage of Motion

## Slide 21
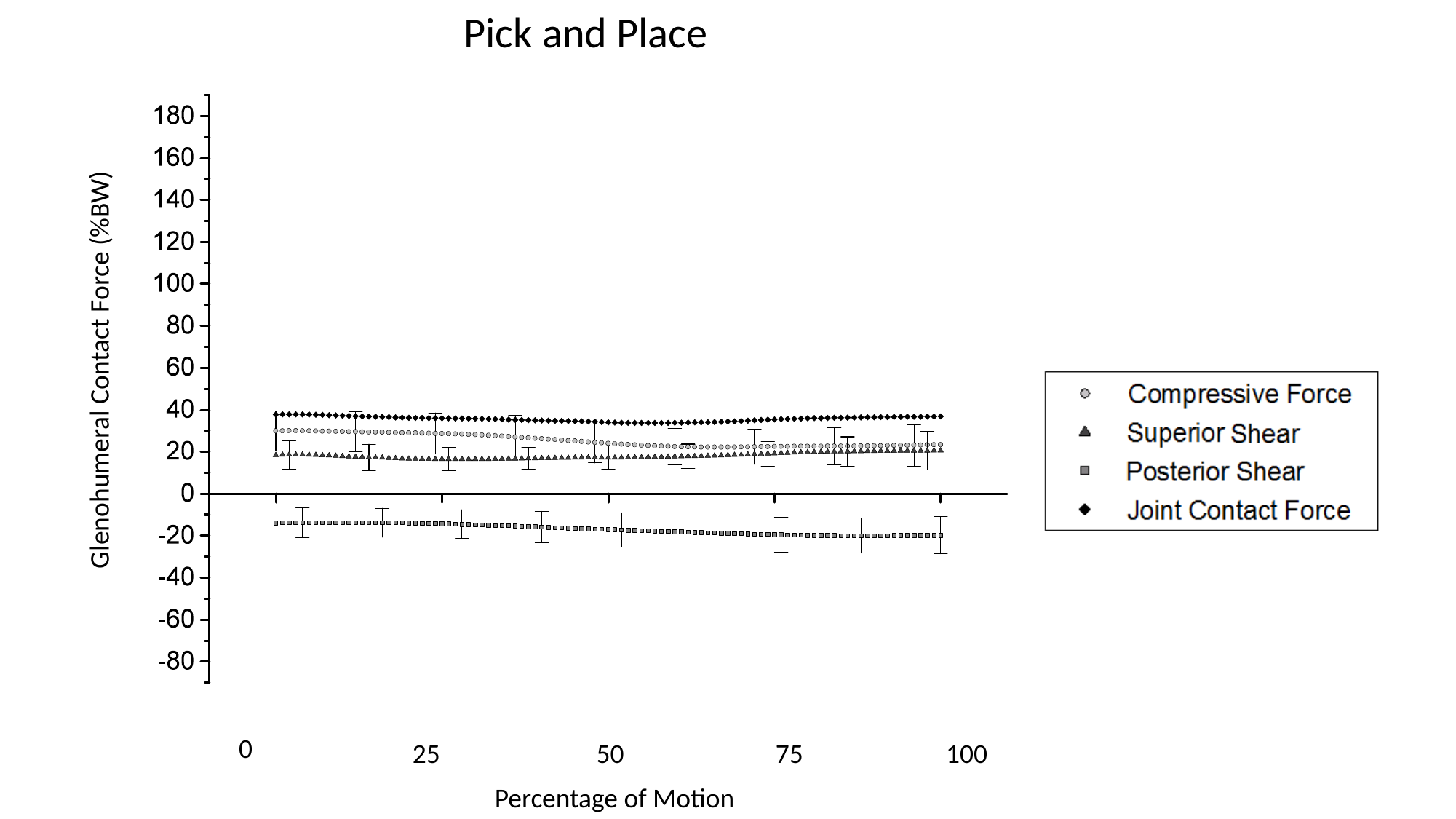

Pick and Place
Glenohumeral Contact Force (%BW)
0
25
50
75
100
Percentage of Motion

## Slide 22
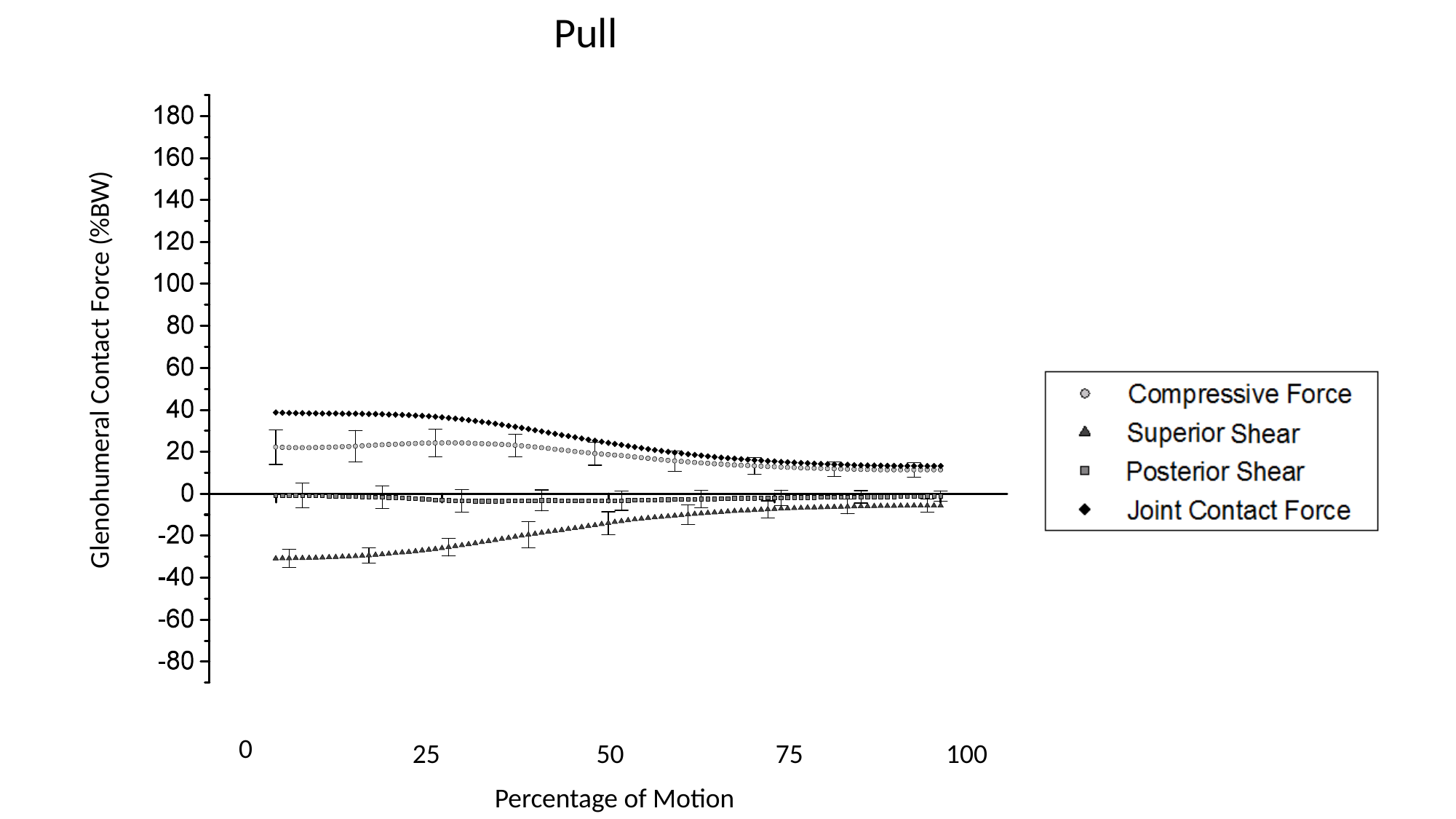

Pull
Glenohumeral Contact Force (%BW)
0
25
50
75
100
Percentage of Motion

## Slide 23
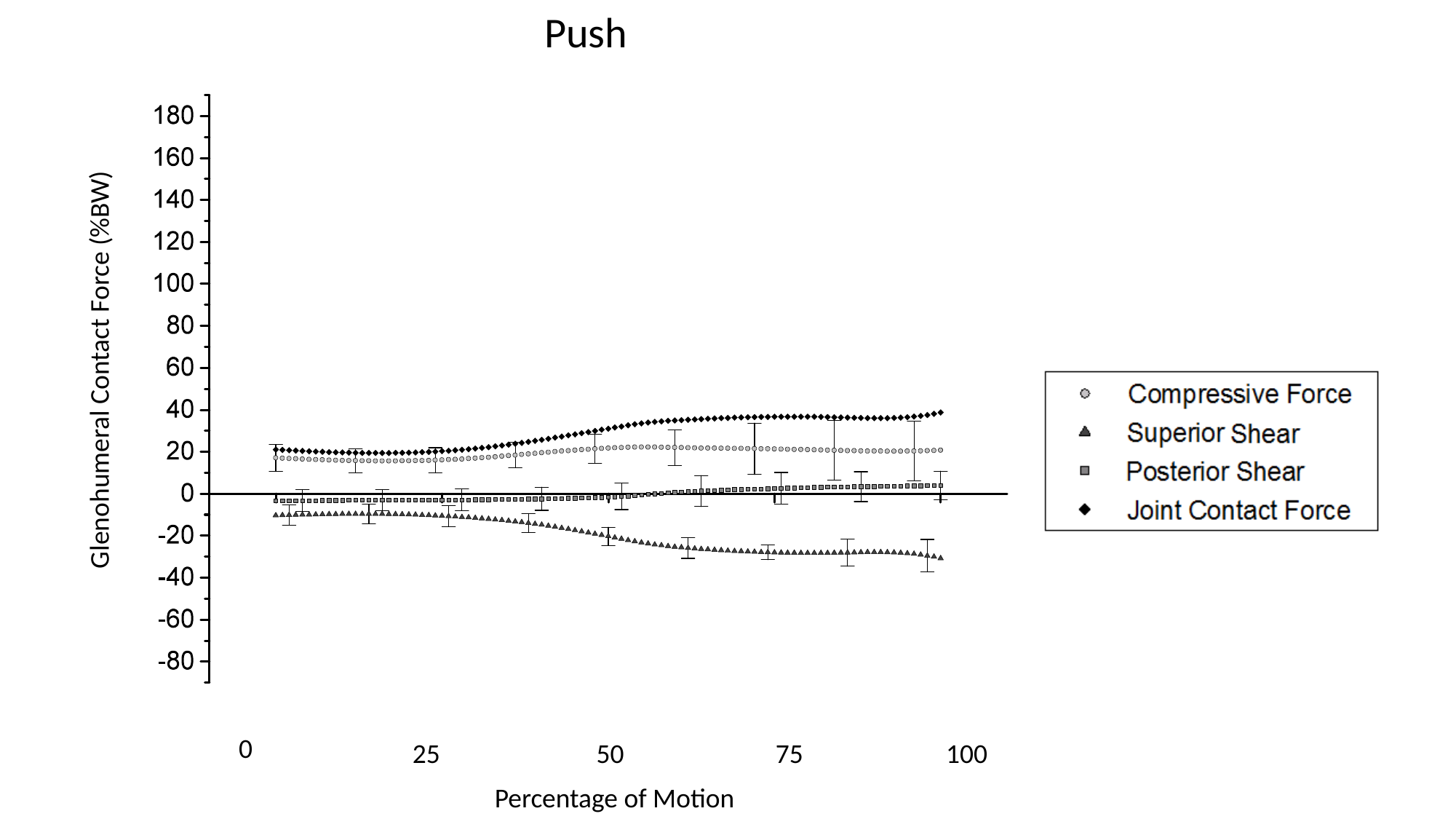

Push
Glenohumeral Contact Force (%BW)
0
25
50
75
100
Percentage of Motion

## Slide 24
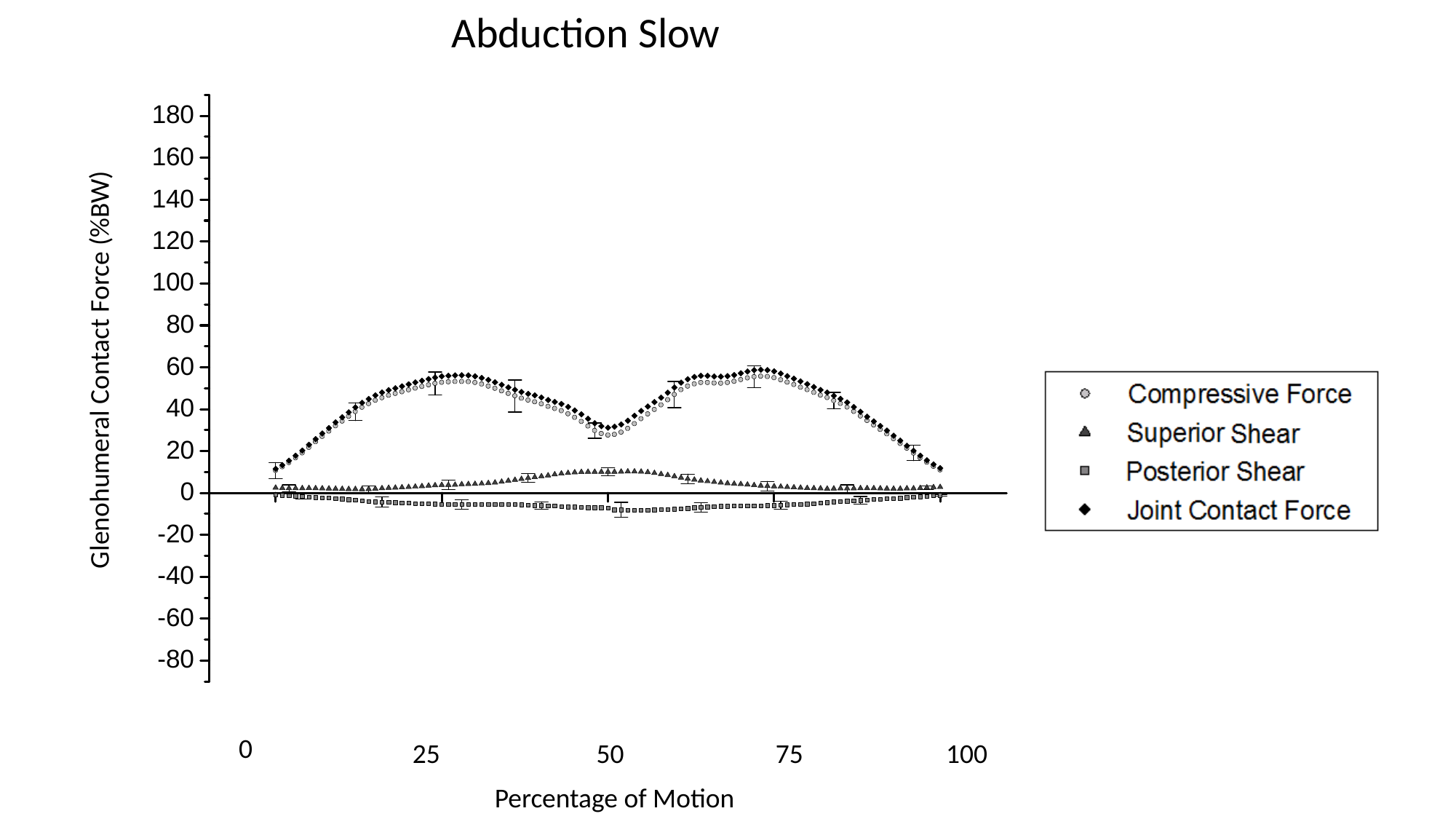

Abduction Slow
Glenohumeral Contact Force (%BW)
0
25
50
75
100
Percentage of Motion

## Slide 25
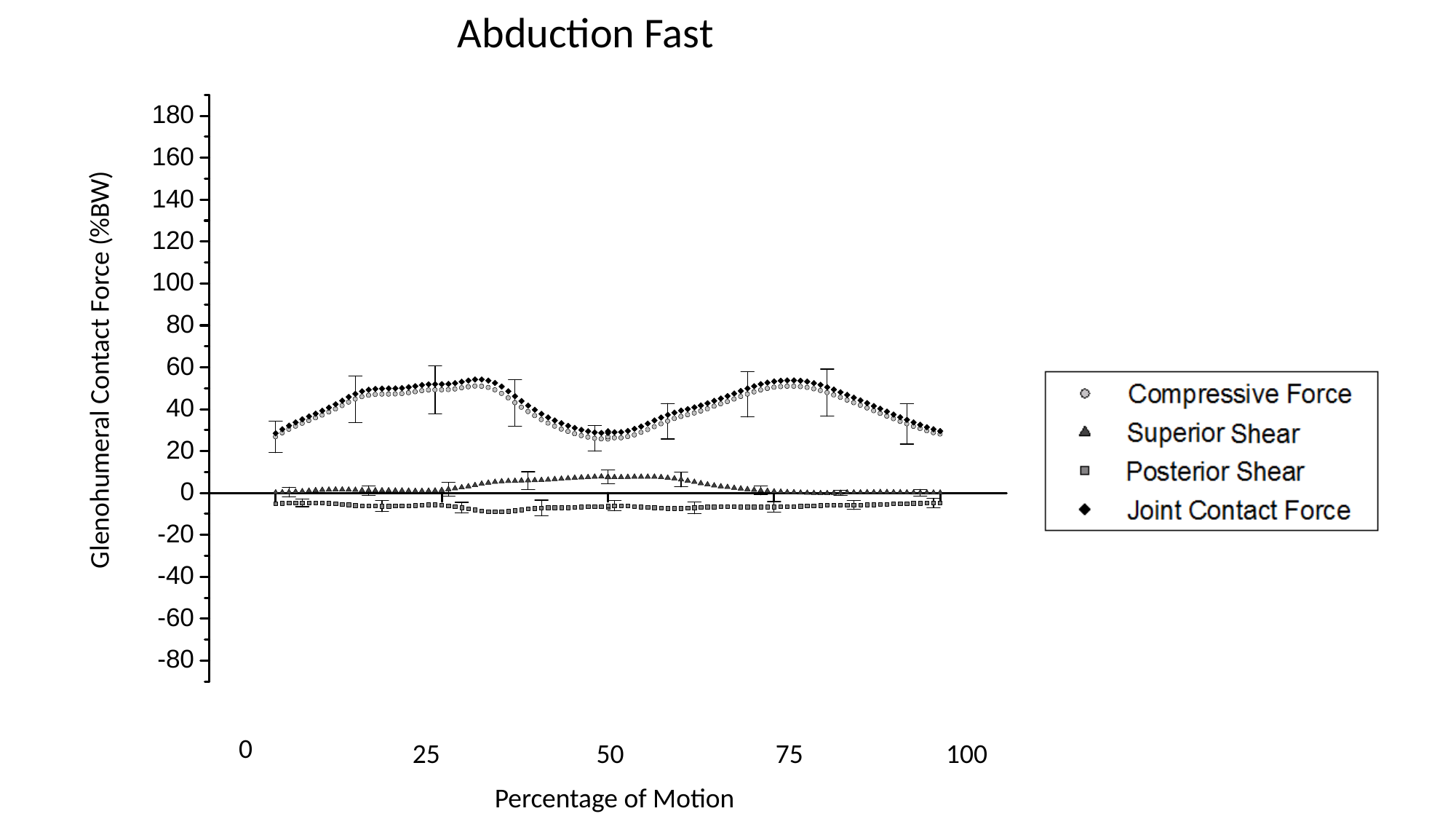

Abduction Fast
Glenohumeral Contact Force (%BW)
0
25
50
75
100
Percentage of Motion

## Slide 26
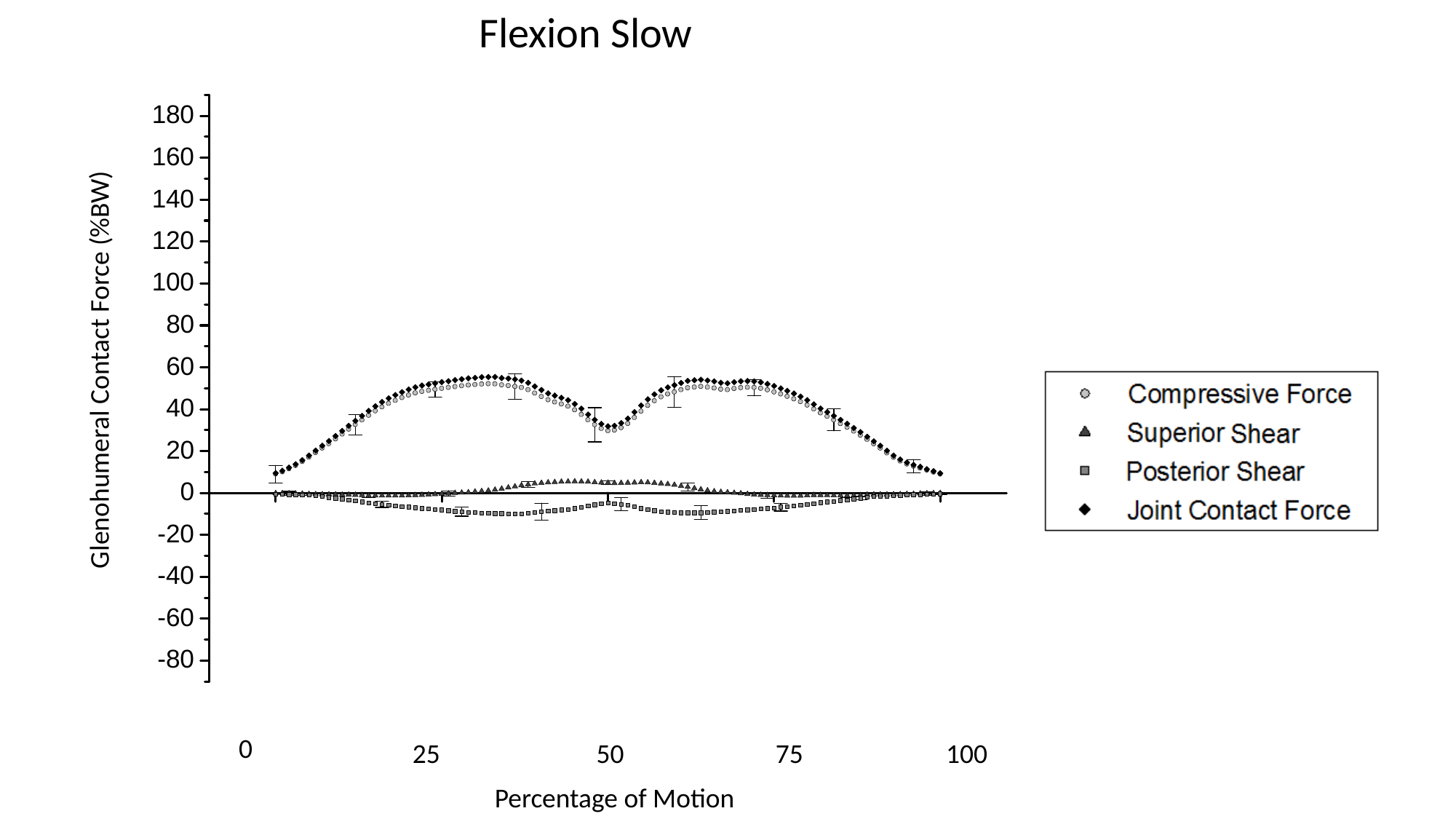

Flexion Slow
Glenohumeral Contact Force (%BW)
0
25
50
75
100
Percentage of Motion

## Slide 27
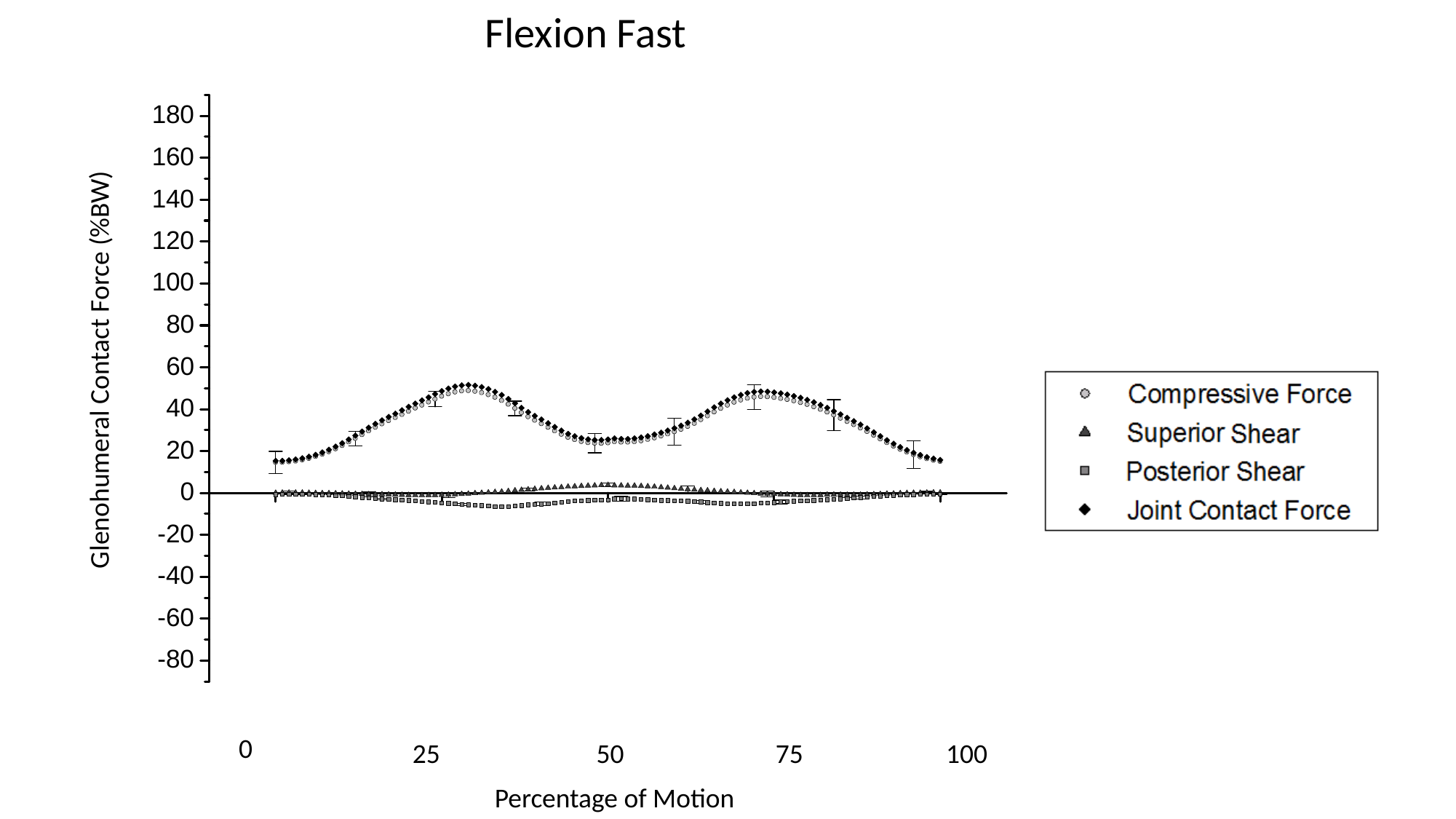

Flexion Fast
Glenohumeral Contact Force (%BW)
0
25
50
75
100
Percentage of Motion
